# Design, Synthesis, and Evaluation of New Mesenchymal–Epithelial Transition Factor (c-Met) Kinase Inhibitors with Dual Chiral Centers

**DOI:** 10.3390/molecules27175359

**Published:** 2022-08-23

**Authors:** Han Yao, Yuanyuan Ren, Jun Yan, Jiadai Liu, Jinhui Hu, Ming Yan, Xingshu Li

**Affiliations:** 1 School of Pharmaceutical Sciences, Sun Yat-sen University, Guangzhou 510006, China; 2 School of Biotechnology and Health Sciences, Wuyi University, Jiangmen 529020, China

**Keywords:** c-Met inhibitors, tepotinib, chiral compounds, kinase, cancer

## Abstract

A series of tepotinib derivatives with two chiral centers was designed, synthesized, and evaluated as anticancer agents. The optimal compound **(*R*, *S*)-12a** strongly exhibited antiproliferative activity against MHCC97H cell lines with an IC_50_ value of 0.002 μM, compared to tepotinib (IC_50_ = 0.013 μM). Mechanistic studies revealed that compound **(*R, S*)-12a** significantly inhibited c-Met activation, as well as the downstream AKT signaling pathway, and suppressed wound closure. Moreover, compound **(*R, S*)-12a** induced cellular apoptosis and cell cycle arrest at the G_1_ phase in a dose-dependent fashion.

## 1. Introduction

Cellular mesenchymal–epithelial transition factor (c-Met)—a high-affinity receptor for the hepatocyte growth factor—is a unique subfamily of receptor tyrosine kinases [1,2,3]. The c-Met signaling pathway is vital in invasive growth during embryonic development and postpartum organ regeneration [4,5,6]. Normally, in adults, the c-Met signaling pathway is fully activated only in the process of wound healing and tissue regeneration; however, the tumor c-Met signaling pathway can be frequently activated by cancer cells to promote tumor formation, invasive growth, and metastasis [7,8,9]. Studies have shown that the c-Met signaling pathway has an abnormal expression or mutation in various types of solid tumors, such as lung, gastric, liver, breast, skin, and colorectal cancers [10,11]. The c-Met signaling pathway plays a vital role in the occurrence and development of a variety of tumors [12,13]. It is becoming a promising target for cancer treatment due to its status as a proto-oncogene and the disorders associated with poor prognosis [14,15].

**Figure 1:**
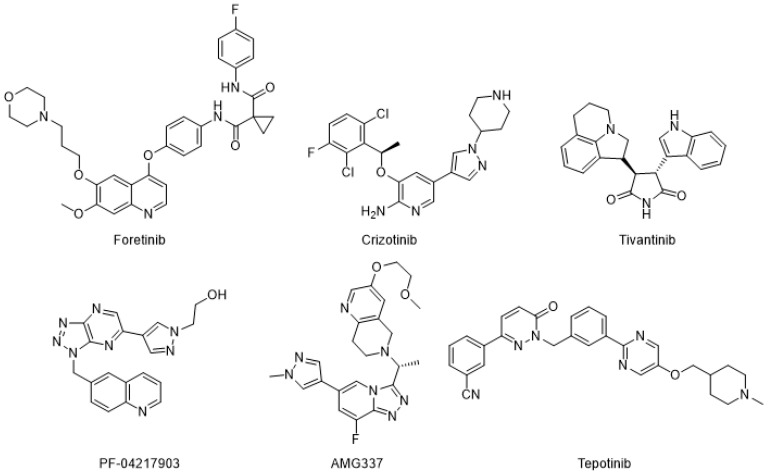
Structures of representative c-Met inhibitors.

Recently, different small-molecule c-Met inhibitors with different binding modes and selectivity profiles have been developed and have progressed to clinical trials, and some of them have been approved by the FDA for cancer treatment (Figure 1). A fast-tracked FDA approval was given to crizotinib, a potent c-Met/ALK dual inhibitor that demonstrated significant efficacy for a subset of NSCLC patients with an EML4-ALK fusion gene [16,17]. Tivantinib (ARQ-197), belonging to non-ATP competitive small molecule c-Met inhibitors, shows good antitumor activity in vitro and in vivo [18]. Foretinib, a type II multitargeted c-Met/VEGFR2 inhibitor bound to the deep hydrophobic back pocket, demonstrated partial regression or stable disease in patients with papillary renal carcinoma [19]. PF-04217903 showed effective tumor growth inhibition in c-Met-dependent tumor models with good oral PK properties and an acceptable safety profile in preclinical studies and progressed to clinical evaluation in a Phase I oncology setting [20]. AMG 337 demonstrated nanomolar inhibition of Met kinase activity, desirable preclinical pharmacokinetics, and a significant Met-dependent mouse efficacy model [21]. In 2021, the FDA approved tepotinib (MSC2156119) with excellent in vitro potency, high-kinase selectivity, long half-life after oral administration, and in vivo antitumor efficacy at low doses [22,23].

According to the binding mode between small molecules and c-Met kinase, small molecule inhibitors are mainly divided into two categories [24,25,26]. Class I is a specific inhibitor of c-met kinase, which can competitively bind to the ATP binding domain with ATP in a U-type binding mode. The nitrogen-containing aromatic ring structure in the inhibitor helps to maintain the U-type binding mode with ATP. At the same time, the nitrogen atom on the aromatic ring and the amine group and carbonyl group connected to the ring can form a hydrogen bond interaction with met1160 and pro1158 in the hinge region of c-met kinase. In addition, the inhibitor can also generate a π-π stacking effect with the tyr1230 residue of the activation ring. The typical Class I inhibitors are crizotinib, capmatinib, volitinib, AMG337, and tepotinib [27].

Class II small molecules are often ATP-competitive, multi-target c-met kinase inhibitors with a relatively flexible structure, which can recognize the inactive “DFG out” conformation and bind with it [28]. The representative drugs are cabozantinib, foretinib, altiratinib, and merestinib [29].

**Figure 2:**
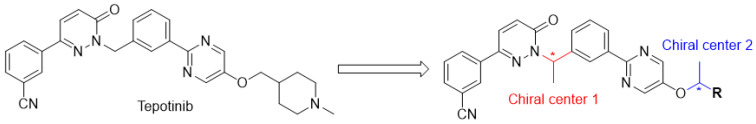
Design of compounds **12a-b**.

Although pharmaceutical scientists have extensively researched c-Met inhibitors, it is still necessary to find new antitumor compounds with good antitumor activity and minimum side effects from a theoretical and practical perspective [5]. Chirality is the basic feature of nature, and the main substances that make up life, such as amino acids, carbohydrates, nucleic acids, proteins, enzymes, and receptors, are chiral [30]. The pharmacological effects of drugs are achieved through strict recognition with macromolecules in the body [31]. In many cases, the pharmacological activity, metabolic process, metabolic rate, and toxicity of chiral compounds of different configurations in an organism have significant differences [32,33]. In addition, there are differences in absorption, distribution, and excretion of enantiomers [34,35]. Therefore, it is important to design and synthesize chiral compounds with different configurations and study their pharmacological activities [36]. Herein, inspired by the interesting scaffold of tepotinib and the importance of chirality in medicine, we have reported the design, synthesis, and activity study of tepotinib derivatives containing two chiral centers (Figure 2) to determine the leading compounds with good activity and minor side effects.

## 2. Results and Discussion

### 2.1. Chemistry

**Scheme 1:**
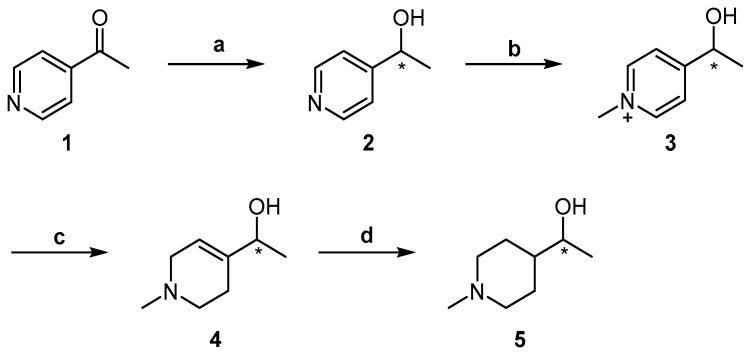
(a) (S,S,S)-CsDPEN/(R,R,S)-CsDPEN, [RuCl_2_(p-cymene)]_2_, HCOONa, DCM/H_2_O, 25 °C; (b) CH_3_I, CH_3_CN, 50 °C; (c) NaBH_4_, MeOH, 0 °C; (d) Pd/C, MeOH, 40 atm, 50 °C.

The synthetic routes of compounds **4** and **5** are shown in Scheme 1. Specifically, the asymmetric hydrogen transfer of 4-acetylpyridine with sodium formate afforded chiral alcohol **2**. Compound **2** reacted with methyl iodide, affording salt 3, which was subjected to reduction by sodium borohydride to give compound **4**. The catalytic hydrogenation reaction of compound **4** yielded N-methyl-1-methylpiperidine-4-methanol 5 [37].

**Scheme 2:**
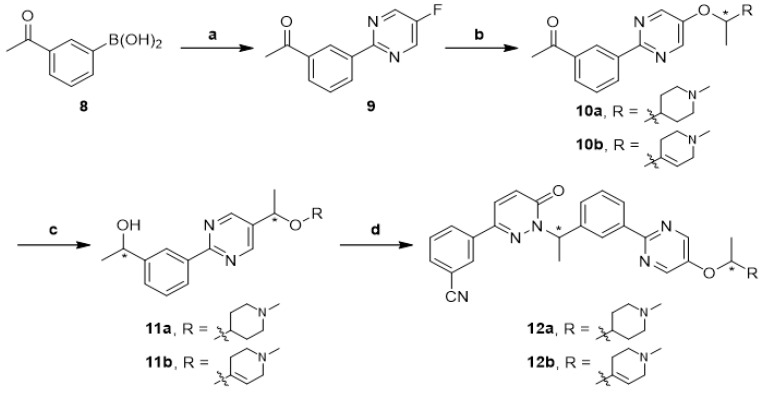
(a) 2-*Chloro*-5-*fluoro*pyrimidine, (Ph_3_P)_2_PdCl_2_, Na_2_CO_3_, PhMe/EtOH/H_2_O, N_2_, 83 °C; (b) compounds **4** and **5**, NaH, DMF, 0 °C; (c) [Ru(p-cymene)Cl_2_]_2_, (S,S,S)-Cs-DPEN/(R,**R,S**)-Cs-DPEN, HCOONa, DCM/H_2_O, room temperature; (d) compound **7**, DIAD, PPh_3_, THF, N_2_, room temperature. “*” refers a chiral center here.

The synthetic route of the target compound **12a-b** is shown in Scheme 2. The Suzuki cross-coupling reaction of 3-acetylphenylboronic acid with 2-chloro-5-fluoropyrimidine afforded compound **9**. The reaction of compound **9** with chiral alcohols 4 or 5 with sodium hydride yielded compounds **10a-b**. Asymmetric hydrogen transfer of compounds **10a-b** with the same procedure, using sodium formate and the chiral catalyst, yielded compounds **11a-b** [37]. Finally, the Mitsunobu reaction of chiral alcohols **11a-b** with 3-(6-oxo-1,6-dihydropyridazin-3-yl)benzonitrile **7** afforded the target compounds **12a-b**.

### 2.2. The Preperation of Monocrystal

The two chiral centers of compounds **12a-b** are formed through an asymmetric hydrogen transfer reaction, with (S, S, S)-CsDPEN/Ru or (R, R, S)-CsDPEN/Ru as the catalyst. We chose compound **R-5b**, containing a single chiral center, to determine its absolute configuration. The method for preparing a single crystal of **R-5b** is as follows: Compound **R-5b** (506.6 mg, 1 mmol) was dissolved in freshly re-distilled absolute ethanol. 1 mL of hydrochloric acid standard solution in ethyl acetate (concentration of 1 mmol/mL) was added dropwise into the **R-5b** solution and then stirred for 1 h. The excess solvent is removed by rotary evaporation to obtain a pale-yellow solid as the hydrochloride of compound **R-5b**. Then, it was dissolved in 5 mL of re-distilled dichloromethane, and 15 mL of re-distilled ethyl acetate was slowly added. After standing still for 3 days, pale-yellow needle-like crystals are precipitated, which are used for X-ray single-crystal diffraction.

### 2.3. The Result of X-ray Diffraction

**Figure 3:**
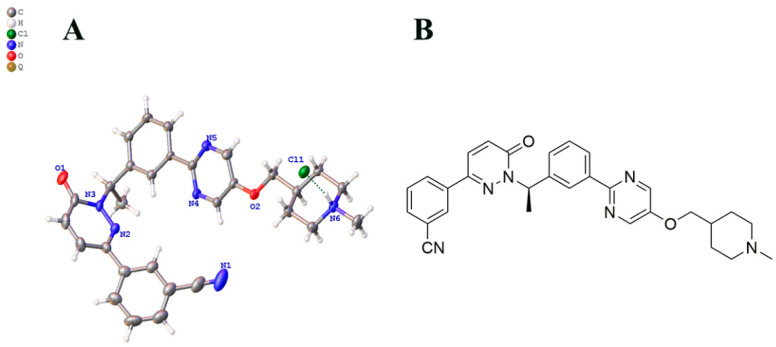
(**A**). Structure of compound **R-5b** analyzed by X-ray diffraction. (**B**). The absolute configuration of compound **R-5b**.

In the synthesis of compound **R-5b**, the S-configuration ligand was used to catalyze the asymmetric hydrogen transfer reaction and to obtain the S-configuration alcohol. During the Mitsunobu reaction in the last step, the inversion of the configuration occurred. Therefore, using the (S, S, S)-CsDPEN ligand in the synthesis resulted in an R-configuration final product. The results of X-ray single crystal diffraction analysis (Figure 3) are consistent with the above inference. The X-ray statistics of **(R)-5b** can be fetched in the CSDS database with the deposition number of 2,132,093 [38].

### 2.4. In Vitro Antiproliferative Activities and SARs

In China, hepatocellular carcinoma is one of the leading cancers that endanger human lives. In our previous work, we synthesized a serial of compounds with mono chiral centers and investigated the effect of chiral moiety on biological activities [39].

On the basis of our former study, compounds **12a-b** were designed and first screened for their antiproliferative activity against the c-Met-driven human hepatocarcinoma cells (MHCC97H), using a standard MTT assay [40]. Tepotinib was used as a positive control.

The IC_50_ values of compounds **12a-b** and the optimal compounds, bearing the single chiral center of previous work, are listed in Table 1. Generally, the derivatives with two chiral centers demonstrated potent antiproliferative activities with the IC_50_ values ranging from submicromolar to nanomolar levels. First, we examined the activities of compound **12a**, in which the R group was a 1-(1-methylpiperidin-4-yl)ethan-1-yl moiety. Compound *(**R, R**)***-12a** showed both chiral carbons in the R configuration and exhibited potent activity against MHCC97H with the IC_50_ value of 0.035 μM. Second, compared to **(*R, R*)-12a**, compound **(*R, S)-*12a** keeps the configuration of C1 carbon as an R configuration and changes the C2 to an S configuration, showing the most potent antiproliferative activity against MHCC97H. The IC_50_ value of compound ***(R, S)-*12a** was 0.0021 μM, which is 10-fold more than the former compound **(*R, R)-*12a**.

**Figure 4:**
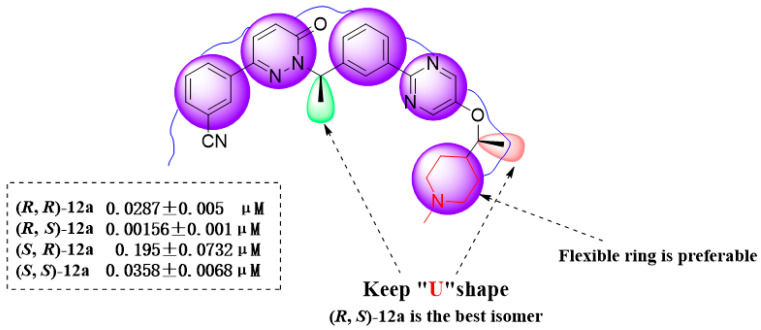
SAR for compounds **12a-b**.

However, the antiproliferative activities of the compounds **(*S, R*)-12a** and **(*S, S*)-12a** are the opposite. The IC_50_ value was 0.2052 and 0.0483 μM for compounds **(*S, R*)-12a** and **(*S, S*)-12a**, respectively. The former study showed that C1 with an R configuration presented better antitumor activities. These results indicated that the configurations of the two chiral carbons are essential for antitumor activities, and the best configuration combination improves biological activities. The optimum results should be such that C1 is in the R configuration, and C2 is its opposite. Third, compound **12b**, featuring 1-(1-methyl-1,2,3,6-tetrahydropyridin-4-yl)ethan-1-yl moiety as the R group, displayed considerably lower antiproliferative activities than the corresponding analog **12a**. These results indicated that the double bond of the R group is unfavorable to antiproliferative activities. The changes in the antiproliferative activities of compounds **12a-b** have the same relationship with the configuration of a chiral carbon (Figure 4). The above results indicate that only compounds with a specific configuration can better bind to the target and achieve an excellent antitumor effect.

### 2.5. Toxicity towards Normal Cells

To evaluate the selectivity of **(*R, S*)-12a** to MHCC-97H and human normal cells, two types of human normal cells including HaCat (human immortalized keratinocytes) and LO2 (human normal liver cells) were used to test the selectivity of **(*R, S*)-12a** toward other cancer cells and normal cells, while tepotinib was used as a positive control. As shown in Table 2, compound **(*R, S*)-12a** exhibited high cytotoxicity toward hepatic carcinoma MHCC-97H cells and was nearly inactive in the inhibition of human normal cells HaCat and LO2, with selectivity ratios of 953.5 and 5750.0, respectively, which were superior to that of tepotinib (150.08 and 671.54).

### 2.6. Effects of Compound **(R, S)-*12a*** on Tumor Cell Morphology

**Figure 5:**
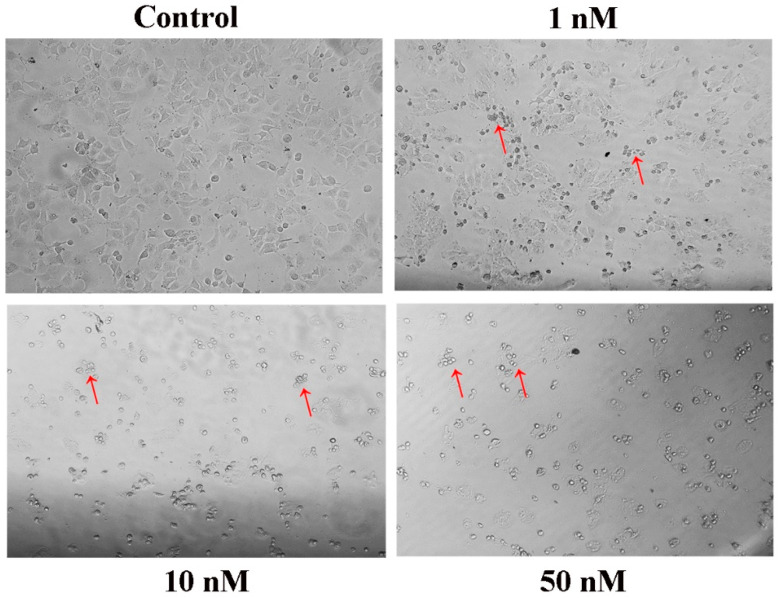
Effects of compound **(*R*, *S*)-12a** on tumor cell morphology.

As the compound ***(R, S)-*12a** exhibited excellent antiproliferative activity in the study of the relationship between structure and activity, it was chosen for studying the effect on the morphology of MHCC-97H tumor cells. As shown in Figure 5, the control group showed the normal morphology of adherence. Co-incubation with compound **(*R*, *S*)-12a** (1, 10, and 50 nM) led to a significant reduction in loss of cell viability. Red arrowheads indicated distinct morphological changes, such as intracytoplasmic particles increasing, the cell shape change, and loss of adherent property. Under the treatment of the compound **(*R*, *S*)-12a** at the concentrations of 10 and 50 nM, the cells shrank and floated and no longer adhered to the wall. Aggregation, lysis, and cell debris also occurred.

### 2.7. Immunofluorescence Staining and Western Blotting

**Figure 6:**
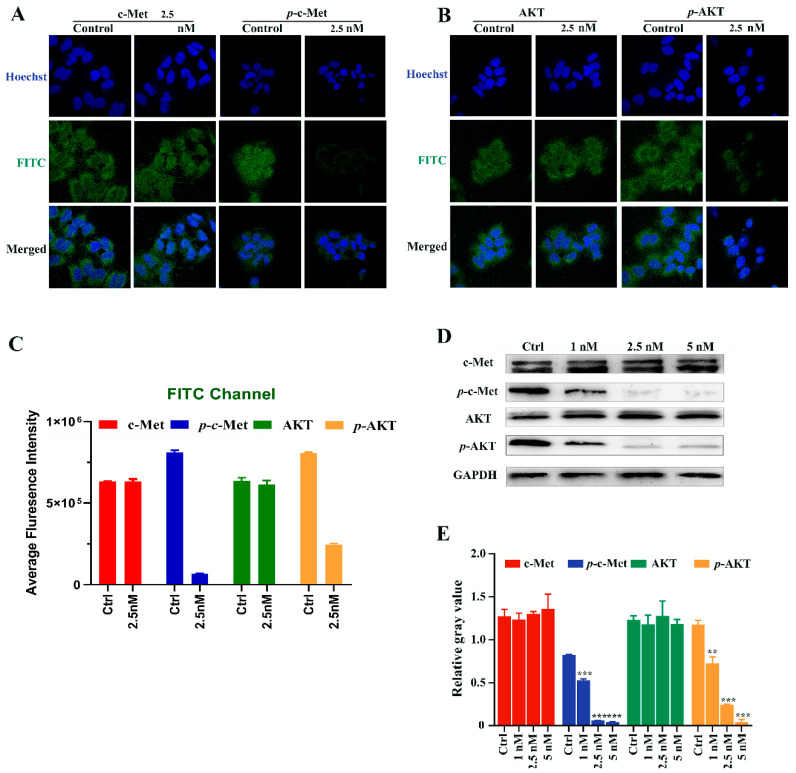
(**A**,**B**) Immunofluorescence staining analysis for the expression of c-Met, p-c-Met, AKT, and p-AKT. MHCC97H cells were treated with vehicle control (0.1% DMSO) and compound **(*R, S*)-12a** for 24 h. The expression of c-Met, p-c-Met, AKT, and p-AKT was visualized with FITC antibody, AlexaFluor488 (green), and counterstained with Hoechst 33342 (blue) via confocal microscopy. (**C**) The average fluorescence intensity of FITC channel (**A**,**B**). (**D**) Western blot images. (**E**) Histogram of relative gray value in 6D, which is quantified by Image J and analyzed by GraphPad Prism 8. “**”, *p* < 0.01, “***”, *p* < 0.001.

To study the effect of the compound ***(R, S)-*12a** on the phosphorylation level of c-Met and Akt proteins in tumor cells, we performed immunofluorescence and Western blot assays. As shown in Figure 6A–C, after the MHCC97H cells were treated with compound **(*R, S*)-12a** (2.5 nM) for 48 h, immunofluorescence assays were performed using confocal microscopy. Staining the nucleus with Hoechst 33,342 and labeling the target protein with FITC and confocal laser assay showed that the target proteins c-Met, p-c-Met, AKT, and p-AKT were all located outside the nucleus. After **(*R, S*)-12a** treatment at a concentration of 2.5 nM, the fluorescence intensity of c-Met and AKT in the cells did not change significantly, that is, the total amount of c-Met and AKT proteins did not change significantly, but the fluorescence intensity of p-c-Met and p-AKT decreased significantly. In addition, we examined the expression levels of c-Met, p-c-Met, AKT, and p-AKT proteins via immunoblotting analysis. As shown in Figure 6D,E, compound **(*R, S*)-12a** was found to decrease the phosphorylation level of c-Met and AKT protein in a dose-dependent fashion, which is consistent with the results obtained by immunofluorescence assays. These results implied that compound **(*R, S*)-12a** exhibits an effective inhibition of c-Met activation and downstream signaling pathway AKT [41,42,43].

### 2.8. Cell Cycle Study

**Figure 7:**
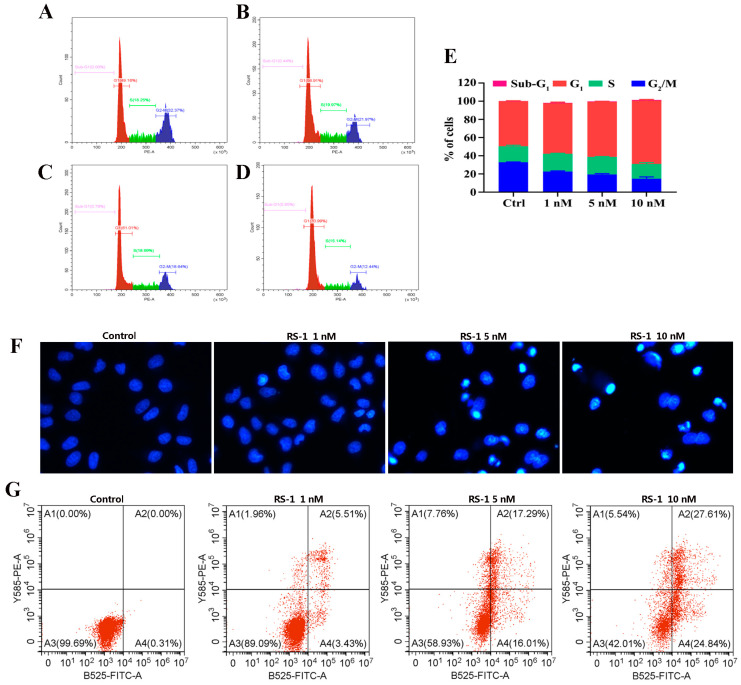
Compound ***(R, S)-*12a** induced G_1_ phase arrest in MHCC97H cells. The MHCC97H cells were treated with compound ***(R, S)*-12a** for 48 h, harvested, stained with PI, and analyzed by flow cytometry. (**A**) Vehicle control; (**B**–**D**) compound ***(R, S)-*12a** at the concentrations of 1, 5, and 10 nM, respectively; (**E**) histograms showing the proportions of cell distribution; (**F**) Hoechst 33342 staining of MHCC97H cells with the treatment of compound **(*R, S)-*12a** (1, 5, and 10 nM) for 48 h. (**G**) Representative flow cytometric profiles showing that compound ***(R, S)-*12a** (1, 5, and 10 nM) induces MHCC97H cells apoptosis for 48 h.

In the next study, compound **(*R*, *S*)-12a** was examined for its effect on the cell cycle progression of MHCC-97H tumor cells by a flow cytometry analysis. As shown in Figure 7, upon the treatment with compound **(*R*, *S*)-12a** at the concentrations of 1, 5, and 10 nM, the growth cycle changed significantly in a dose-dependent fashion. The proportions of cells in the G_1_ phase increased, and the proportions of cells in the G_2_/M phase decreased. The effect was most obvious for compound **(*R, S*)-12a** at the concentration of 10 nM: the proportion of cells in the G_1_ phase increased from 49.16% to 70.99%, and the proportion in the G_2_/M phases decreased from 32.37% to 12.44%, compared to the vehicle control. These results indicated that compound **(*R, S*)-12a** could trigger cell cycle arrest in the G_1_ phase.

### 2.9. Cellular Apoptosis Analysis

Tumor cell apoptosis is an important stage in the effective treatment of cancer [33]. To evaluate the capacity of compound **(*R, S*)-12a** to induce apoptosis in human cancer cell lines, we used Hoechst 33342 staining to investigate the effect of compound **(*R, S*)-12a** on MHCC97H cells. Hoechst 33342 staining was used to examine apoptosis through morphological observation. As shown in Figure 7F, obvious nucleolar pyknosis, DNA fragmentation, and chromatin condensation were observed in the compound **(*R, S*)-12a**-treated cells with stronger blue fluorescence compared to the vehicle control cells.

Moreover, an FITC conjugated Annexin-V/PI assay was performed with MHCC-97H tumor cells, and the results are shown in Figure 7G. Upon the treatment with compound **(*R, S*)-12a**, the proportions of early and late apoptosis cells were 8.9% at 1 nM, 33.3% at 5 nM, and 52.5 at 10 nM. These results implied that compound **(*R, S*)-12a** could effectively induce apoptosis in a dose-dependent manner and thus lead to the death of cancer cells eventually.

### 2.10. **(R, S)-*12a*** Inhibited MHCC-97H Tumor Cell Migration

**Figure 8:**
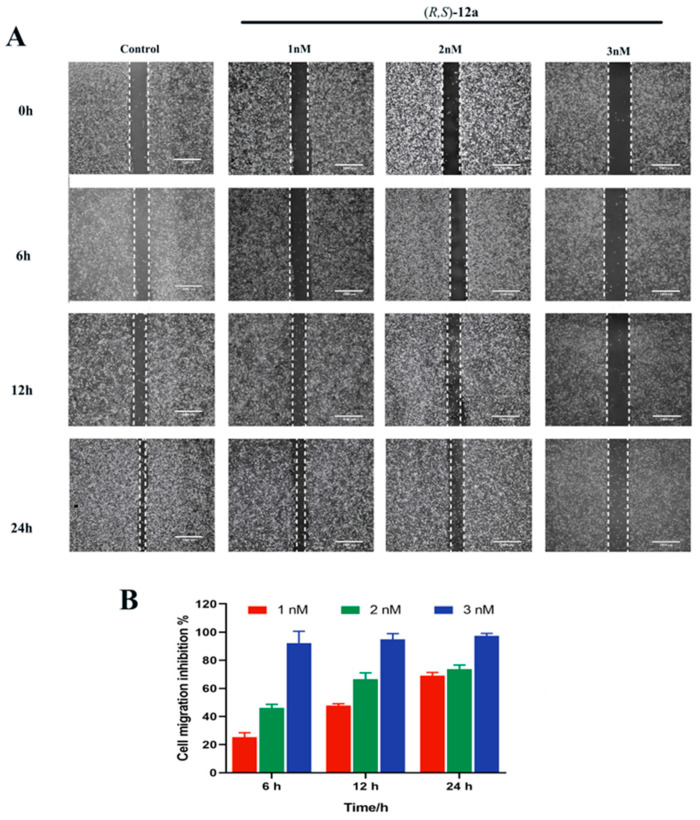
Effects of compound **(*R***, ***S*)-12a** on MHCC97H cell migration. (**A**) Representative images of the wound area. Images were obtained at 0, 6, 12, and 24 h after treatment with 1% DMSO and compound **(*R***, ***S*)-12a** (1, 2, and 3 nM) by phase-contrast microscopy. (**B**) Histograms displaying the cell migration inhibition of compound **(*R***, ***S*)-12a** (1, 2, and 3 nM).

Tumor metastasis is one of the main causes of patient death [34]. To investigate whether the compound **(*R***, ***S*)-12a** could inhibit tumor cell migration, we conducted a cell scratch assay in MHCC97H cells. As shown in Figure 8, compound **(*R***, ***S*)-12a** significantly suppressed wound closure in a dose- and time-dependent manner, compared to the vehicle control. These results suggest that compound **(*R***, ***S*)-12a** may have the potential ability to inhibit tumor cell migration and metastasis.

### 2.11. Computational Characterization of the Binding Modes between Compounds ***12a*** and c-Met

**Figure 9:**
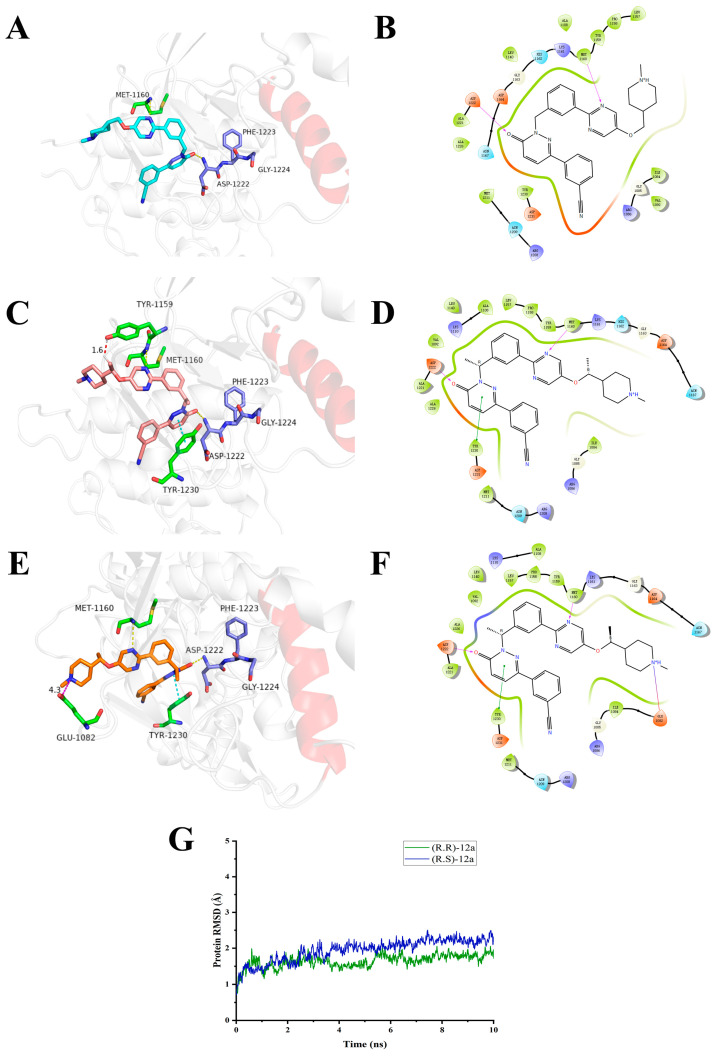
(**A**) Tepotinib is in cyan; motif 1222, 1223, and 1224 of DFG catalytic triplet are in purple blue; C-helices are in red. (**B**) The 2D interaction of tepotinib. (**C**) **(*R*, *R*)-12a** is in violet; motif 1222, 1223, and 1224 of DFG catalytic triplet are in purple blue; C-helices are in red. (**D**) The 2D interaction of **(*R*, *R*)-12a**. (**E**) **(*R*, *S*)-12a** is in orange; motif 1222, 1223, and 1224 of DFG catalytic triplet are in purple blue; C-helices are in red. (**F**) The 2D interaction of **(*R*, *S*)-12a**. (**G**) RMSD and plots of protein backbone atoms for c-met structures in complex with **(*R*, *S*)-12a**, **(*R*, *R*)-12a**.

To explore the possible binding mode of compounds **(*R***, ***R*)-12a** and **(*R***, ***S*)-12a** with c-Met, we used the crystal structure of c-Met as a template (tepotinib in PDB:4R1V) [22], and a docking operation was conducted to rationalize the inhibition results with Schrödinger suites (Figure 9). Comparing the binding mode of **(*R***, ***R*)-12a** and **(*R***, ***S*)-12a** with tepotinib, we found both of them were type I inhibitors, the same as tepotinib. They occupy c-Met’s ATP-binding site and compete with ATP molecules by forming hydrogen bonds with Met 1160 and Asp 1222 motifs and also bind to the active region of c-Met, for the c-helix and DFG motifs (a usual catalytic triplet in a kinase structure) that adopt an “in” configuration [43].

Unlike tepotinib, the introduction of a methyl group, which constructs the C2 chiral center, produces a π–π stacking interaction with Tyr 1230, enhancing the inhibitory activity. Moreover, the methyl group of the C2 chiral center of the two enantiomers induces N-methylpiperazine moiety in a different direction. Figure 9C shows the direction of the methyl group in **(*R***, ***S*)-12a** that created a salt bridge between Glu1082 and the nitrogen atom on the piperidine ring. In contrast, the methyl group of **(*R***, ***R*)-12a** pointed toward TYR1159, in a distance of 1.6 Å, which is a steric hindrance (Figure 9B). This could explain why the compounds in R configuration of the C2 chiral center are less active than in S configuration.

### 2.12. Molecular Dynamics Simulations

To further study the stability of the docking complex to the target, molecular dynamics (MD) simulations were performed preliminarily, and the protein RMSD values for each simulation run were monitored to measure the stability of protein–ligand poses. As the backbone RMSD value of two complexes in Figure 9, the RMSD value of the system for the **(*R*, *S*)-12a** complex increased to around 2.0 Å at 4 ns and settled smoothly around 2.2 Å until the end of the simulation, while the RMSD plot of **(*R*, *R*)-12a** demonstrated the initial complex system fluctuations till 5.5 ns, and then the plot stabilized around 1.7 Å, indicating that the system equilibrated at the end of the simulation. These results indicated that the conformational changes of the docked complexes were within the acceptable limit of 1–3 Å during the 10 ns simulation [44].

## 3. Discussion

At present, the total number of drugs used in the world is about 1900. Among them, about 200 drugs are commonly used in clinical practice, and there are more than 110 kinds of chiral drugs. The discovery of chiral drugs is still one of the main directions of new drug research. In this paper, we developed a series of tepotinib derivatives with two chiral centers. Studies on cell proliferation activities show that the configuration of chiral carbon in the molecular structure has a great influence on antiproliferative activities. The antiproliferative activities of compounds with different configurations (**(*R*, *S*)-12a** vs. **(*S***, ***R*)-12a**) differ by nearly a hundred-fold. These results indicate that the optimal compound **(*R*, *S*)-12a** is worthy of further druggability studies.

## 4. Materials and Methods

### 4.1. Chemistry

#### 4.1.1. General Methods and Materials for Synthesis

All reagents used in the synthesis were obtained commercially and used without further purification unless otherwise specified. The ^1^H NMR and ^13^C NMR spectra were recorded using TMS as the internal standard on a Bruker BioSpin GmbH spectrometer at 400/500 and 100/125 MHz, respectively. High-resolution mass spectra (HR-MS) were recorded using a Shimadzu LCMS-ITTOF mass spectrometer (more data at Supplementary Materials).

The reactions were monitored by thin-layer chromatography (TLC) on glass-packed precoated silica gel plates and visualized in an iodine chamber or with a UV lamp. Flash column chromatography was performed using silica gel (200–300 mesh) purchased from Qingdao Haiyang Chemical Co. Ltd. (Qingdao, China). Chemical and solvents were of reagent grade and used without further purification.

The purities of all the final synthesized compounds were ≥95% as determined by high-performance liquid chromatography (HPLC), compound **12a** with an Agilent TC-C18 column (4.6 × 250 mm, 5 μm) and compound **12b** with Shimadzu GIST-HP C-18 column (2.1 × 100 mm, 3 μm). The enantiomeric excess of compounds **(*R*, *S*)-12b** and **(*S*, *R*)-12b** was analyzed using Daicel OJ-H column, eluted with n-hexane/isopropanol (85/15, *v*/*v*) and 1‰ diethylamine as a tailing agent at a low flow rate of 0.5 mL/min. The chiral purities of the other target chiral compounds were analyzed using Daicel AD-H column, eluted with n-hexane/isopropanol (90/10, *v*/*v*) and 1‰ diethylamine as a tailing agent at a low flow rate of 0.5 mL/min.

The preparation of 3-(6-*oxo*-1,6-dihydropyridazin-3-yl)benzonitrile (7) was carried out according to the method of literature.

#### 4.1.2. General Procedures for Preparation of Compounds **(*R*)-2** and **(*S*)-2**

A suspension formed by mixing of chiral ligand (*S*, *S*, *S*)-CsDPEN/(*R*, *R*, *S*)-CsDPEN (0.23 mmol, 96 mg) and [RuCl_2_(p-cymene)]_2_ (0.11 mmol, 64.5 mg) in water (30 mL) was stirred at 40 °C for 4 h under nitrogen. To the chiral catalyst prepared above, a solution of 4-acetylpyridine and sodium formate (32.3 mmol, 2.2 g) in dichloromethane/water (30 mL) was added. The reaction mixtures were stirred at room temperature and monitored with TLC. After 20 h, the organic phase was separated, washed with brine, dried, and concentrated in vacuo. The crude products were purified with silica gel column chromatography using dichloromethane/methanol (10/1, *v*/*v*) as eluents to afford compounds **(*R*)-2** and **(*S*)-2**. Colorless oil, yield: 73.4%. ^1^H NMR (400 MHz, CDCl_3_) δ 8.51–8.27 (m, 2H), 8.11 (s, 1H), 7.79–7.57 (m, 1H), 6.79 (s, 1H), 4.47 (m, 1H), 3.02 (s, 3H).

#### 4.1.3. General Procedures for Preparation of Compounds (***R***)-**4** and (***S***)-**4**

To a solution of compound **(*R*)-2** (9.2 mmol, 1.19 g) in acetonitrile (15 mL), methyl iodide (12.1 mmol, 0.75 mL) was added. The reaction mixture was stirred at 40 °C and checked with TLC to confirm that the reaction was completed. After 3.5 h, the solvents were removed under reduced pressure. The crude product, crude **(*R*)-3**, was used for the next step. To a solution of compound **(*R*)-3** in methanol (25 mL), sodium borohydride (37.0 mmol, 1.4 g) was added in batches under an ice bath. The reaction mixture was stirred for one hour and then slowly warmed to room temperature and checked with TLC. The reaction was quenched with water (30 mL), and the mixture was extracted with ethyl acetate (30 × 2 mL). The organic phase was separated, washed, dried, and concentrated under reduced pressure to provide compounds **(*R*)-4** and **(*S*)-4**. Brown oil, yield: 85.5%. ^1^H NMR (400 MHz, CDCl_3_) δ 5.61 (dq, *J* = 3.1, 1.5 Hz, 1H), 4.18 (q, *J* = 6.3 Hz, 1H), 2.99 (s, 1H), 2.96 (d, *J* = 3.0 Hz, 2H), 2.63–2.51 (m, 2H), 2.36 (s, 3H), 2.21 (m, 2H), 1.25 (d, *J* = 6.5 Hz, 3H).

#### 4.1.4. General Procedures for Preparation of Compounds **(*R*)-5** and (***S***)-5 [11]

To a solution of chiral alcohol **(*R*)-4** (8 mmol; 1.14 g) in methanol (6 mL) in an autoclave, palladium on carbon (1.6 mmol, 170 mg) was added. The reaction system was replaced with hydrogen three times, pressurized to 40 bar, and then stirred at 50 °C, monitored by the iodine cylinder. After 24 h, the mixture was concentrated under reduced pressure to afford compound **(*R*)-5**. Colorless oil, yield: 90.6%. ^1^H NMR (400 MHz, CDCl_3_) δ 3.58 (p, *J* = 6.3 Hz, 1H), 2.99–2.89 (m, 2H), 2.30 (s, 3H), 2.01–1.91 (m, 2H), 1.87 (dt, *J* = 13.0, 2.7 Hz, 1H), 1.64 (dt, *J* = 13.0, 3.0 Hz, 1H), 1.41 (m, 2H), 1.32–1.22 (m, 1H), 1.18 (d, *J* = 6.3 Hz, 3H).

##### 1-(3-(5-Fluoropyrimidin-2-yl)phenyl)ethan-1-one (**9**)

A mixture of sodium carbonate (66 mmol, 6.72 g), 5-fluoro-2-chloropyrimidine (33 mmol, 4.32 g), (Ph_3_P)_2_PdCl_2_ (0.68 mmol, 0.48 g), 3-acetylphenylboronic acid (32 mmol, 5.25 g) in a mixture of toluene (65 mL), ethanol (33 mL), and water (32 mL) was refluxed at 83 °C for 11 h under nitrogen. After cooling to room temperature, the reaction mixture was extracted with ethyl acetate. The organic phase was separated, dried, and concentrated in vacuo. The crude product was slurried using petroleum ether/ethyl acetate (8:1, *v*/*v*) and filtered to obtain compound **9**. Light yellow solid, yield: 80.2%. ^1^H NMR (400 MHz, CDC1_3_) δ 8.98 (t, *J* = 1.6 Hz, 1H), 8.70 (s, 2H), 8.59–8.47 (m, 2H), 7.60 (m, 1H), 2.71(s, 3H).

##### (***S***,***R***)-1-(3-(5-(1-((1-Methyl-1,2,3,6-tetrahydropyridin-4-yl)oxy)ethyl)pyrimidin-2-yl)phenyl)ethan-1-ol (**11**)

To a mixture of compound **(*R*)-5** or **(*S*)-5** in DMF (10 mL), sodium hydride (60%, 3.80 g) was added in batches at 0 °C. After 15 min, compound **9** (0.59 mmol, 1.28 g) in DMF (13 mL) was slowly added to the above mixture at 0 °C. The mixture was allowed to warm to room temperature. After 1.5 h, the reaction mixture was slowly poured into ice water (30 mL) and extracted with dichloromethane (60 mL). The organic layer was separated and washed with water three times to remove the DMF to obtain compound **10** in dichloromethane. A suspension formed by mixing chiral ligand (*S*,*S*,*S*)-CsDPEN/(*R*,*R***,***S*)-CsDPEN (0.23 mmol, 96 mg) and [RuCl_2_(p-cymene)]_2_ (0.11 mmol, 64.5 mg) in water (30 mL) was stirred at 40 °C for 4 h under nitrogen. To the chiral catalyst prepared above, a solution of compound **10** and sodium formate (13.1 mmol, 0.89 g) in dichloromethane/water (30 mL) was added. The reaction mixtures were stirred at room temperature and monitored with TLC. The reaction mixtures were stirred at room temperature and monitored with TLC. After 20 h, the organic phase was separated, washed with brine, dried, and concentrated in vacuo. The crude products were purified with silica gel column chromatography using dichloromethane/methanol (30/1→10/1, *v*/*v*) as eluents to afford compounds **(*S*,*R*)-11**. Brown oil, yield: 75.5%. ^1^H NMR (400 MHz, CDCl_3_) δ 8.44 (s, 2H), 8.33 (s, 1H), 8.24 (d, *J* = 6.9 Hz, 1H), 7.48 (d, *J* = 7.2 Hz, 2H), 5.75 (s, 1H), 4.81 (q, *J* = 6.1 Hz, 1H), 2.94 (q, *J* = 16.6 Hz, 2H), 2.60 (dt, *J* = 10.6, 5.1 Hz, 1H), 2.43 (dt, *J* = 11.5, 5.9 Hz, 2H), 2.32 (s, 3H), 2.26 (s, 1H), 2.13 (d, *J* = 17.4 Hz, 1H), 1.55 (d, *J* = 6.5 Hz, 3H), 1.52 (d, *J* = 6.4 Hz, 3H).

##### General Procedures for Preparation of Compounds **12a-b**

To a solution of compound **7** (1.5 mmol, 296 mg), triphenylphosphine (3.0 mmol, 715 mg), and chiral alcohol intermediates **11** (1.3 mmol, 445 mg) in anhydrous tetrahydrofuran (4 mL) diisopropylazodicarboxylate (551 mg, 2.72 mmol) was added dropwise under nitrogen. The mixture was allowed to warm to room temperature and stirred overnight. The reaction mixtures were quenched with ice water, and the mixture was extracted with ethyl acetate (25 mL). The organic layers were separated, dried, and concentrated under reduced pressure. The crude products were purified by silica gel column chromatography using ethyl acetate/petroleum ether/triethylamine (100/25/1, *v*/*v*/*v*) as eluents to afford compounds **12a-b**.

##### (***R***,***R***)-3-(1-(1-(3-(5-(1-((1-Methylpiperidin-4-yl)oxy)ethyl)pyrimidin-2-yl)phenyl)ethyl)-6-oxo-1,6-dihydropyridazin-3-yl)benzonitrile ((***R***, ***R***)-**12a**)

Light yellow solid, yield: 43.2%. 1H NMR (400 MHz, Chloroform-d) δ 8.70 (s, 1H), 8.51 (s, 2H), 8.30 (d, *J* = 7.8 Hz, 1H), 8.23 (s, 1H), 8.00 (d, *J* = 8.0 Hz, 1H), 7.71 (d, *J* = 7.7 Hz, 1H), 7.63–7.54 (m, 3H), 7.45 (t, *J* = 7.7 Hz, 1H), 7.05 (d, *J* = 9.7 Hz, 1H), 6.57 (q, *J* = 7.1 Hz, 1H), 4.35 (p, *J* = 6.1 Hz, 1H), 2.99 (d, *J* = 11.2 Hz, 2H), 2.34 (s, 3H), 2.04–1.97 (m, 2H), 1.94 (d, *J* = 7.1 Hz, 3H), 1.75 (d, *J* = 12.0 Hz, 1H), 1.65– 1.49 (m, 2H), 1.36 (d, *J* = 6.2 Hz, 3H); 13C NMR (126 MHz) δ 159.24, 157.57, 150.28, 145.26, 141.88, 140.65, 137.69, 136.32, 132.51, 130.28, 129.83, 129.78, 129.73, 128.81, 128.74, 127.30, 126.92, 118.75, 113.32, 56.19, 54.59, 54.47, 43.82, 38.97, 25.74, 24.47, 20.11, 16.17, and HR-ESI-MS for C_31_H_32_N_6_O_4_ ([M + H]^+^) calcd: 521.2660; found: 521.2685.

##### (***R***,***S***)-3-(1-(1-(3-(5-(1-((1-Methylpiperidin-4-yl)oxy)ethyl)pyrimidin-2-yl)phenyl)ethyl)-6-oxo-1,6-dihydropyridazin-3-yl)benzonitrile ((***R***, ***S***)-**12a**)

Light yellow solid, yield: 45.4%. 1H NMR (400 MHz, Chloroform-d) δ 8.71 (s, 1H), 8.51 (s, 2H), 8.31 (d, *J* = 7.8 Hz, 1H), 8.24 (s, 1H), 8.00 (d, *J* = 8.5 Hz, 1H), 7.71 (d, *J* = 7.8 Hz, 1H), 7.63–7.55 (m, 3H), 7.45 (t, *J* = 7.7 Hz, 1H), 7.05 (d, *J* = 9.7 Hz, 1H), 6.57 (q, *J* = 6.9 Hz, 1H), 4.34 (q, *J* = 6.0 Hz, 1H), 3.01 (d, *J* = 9.7 Hz, 2H), 2.35 (s, 3H), 2.06–1.99 (m, 2H), 1.94 (d, *J* = 7.1 Hz, 3H), 1.76 (d, *J* = 9.6 Hz, 2H), 1.69–1.59 (m, 3H), 1.37 (d, *J* = 6.2 Hz, 3H); 13C NMR (126 MHz, CDCl3) δ 159.25, 157.51, 150.39, 145.25, 141.90, 140.64, 137.70, 136.32, 132.50, 130.26, 129.84, 129.78, 129.67, 128.80, 128.76, 127.28, 126.92, 118.74, 113.30, 56.21, 54.36, 54.22, 43.78, 38.99, 25.59, 24.66, 20.09, 16.28, and HR-ESI-MS for C_31_H_32_N_6_O_4_ ([M + H]^+^) calcd: 521.2660; found: 521.2685.

##### (***S***,***R***)-3-(1-(1-(3-(5-(1-((1-Methylpiperidin-4-yl)oxy)ethyl)pyrimidin-2-yl)phenyl)ethyl)-6-oxo-1,6-dihydropyridazin-3-yl)benzonitrile ((***S***, ***R***)-**12a**)

Light yellow solid, yield: 47.6%. ^1^H NMR (400 MHz, Chloroform-d) δ 8.70 (s, 1H), 8.51 (s, 2H), 8.30 (d, *J* = 7.8 Hz, 1H), 8.23 (s, 1H), 8.00 (d, *J* = 8.0 Hz, 1H), 7.71 (d, *J* = 7.7 Hz, 1H), 7.63–7.54 (m, 3H), 7.45 (t, *J* = 7.7 Hz, 1H), 7.05 (d, *J* = 9.7 Hz, 1H), 6.57 (q, *J* = 7.1 Hz, 1H), 4.35 (p, *J* = 6.1 Hz, 1H), 2.99 (d, *J* = 11.2 Hz, 2H), 2.34 (s, 3H), 2.04–1.97 (m, 2H), 1.94 (d, *J* = 7.1 Hz, 3H), 1.75 (d, *J* = 12.0 Hz, 2H), 1.67–1.50 (m, 3H), 1.36 (d, *J* = 6.2 Hz, 3H).; 13C NMR (126 MHz, CDCl3) δ 159.20, 157.51, 150.38, 145.23, 141.85, 140.63, 137.71, 136.34, 132.47, 130.26, 129.81, 129.76, 129.69, 128.77, 128.71, 127.27, 126.91, 118.72, 113.33, 56.18, 54.35, 54.21, 43.81, 39.05, 29.69, 24.71, 20.10, 16.27, and HR-ESI-MS for C_31_H_32_N_6_O_4_ ([M + H]^+^) calcd: 521.2660; found: 521.2679.

##### (***S***,***S***)-3-(1-(1-(3-(5-(1-((1-Methylpiperidin-4-yl)oxy)ethyl)pyrimidin-2-yl)phenyl)ethyl)-6-oxo-1,6-dihydropyridazin-3-yl)benzonitrile ((***S***, ***S***)-**12a**)

Light yellow solid, yield: 42.7%. 1H NMR (500 MHz) δ 8.69 (s, 1H), 8.51 (s, 2H), 8.28 (d, *J* = 7.7 Hz, 1H), 8.24 (s, 1H), 7.96 (d, *J* = 7.9 Hz, 1H), 7.69 (d, *J* = 7.6 Hz, 1H), 7.63–7.53 (m, 3H), 7.43 (t, *J* = 7.7 Hz, 1H), 7.03 (d, *J* = 9.7 Hz, 1H), 6.54 (q, *J* = 6.9 Hz, 1H), 4.43–4.38 (m, 1H), 3.44 (d, *J* = 10.0 Hz, 2H), 2.70 (s, 3H), 2.63 (t, *J* = 11.7 Hz, 2H), 2.11 (d, *J* = 14.1 Hz, 1H), 2.01 (m, 3H), 1.91 (d, *J* = 7.1 Hz, 3H), 1.88 –1.82(m,1H); ^13^C NMR (101 MHz, CDCl_3_) δ 159.23, 157.38, 150.51, 145.25, 141.85, 140.67, 137.71, 136.28, 132.44, 130.23, 129.84, 129.76, 129.71, 129.46, 128.77, 127.22, 126.87, 118.66, 113.26, 56.27, 54.55, 54.43, 44.21, 39.27, 26.01, 25.19, 20.11, 16.30, and HR-ESI-MS for C_31_H_32_N_6_O_4_ ([M + H]^+^) calcd: 521.2660; found: 521.2686.

##### (***R***,***R***)-3-(1-(1-(3-(5-(1-((1-Methyl-1,2,3,6-tetrahydropyridin-4-yl)oxy)ethyl)pyrimidin-2-yl)phenyl)ethyl)-6-oxo-1,6-dihydropyridazin-3-yl)benzonitrile ((***R***, ***R***)-**12b**)

Light yellow solid, yield: 58.2%. 1H NMR (400 MHz, Chloroform-d) δ 8.64 (s, 1H), 8.48 (s, 2H), 8.26 (d, *J* = 7.8 Hz, 1H), 8.21 (s, 1H), 7.97 (d, *J* = 8.0 Hz, 1H), 7.69 (d, *J* = 7.8 Hz, 1H), 7.62–7.53 (m, 3H), 7.44 (d, *J* = 7.7 Hz, 1H), 7.03 (d, *J* = 9.7 Hz, 1H), 6.53 (d, *J* = 7.1 Hz, 1H), 5.78 (s, 1H), 4.86 (q, *J* = 6.3 Hz, 1H), 3.24 (d, *J* = 15.8 Hz, 1H), 3.15 (d, *J* = 16.8 Hz, 1H), 2.90–2.85 (m, 1H), 2.72–2.66 (m, 1H), 2.51 (s, 3H), 2.43 (s, 1H), 2.28 (d, *J* = 17.4 Hz, 1H), 1.91 (d, *J* = 7.0 Hz, 3H), 1.54 (d, *J* = 6.4 Hz, 3H); ^13^C NMR (101 MHz, CDCl_3_) δ 159.24, 157.45, 150.49, 145.27, 141.80, 140.68, 137.72, 136.28, 132.41, 130.26, 129.81, 129.77, 129.76, 129.71, 129.50, 128.81, 128.70, 127.27, 126.88, 119.37, 118.64, 113.27, 77.77, 56.29, 52.31, 50.62, 43.75, 22.23, 20.02, 19.97, and HR-ESI-MS for C_31_H_30_N_6_O_4_ ([M + H]^+^) calcd: 521.2660; found: 521.2686.

##### (***R***,***S***)-3-(1-(1-(3-(5-(1-((1-Methyl-1,2,3,6-tetrahydropyridin-4-yl)oxy)ethyl)pyrimidin-2-yl)phenyl)ethyl)-6-oxo-1,6-dihydropyridazin-3-yl)benzonitrile ((***R***, ***S***)-**12b**)

Light yellow solid, yield: 60.2%. 1H NMR (500 MHz, Chloroform-d) δ 8.68 (s, 1H), 8.51 (s, 2H), 8.28 (d, *J* = 7.7 Hz, 1H), 8.25 (s, 1H), 7.95 (d, *J* = 7.9 Hz, 1H), 7.70 (d, *J* = 7.7 Hz, 1H), 7.62–7.54 (m, 3H), 7.03 (d, *J* = 9.7 Hz, 1H), 6.54 (t, *J* = 7.0 Hz, 1H), 5.80 (s, 1H), 4.90 (q, *J* = 6.1 Hz, 1H), 3.58 (d, *J* = 16.1 Hz, 1H), 3.49 (d, *J* = 16.3 Hz, 1H), 3.22 (dt, *J* = 11.9, 5.5 Hz, 1H), 3.04 (q, *J* = 10.3, 8.0 Hz, 1H), 2.74 (s, 3H), 2.69 (m, 1H), 2.48 (m, 1H), 1.92 (d, *J* = 7.0 Hz, 3H), 1.57 (d, *J* = 6.4 Hz, 3H); ^13^C NMR (126 MHz, CDCl_3_) δ 159.27, 157.42, 150.56, 145.28, 141.80, 140.67, 137.77, 136.34, 135.77, 132.42, 130.31, 129.84, 129.78, 129.72, 129.49, 128.82, 128.68, 127.27, 126.92, 120.42, 118.64, 113.31, 56.32, 52.80, 50.90, 44.30, 22.77, 19.98, and HR-ESI-MS for C_31_H_30_N_6_O_4_ ([M + H]^+^) calcd: 519.2503; found: 519.2540.

##### (***S***,***R***)-3-(1-(1-(3-(5-(1-((1-Methyl-1,2,3,6-tetrahydropyridin-4-yl)oxy)ethyl)pyrimidin-2-yl)phenyl)ethyl)-6-oxo-1,6-dihydropyridazin-3-yl)benzonitrile ((***S***, ***R***)-12b)

Light yellow solid, yield: 56.3%. 1H NMR (500 MHz, Chloroform-d) δ 8.66 (s, 1H), 8.50 (s, 2H), 8.28 (d, *J* = 7.7 Hz, 1H), 8.23 (s, 1H), 7.98 (d, *J* = 8.0 Hz, 1H), 7.70 (d, *J* = 7.7 Hz, 1H), 7.63–7.54 (m, 3H), 7.44 (t, *J* = 7.7 Hz, 1H), 7.04 (d, *J* = 9.7 Hz, 1H), 6.55 (q, *J* = 7.0 Hz, 1H), 5.79 (s, 1H), 4.88 (q, *J* = 6.2 Hz, 1H), 3.29 (d, *J* = 16.6 Hz, 1H), 3.21 (d, *J* = 16.5 Hz, 1H), 2.92 (m, 1H), 2.74 (m, 1H), 2.54 (s, 3H), 2.48 (s, 1H), 2.32 (d, *J* = 17.3 Hz, 1H), 1.92 (d, *J* = 7.0 Hz, 3H), 1.55 (d, *J* = 6.4 Hz, 3H); ^13^C NMR (126 MHz, CDCl_3_) δ 159.24, 157.66, 150.43, 145.32, 141.82, 140.68, 137.69, 136.40, 136.32, 132.42, 130.29, 129.81, 129.78, 129.70, 128.84, 128.69, 127.34, 126.88, 118.69, 117.84, 113.29, 77.32, 56.23, 51.71, 50.31, 43.07, 21.48, 19.97, and HR-ESI-MS for C_31_H_30_N_6_O_4_ ([M + H]^+^) calcd: 519.2503; found: 519.2521.

##### (***S***,***S***)-3-(1-(1-(3-(5-(1-((1-Methyl-1,2,3,6-tetrahydropyridin-4-yl)oxy)ethyl)pyrimidin-2-yl)phenyl)ethyl)-6-oxo-1,6-dihydropyridazin-3-yl)benzonitrile (**(*S, S*)-12b**)

Light yellow solid, yield: 59.7%. 1H NMR (400 MHz, Chloroform-d) δ 8.70 (s, 1H), 8.50 (s, 2H), 8.28 (d, *J* = 7.8 Hz, 1H), 8.25 (s, 1H), 7.95 (d, *J* = 7.9 Hz, 1H), 7.69 (d, *J* = 7.7 Hz, 1H), 7.61–7.53 (m, 3H), 7.43 (t, *J* = 7.7 Hz, 1H), 7.02 (d, *J* = 9.7 Hz, 1H), 6.54 (d, *J* = 7.0 Hz, 1H), 5.79 (s, 1H), 4.88 (p, *J* = 7.7, 7.0 Hz, 1H), 3.34 (d, *J* = 17.1 Hz, 1H), 3.26 (d, *J* = 16.3 Hz, 1H), 2.99–2.93 (m, 1H), 2.78 (dt, *J* = 11.3, 6.4 Hz, 1H), 2.57 (s, 3H), 2.51 (d, *J* = 10.3 Hz, 1H), 2.33 (d, *J* = 17.7 Hz, 1H), 1.92 (d, *J* = 7.0 Hz, 3H), 1.54 (d, *J* = 6.4 Hz, 3H); ^13^C NMR (101 MHz, CDCl_3_) δ 158.22, 156.42, 149.47, 144.25, 140.78, 139.65, 136.70, 135.26, 134.92, 131.38, 129.24, 128.78, 128.74, 128.69, 128.47, 127.78, 127.67, 126.25, 125.86, 118.45, 117.61, 112.25, 55.28, 51.35, 49.63, 42.79, 21.26, 19.00, 18.95, and HR-ESI-MS for C_31_H_30_N_6_O_4_ ([M + H]^+^) calcd: 519.2503; found: 519.2544.

### 4.2. Biological Effects

#### 4.2.1. Cell Culture and Cytotoxicity Assay

MHCC97H were purchased from Kangyuan Bochuang Biotechnology co. LTD (Beijing, China) and cultured with DMEM medium containing 10% FBS. The in vitro antiproliferative activity of each compound was assayed using MTT method. In brief, cells were seeded in 96-well plates, at the density of 5 × 10^3^/well. After medium removal, 100 μL of culture media with 0.1% DMSO containing each compound at different concentrations was added to each well, and incubation continued for another 48 h at 37 °C. DMSO (0.1%) and tepotinib were used as the negative and positive controls, respectively. MTT solution (5 mg/mL in PBS) was added, and incubation continued for another 4 h. The optical density was detected with a microplate reader at 570 nm (BioTek, Winooski, VT, USA). The IC_50_ value of each compound was calculated using GraphPad Prism 8. All the experiments were repeated independently at least three times.

#### 4.2.2. Kinase Inhibition Assay

The kinase inhibition assay was carried out according to the principle of MSA (Mobility Shifting Assay) detection. Briefly, MET (08-151, Carna Bioscience, Kobe, Japan) was prepared for 1-fold kinase buffer, and each compound was dissolved in DMSO as a stock solution of 10 mM. Using triple dilution method, 10 gradient concentration groups were received. Varying concentrations of compounds were transported in 384-well plates, and 100% DMSO was set as negative control. After adding kinase buffer, the 384-well plates were centrifuged and vortexed, followed by 10 min of incubation. Then, ATP and kinase substrate 2 (190861, GL Science, Tokyo, Japan), diluted in kinase buffer, were shifted in the plate and incubated for 60 min after thorough mixing. Finally, adding stop solution ended kinase reaction, and the conversion% was detected by a microplate reader (Caliper EZ Reader II, Perkin Elmer, Waltham, Massachusetts, Germany). The statistics were analyzed by GraphPad Prism 8, using log (inhibitor) vs. response–variable slope to fit the dose–effect curve.

#### 4.2.3. Cell Cycle Analysis

MHCC97H cells were incubated with varying concentrations of compound **(*R*, *S*)-12a** or 1‰ DMSO for 48 h. The cells were collected by centrifugation, washed with PBS, and fixed in 70% ice-cold ethanol at 4 °C for 12 h. After the removal of ethanol, each sample was re-suspended in 500 μL of staining buffer containing 50 μL of RNase A and 450 μL of propidium iodide (KGA511, KeyGEN, BioTECH, Nanjing, Jiangsu, China) and incubated at room temperature, avoiding light exposure for 30 min. The DNA content of the cells was measured using a CytoFLXE flow cytometer (BECKMAN COLTER, Brea, CA, USA).

#### 4.2.4. Cellular Apoptosis Analysis [31]

MHCC97H cells were incubated with varying concentrations of compound **(*R*, *S*)-12a** or diluent (1‰ DMSO, as a negative control) for 48 h. The cells were harvested and washed with PBS twice. Then, 500 μL of binding buffer suspended cells were added. The cells were stained with 5 μL of Annexin V-FITC and 10 μL of PI (KGA105, KeyGEN, BioTECH, Nanjing, Jiangsu, China). Then, the cells were incubated at room temperature for 15 min without light exposure. Apoptosis was analyzed using a CytoFLXE flow cytometer (BECKMAN COLTER, Brea, CA, USA).

#### 4.2.5. Wound Healing Assay [34]

MHCC97H cells were seeded in a 6-well plate and cultured for 24 h; subsequently, the confluent monolayer cells were scratched with a 10 μL pipette tip. After that, the wound was washed with PBS thrice to remove the non-adherent cell debris. In addition, each petri dish had media containing **(*R*, *S*)-12a** in gradient concentrations (1, 2, and 3 nM) added. At 0, 6, 12, and 24 h, changes of cell scratch in area were photographed by phase contrast microscope and finally measured and analyzed by Image J.

#### 4.2.6. Immunofluorescence Staining

MHCC97H cells were seeded into confocal dishes and then treated with vehicle control (1‰ DMSO) and compound **(*R*, *S*)-12a** (2.5 nM). Cells were washed thrice with PBS, fixed with 4% paraformaldehyde for 15 min, and punched with 0.2% triton X100 and then blocked with 500 μL of blocking buffer at room temperature for 1 h. Cells were incubated with a primary monoclonal antibody (c-Met, p-c-Met, AKT, and p-AKT) at 4 °C for 12 h and then washed with PBS thrice, followed by incubation with the fluorescence secondary antibody (Alexa Fluor 488-labeled Goat Anti-Mouse IgG (H + L)-A0428) and labeling of nuclei by Hoechst33342 (MA0126, Meilunbio, Dalian, Liaoning, China). The cells were finally visualized by a confocal microscope (Carl Zessi Microscopy LSM 880, Oberkochen, Germany). The average fluorescence intensity of FITC channel was analyzed by Image J, and the illustration was drafted by GraphPad Prism8.

#### 4.2.7. Western Blotting Analysis [35,36,37]

MHCC97H cells were then homogenized with lysis buffer. The protein concentrations were detected using a BCA Protein Assay Kit (Pierce BCA Protein Assay Kit 23227, Thermo, Waltham, MA, USA), and their extracts were reconstituted within loading buffer (Beyotime, Shanghai, China) and inactivated for 5 min at 100 °C. Subsequently, proteins (20 μg) were fractionated by 10% SDS-PAGE and transferred to PVDF membranes. Then, PVDF membranes were blocked in 10% skimmed milk and incubated with the appropriate dilution of primary antibodies, including c-Met (Abcam, Cambridge, UK), p-c-Met (Abcam), AKT (Cell Signaling Technology), p-AKT (Cell Signaling Technology), and GAPDH (Cell Signaling Technology, Denver, MA, USA). The above primary antibodies were then reacted with HRP-conjugated secondary antibody, and immunoreactive bands were visualized by an ECL Detection Reagent (36208ES60, Yeasen, Shanghai, China) and were estimated through densitometry with a chemiluminescence imaging system (Tanon, Shanghai, China). Intensities of the blots were quantified with Image J and analyzed by GraphPad Prism 8.

#### 4.2.8. Molecular Docking

All docking studies were performed by Schrödinger suites (Maestro v.9.0, Schrödinger Inc., New York, NY, USA). For ligand preparation, 3D structures of all ligands were constructed using ChemBio3D ultra14.0, and then the initial lowest energy conformations were calculated with igPrep. For protein preparation, the X-ray crystal structures of c-Met (4R1V) [22] were downloaded from the RCSB PDB Bank (http://www.rcsb.org/ (accessed on 22 May 2022)), and then Protein Preparation Wizard was used with default settings to pre-process and refine. For dockings, the grid center was placed at the centroid of the ligand-binding site, and then Glide with SP protocol was used to perform the docking study. The docking poses were affirmed by PyMOL [45].

#### 4.2.9. Molecular Dynamics Simulations

The stability of docked ligand–c-Met complexes and conformation changes was analyzed by molecular dynamics simulation using Schrödinger suites (Desmond v3.8, Schrödinger Inc., NY, USA). The systems applied the OPLS_2005 force field and simple point charge (SPC) water model [46]. Each complex was built with the use of the highest-scoring docking pose. All the 10 ns simulations were run on our servers.

## Figures and Tables

**Table 1:** In vitro c-Met kinase inhibition and antiproliferative activity against MHCC97H of compounds **12a-b**.

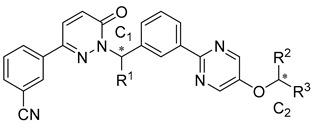							
Comp.	Config. of C1	Config. of C2	R1	R2	R3	Kinase	MHCC97H Cell
						IC50 (μM) ^a^	
(*R*, *R*)-12a	R	R	-CH_3_	-CH_3_	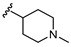	0.0287 ± 0.005	0.030 ± 0.008
(*R, S*)-12a	R	S	-CH_3_	-CH_3_		0.00156 ± 0.001	0.002 ± 0.002
(*S*, *R*)-12a	S	R	-CH_3_	-CH_3_		0.195 ± 0.0732	0.21 ± 0.095
(*S*, *S*)-12a	S	S	-CH_3_	-CH_3_		0.0358 ± 0.0068	0.048 ± 0.018
(*R*, *R*)-12b	R	R	-CH_3_	-CH_3_	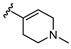	/	0.13 ± 0.038
(*R, S*)-12b	R	S	-CH_3_	-CH_3_		/	0.009 ± 0.005
(*S*, *R*)-12b	S	R	-CH_3_	-CH_3_		/	0.616 ± 0.0782
(*S*, *S*)-12b	S	S	-CH_3_	-CH_3_		/	0.136 ± 0.052
(±)-5b	/	/	-CH_3_	-H	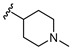	/	0.0353 ± 0.011
(*R*)-5b	R	/	-CH_3_	-H		/	0.0037 ± 0.001
(*S*)-5b	S	/	-CH_3_	-H		/	0.1182 ± 0.056
Tepotinib	/	/			/	0.0033 ± 0.0012	0.013 ± 0.002

^a^ Each IC_50_ value was indicated as the mean ± SD of three independent experiments. The IC_50_ values of (±)-5b, (R)-5b, and (S)-5b were referred from our previous work.

**Table 2:** IC_50_ of compound ***(R, S)*-12a** and tepotinib on MHCC-97H, Hacat, and LO2.

Compound	MHCC-97H	HaCat	Selectivity Index	LO2	Selectivity Index
Tepotinib	0.013 ± 0.002 μM	1.951 ± 0.349 μM	150.08	8.73 ± 0.841 μM	671.54
(*R, S*)*-*12a	0.002 ± 0.002 μM	1.907 ± 0.25 μM	953.5	11.50 ± 1.53 μM	5750.0

## Data Availability

The data presented in this study are available in this article.

## Supplementary Materials

The following supporting information can be downloaded at: https://www.mdpi.com/article/10.3390/molecules27175359/s1: NMR, HPLC, and HPLC of chiral purity and HRMS spectra data of compounds **12a-b**; X-ray diffraction data of **R-5b**.

## Sample Availability

Samples of the compounds are available from the corresponding authors.
