# Supplementary material for: Design, Synthesis, and Evaluation of New Mesenchymal–Epithelial Transition Factor (c-Met) Kinase Inhibitors with Dual Chiral Centers
Source: Molecules. 2022 Aug 23;27(17):5359. doi: 10.3390/molecules27175359 (PMC9457593; doi:10.3390/molecules27175359)
Supplement: Supplementary file 1 [file molecules-27-05359-s001.zip › molecules-1853129-supplementary.pdf]

# Supplementary Material


| Contents | Pages |
|---|---|
| NMR Spectra | **1-9** |
| HPLC Spectra | **10-13** |
| HPLC Spectra of Chiral Purity | **13-19** |
| HRMS Spectra | **20-27** |
| X-ray diffraction data | **28-42** |

**NMR Spectra**

**Compound (*R*, *R*)-12a**

**H[1] NMR**

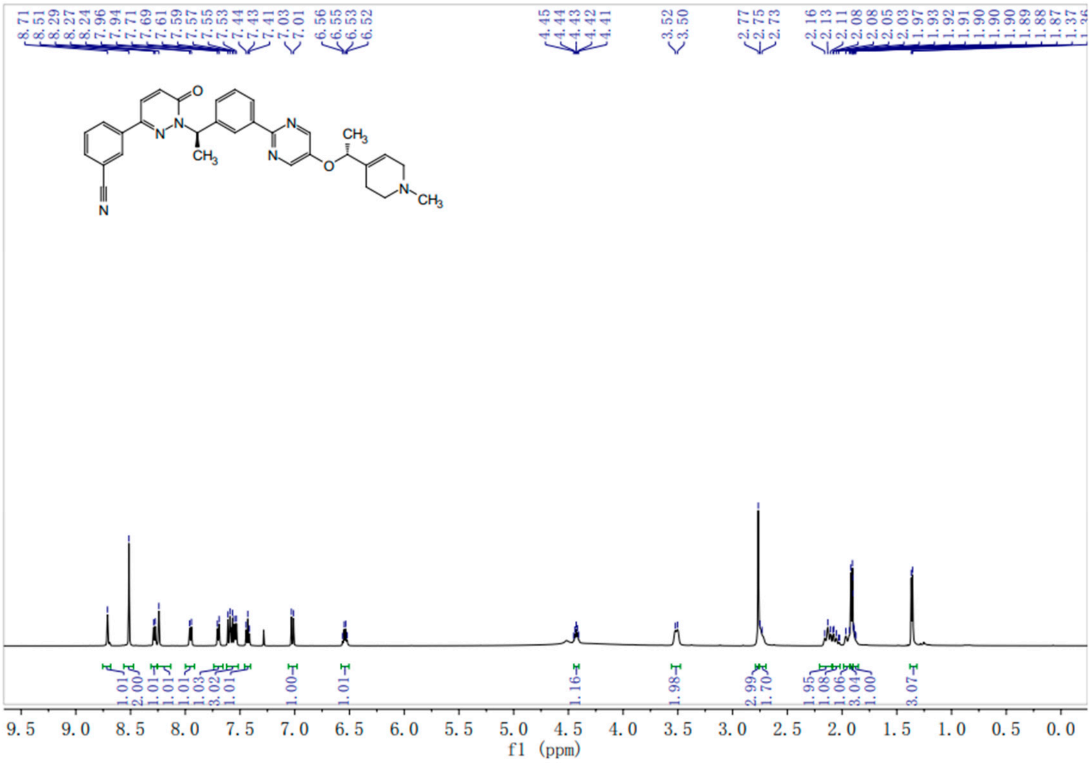

**C[13] NMR**

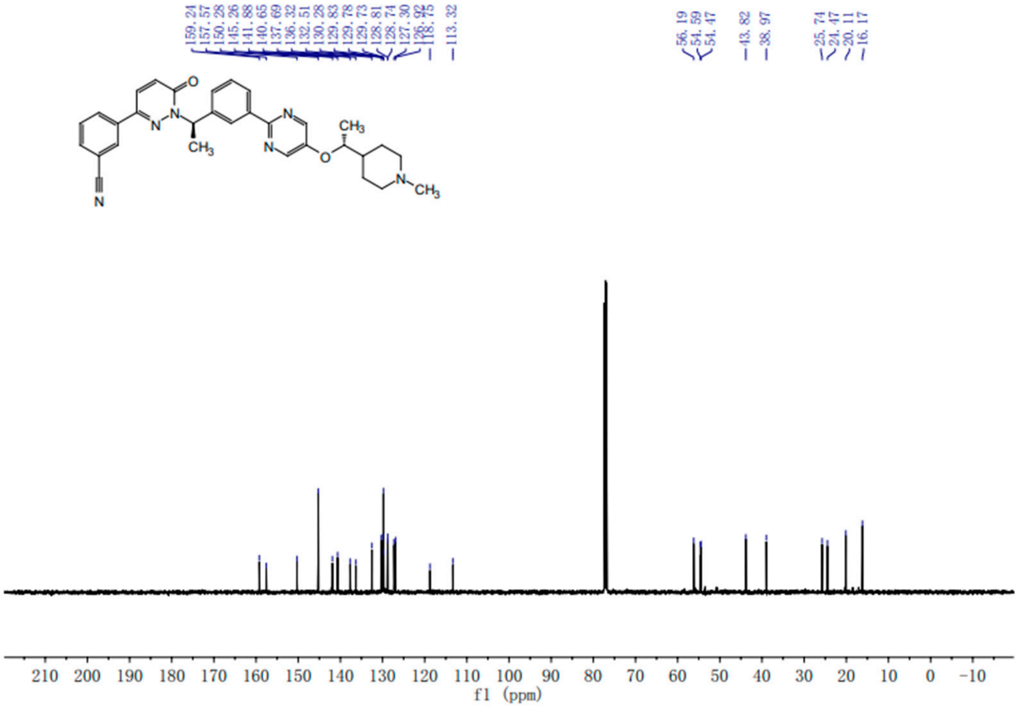

**Compound (*R*, *S*)-12a**
**H[1] NMR**

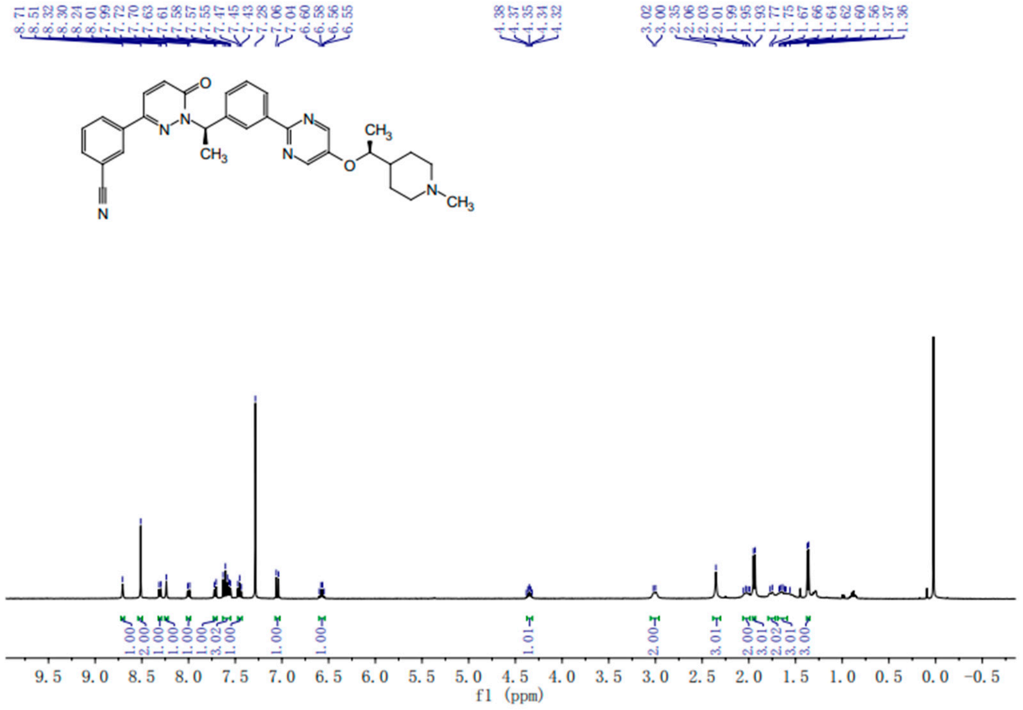


**C[13] NMR**

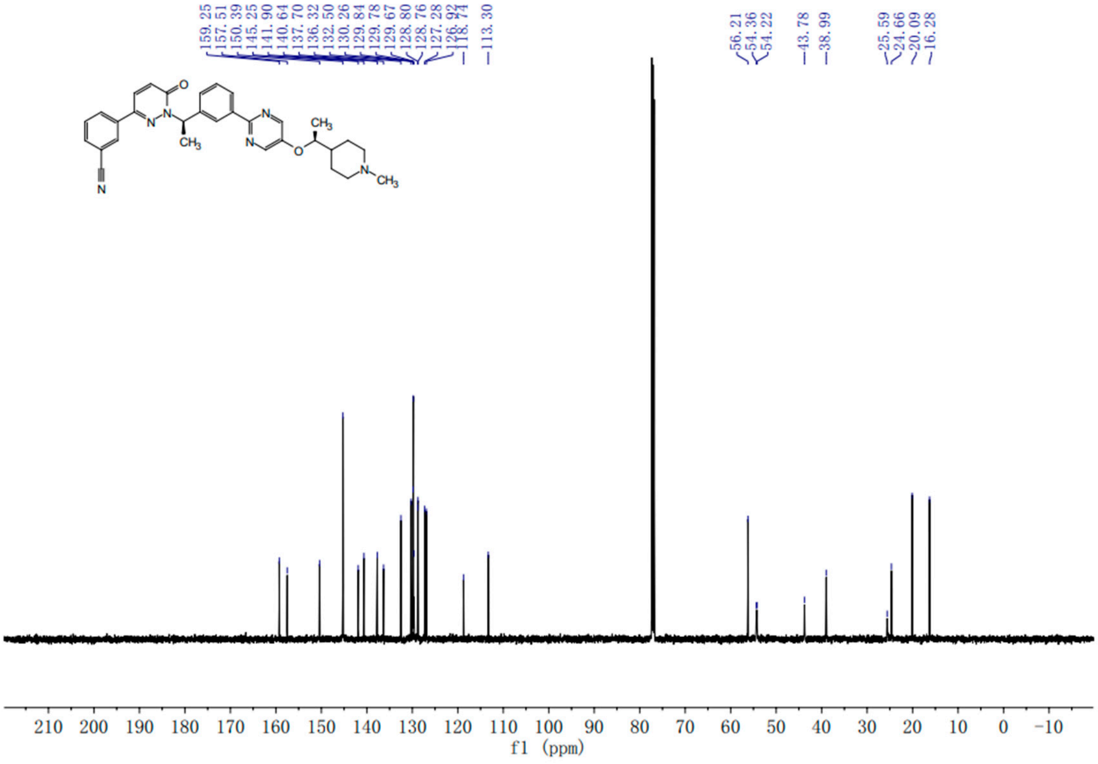

**Compound (*S, R*)-12a**

**H[1] NMR**

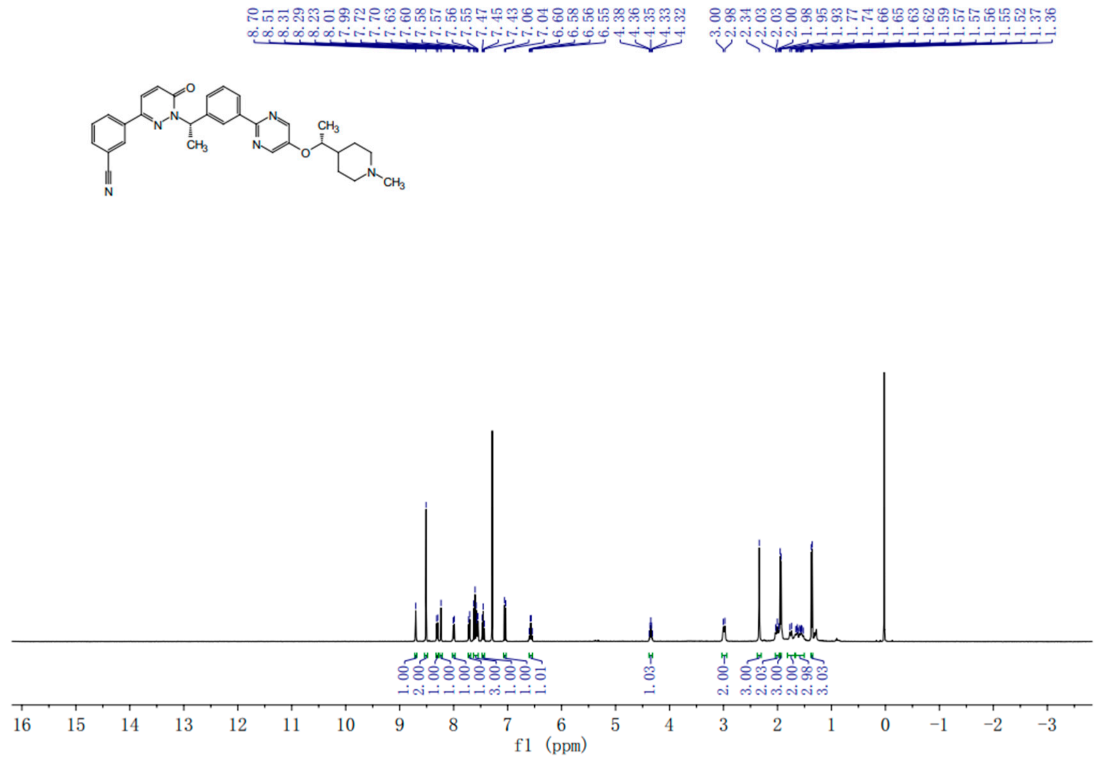


**C[13] NMR**

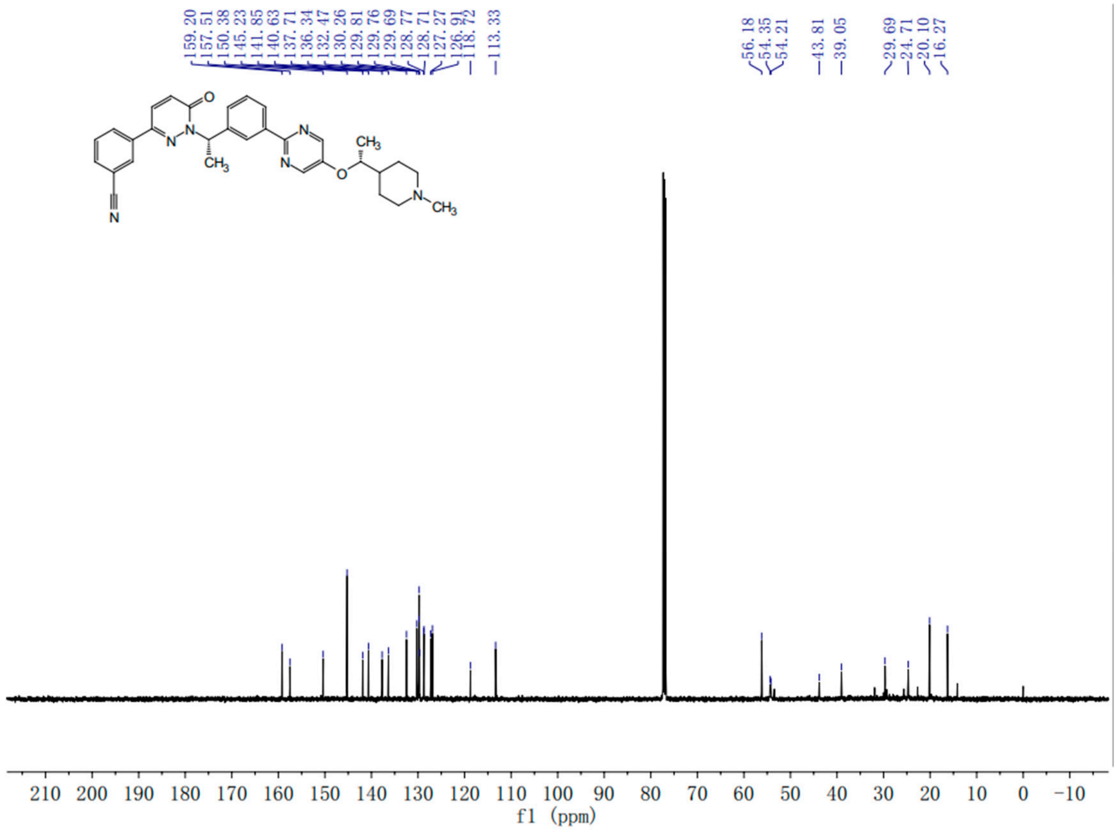

**Compound (*S*, *S*)-12a**

**H[1] NMR**

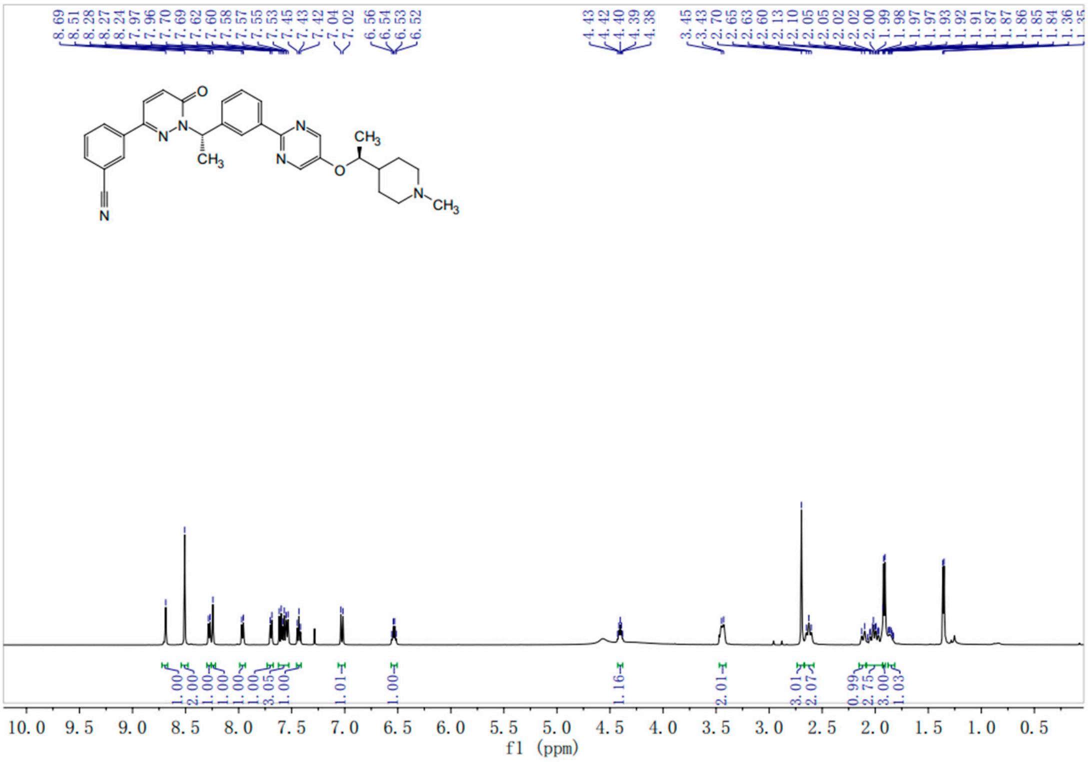

**C[13] NMR**

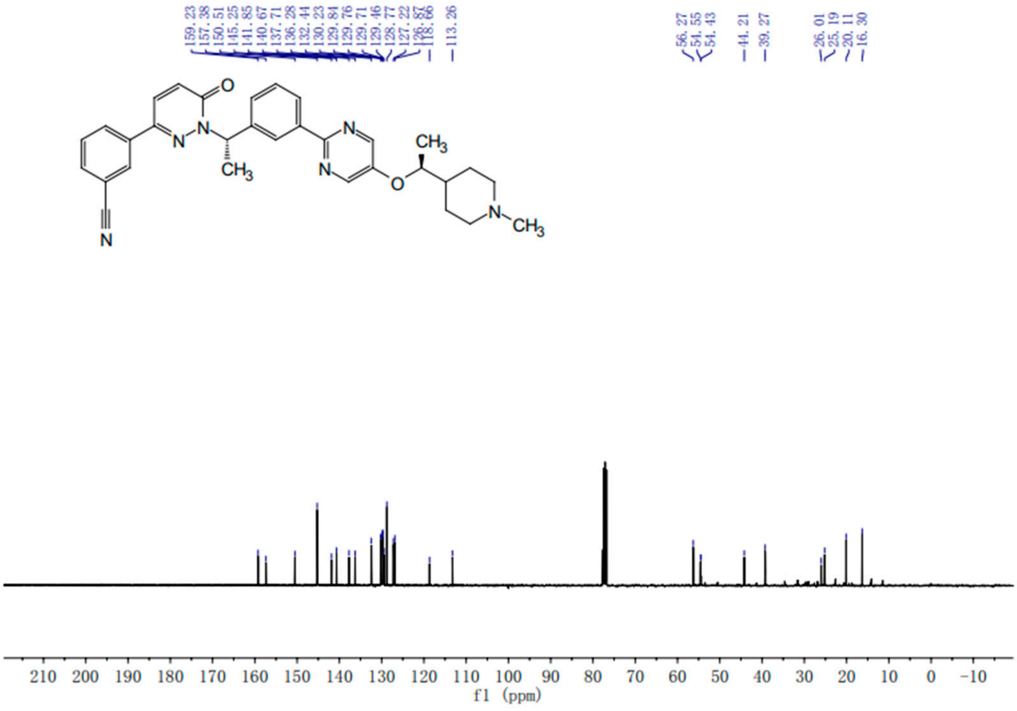

**Compound (*R*, *R*)-12b**

**H[1] NMR**

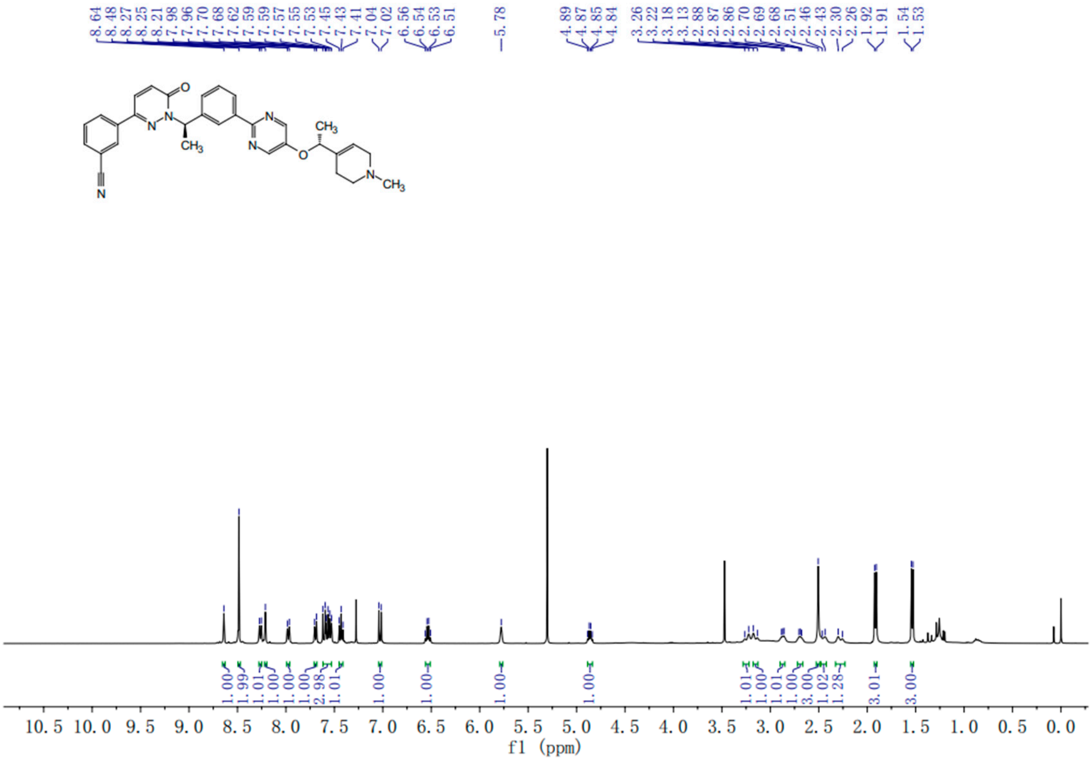


**C[13] NMR**

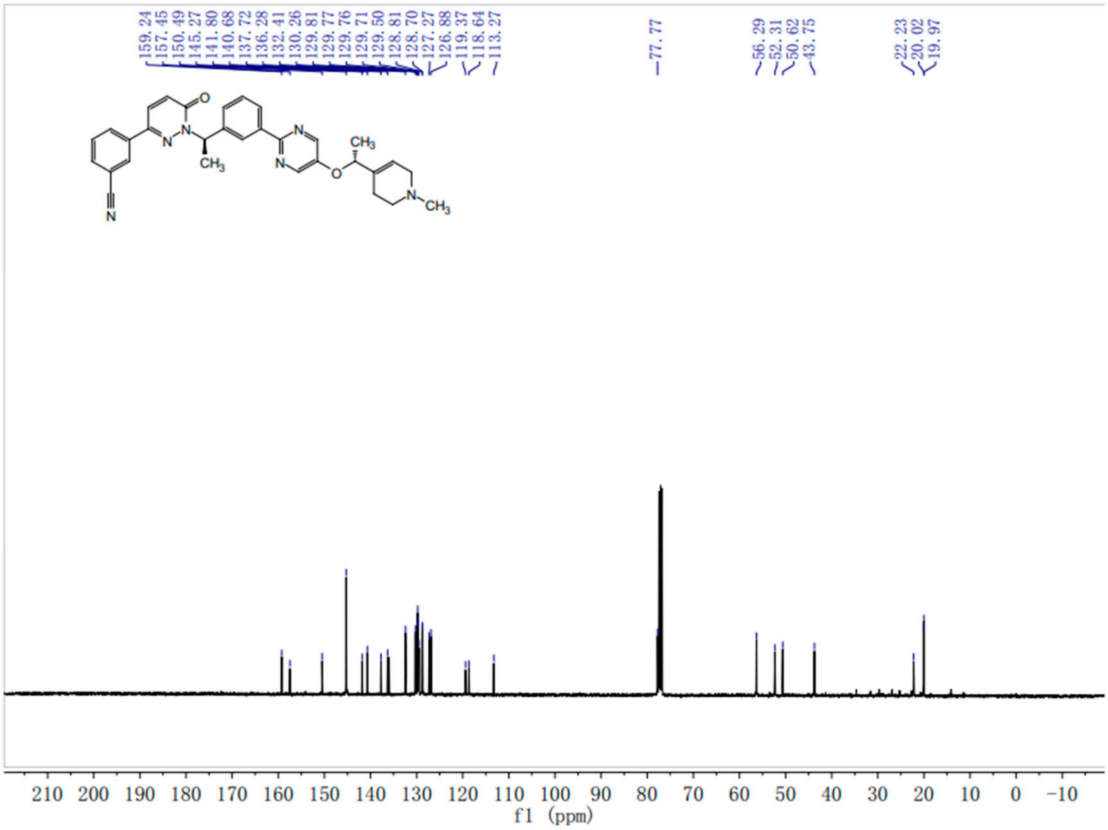

**Compound (*R*, *S*)-12b**

**H[1] NMR**

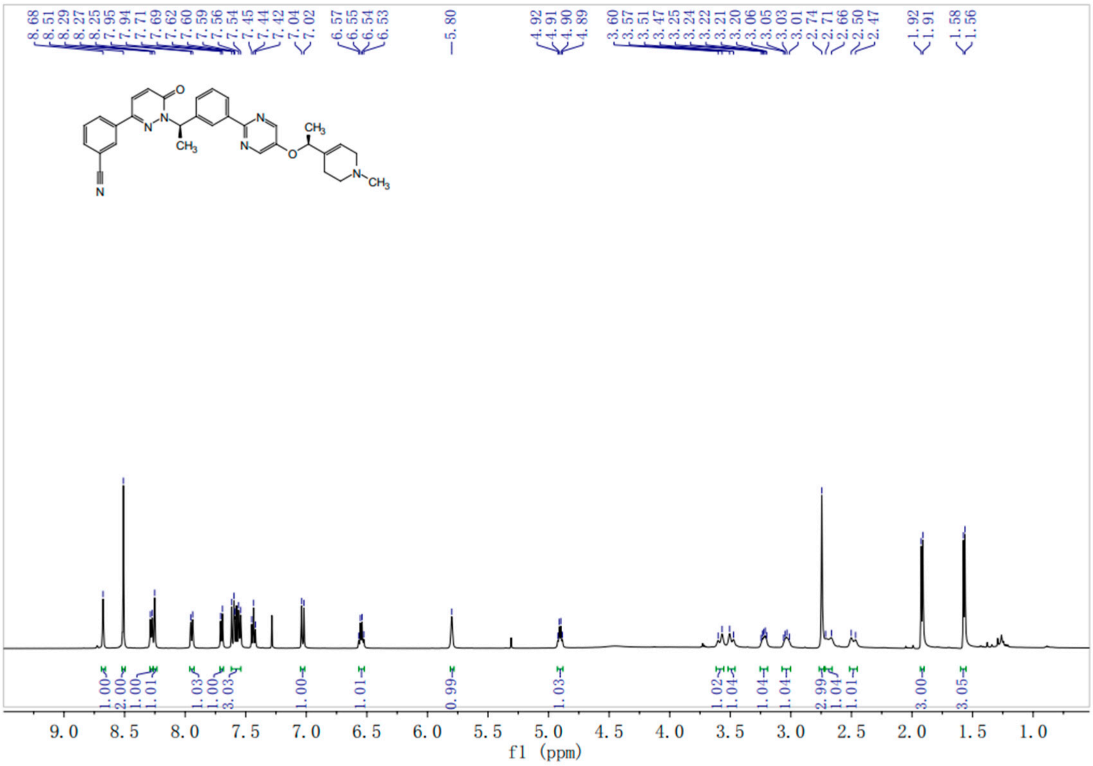


**C[13] NMR**

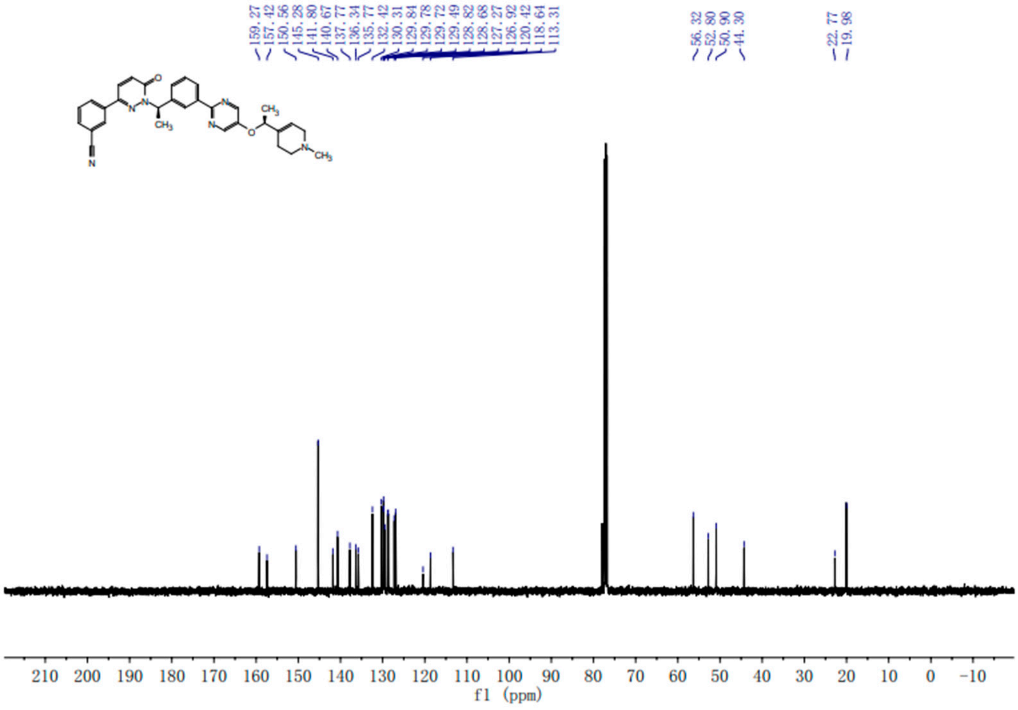

**Compound (*S, R*)-12b**

**H[1] NMR**

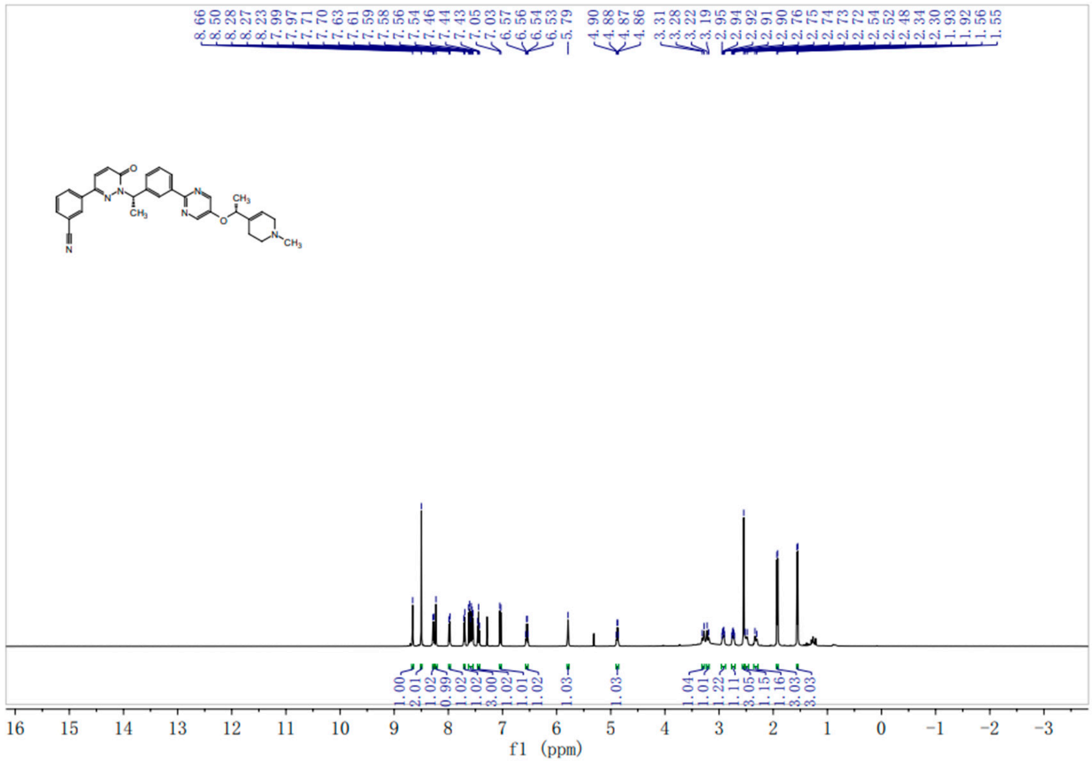


**C[13] NMR**

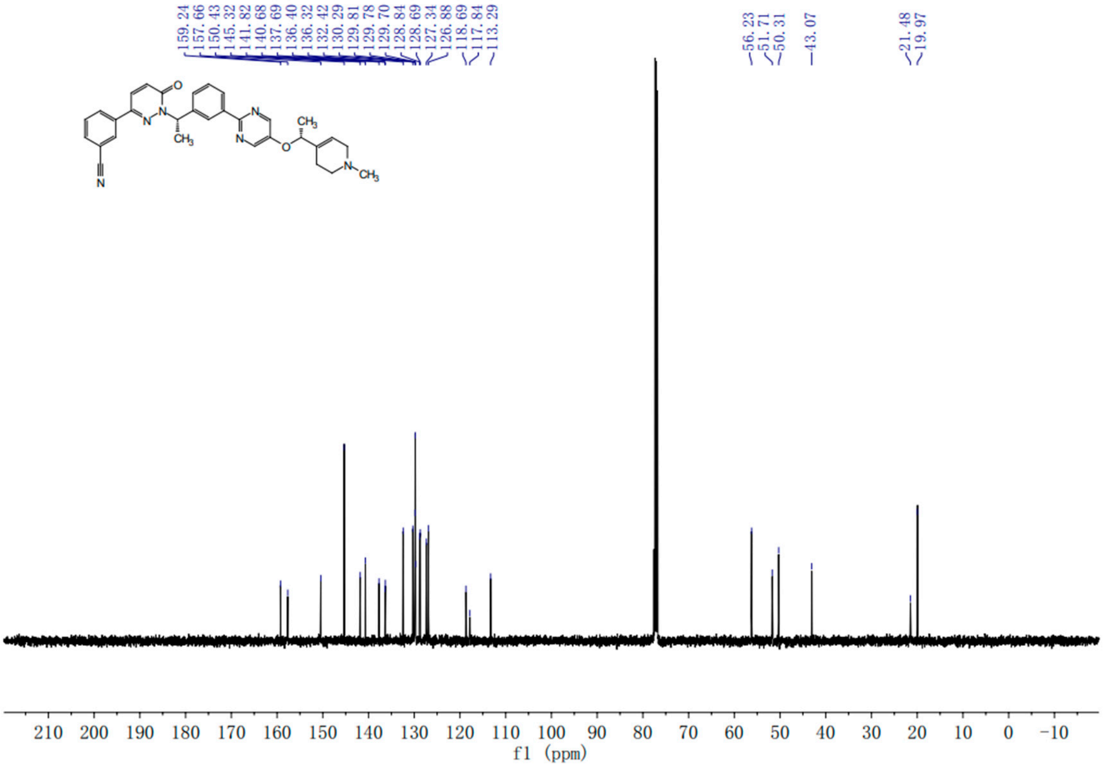

**Compound (*S, S*)-12b**

**$H^1$ NMR**

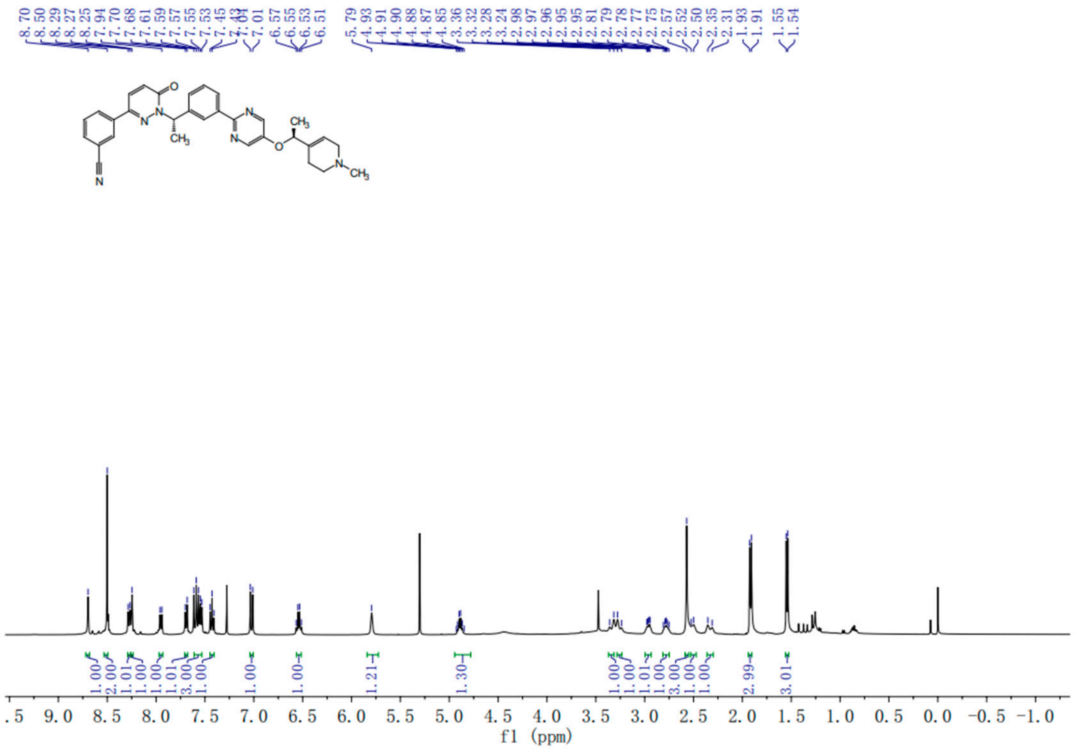

**$C^{13}$ NMR**

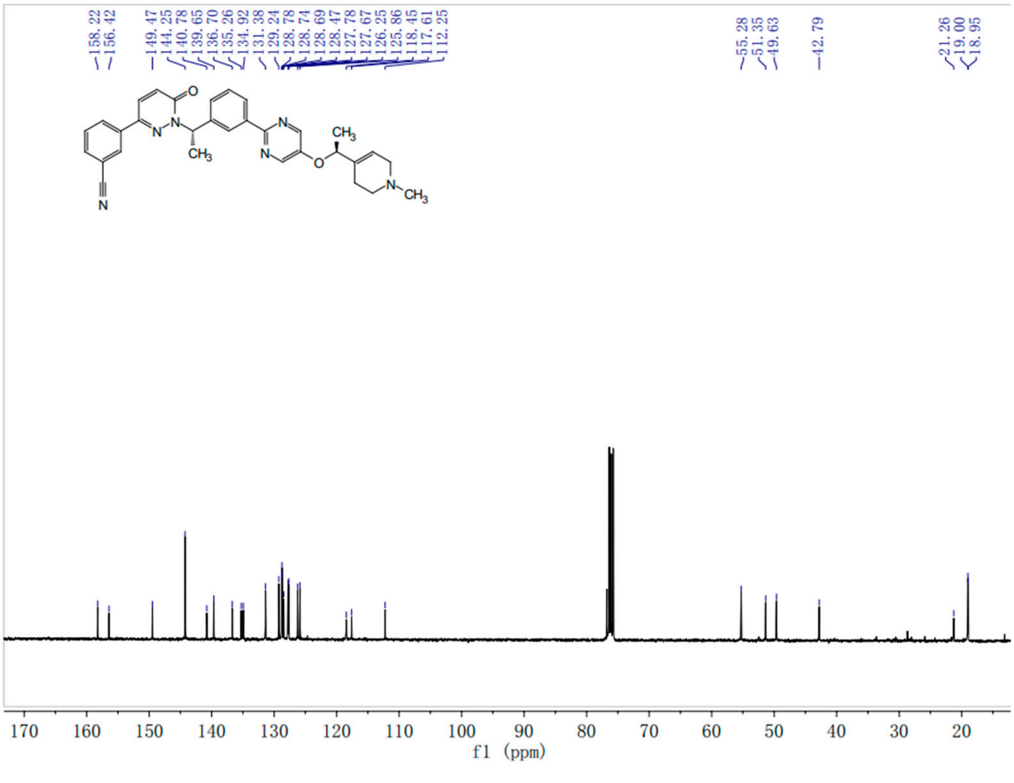

## HPLC spectra of compounds

## Compound (*R*, *R*)-12a

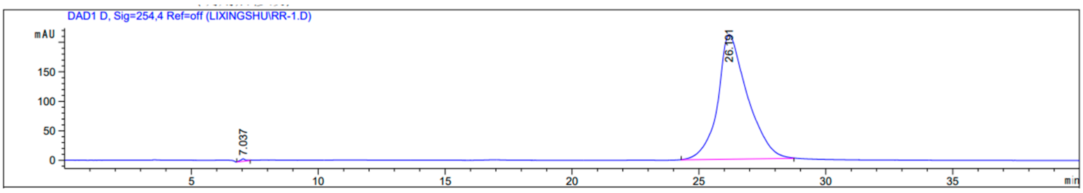


Signal 1: DAD1 D, Sig=254,4 Ref=off

| Peak # | Ret.Time [min] | Type | width [min] | Area [mAU*s] | Height [mAU] | Area % |
|---|---|---|---|---|---|---|
| 1 | 7.037 | BB | 0.1861 | 59.63461 | 4.24042 | 0.3464 |
| 2 | 26.191 | BB | 1.1411 | 1.71540e4 | 211.31198 | 99.6536 |
| Total : | | | | 1.72136e4 | 215.55240 | |

## Compound (*R*, *S*)-12a

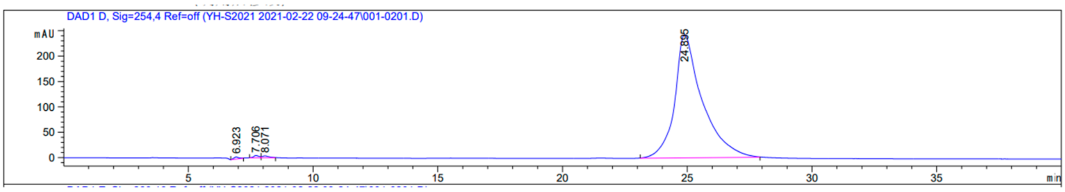


Signal 1: DAD1 D, Sig=254,4 Ref=off

| Peak # | Ret.Time [min] | Type | width [min] | Area [mAU*s] | Height [mAU] | Area % |
|---|---|---|---|---|---|---|
| 1 | 6.923 | BB | 0.1994 | 64.00987 | 4.30612 | 0.3380 |
| 2 | 7.706 | BV | 0.2265 | 70.79807 | 4.52691 | 0.3739 |
| 3 | 8.071 | VB | 0.2785 | 73.02588 | 3.72826 | 0.3856 |
| 4 | 24.895 | BB | 1.0696 | 1.87291e4 | 242.89191 | 98.9025 |
| Total : | | | | 1.89369e4 | 255.45320 | |

## Compound (*S, R*)-12a

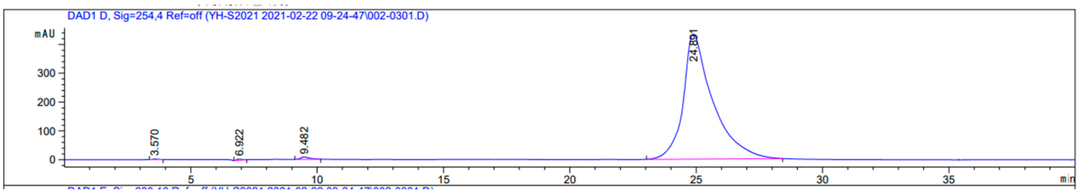


Signal 1: DAD1 D, Sig=254,4 Ref=off

| Peak # | Ret.Time [min] | Type | width [min] | Area [mAU*s] | Height [mAU] | Area % |
|---|---|---|---|---|---|---|
| 1 | 3.570 | BB | 0.2051 | 27.78988 | 1.87248 | 0.0778 |
| 2 | 6.922 | BB | 0.2073 | 65.20597 | 4.24351 | 0.1826 |
| 3 | 9.482 | BB | 0.3592 | 204.64351 | 8.03437 | 0.5729 |
| 4 | 24.891 | BB | 1.1350 | 3.54210e4 | 433.63293 | 99.1667 |

Total : 3.57187e4 447.78330

## Compound (*S, S*)-12a

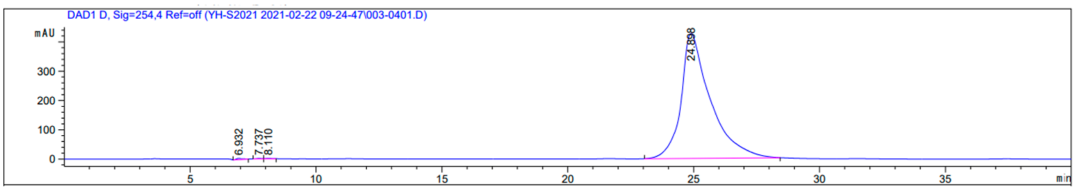


Signal 1: DAD1 D, Sig=254,4 Ref=off

| Peak # | Ret.Time [min] | Type | width [min] | Area [mAU*s] | Height [mAU] | Area % |
|---|---|---|---|---|---|---|
| 1 | 7.037 | BB | 0.1861 | 59.63461 | 4.24042 | 0.3464 |
| 2 | 26.191 | BB | 1.1411 | 1.71540e4 | 211.31198 | 99.6536 |

Total : 1.72136e4 215.55240

**Compound (*R*, *R*)-12b**

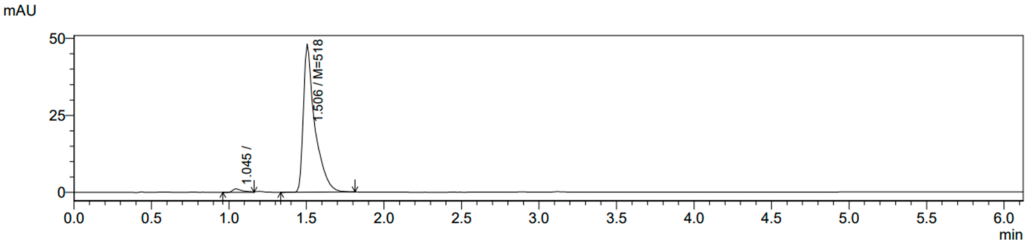


| Ret.Time | Compounds | Width(Height 50%) | Height | Area | Area% | N(USP) | T | R(USP) |
|---|---|---|---|---|---|---|---|---|
| 1.045 | | 0.059 | 1140 | 5031 | 2.004 | 1304 | -- | -- |
| 1.506 | M=518 | 0.069 | 48161 | 246057 | 97.996 | 1849 | 1.886 | 3.602 |
| | | | 49301 | 251088 | 100.000 | | | |

**Compound (*R*, *S*)-12b**

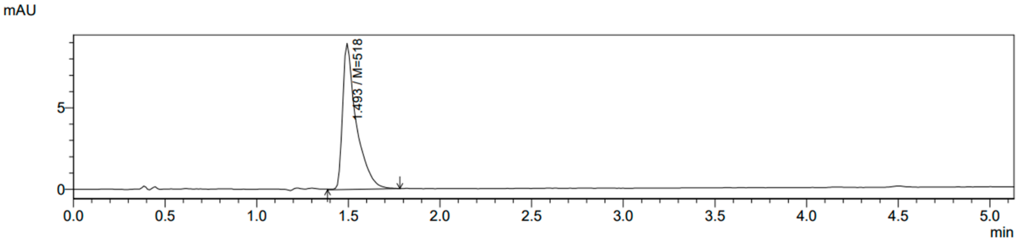


| Ret.Time | Compounds | Width(Height 50%) | Height | Area | Area% | N(USP) | T | R(USP) |
|---|---|---|---|---|---|---|---|---|
| 1.493 | M=518 | 0.070 | 8975 | 46527 | 100.000 | 1772 | 1.977 | -- |
| | | | 8975 | 46527 | 100.000 | | | |

## Compound (*S, R*)-12b

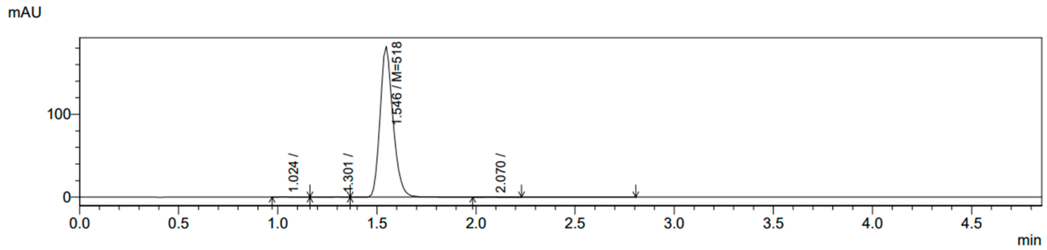


| Ret.Time | Compounds | Width(Height 50%) | Height | Area | Area% | N(USP) | T | R(USP) |
|---|---|---|---|---|---|---|---|---|
| 1.024 | | 0.074 | 356 | 1801 | 0.217 | 816 | 1.822 | -- |
| 1.301 | | 0.072 | 271 | 1510 | 0.182 | 1122 | -- | 1.856 |
| 1.546 | M=518 | 0.068 | 182115 | 823796 | 99.435 | 2064 | 1.218 | 1.675 |
| 2.070 | | 0.087 | 237 | 1368 | 0.165 | 2443 | 1.470 | 3.452 |
| | | | 182979 | 828474 | 100.000 | | | |

## Compound (*S, S*)-12b

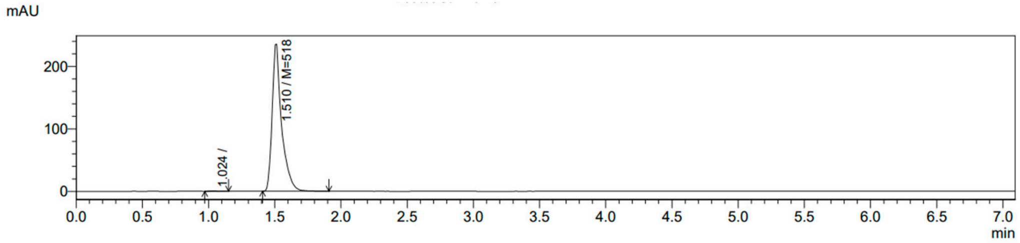


| Ret.Time | Compounds | Width(Height 50%) | Height | Area | Area% | N(USP) | T | R(USP) |
|---|---|---|---|---|---|---|---|---|
| 1.024 | | 0.055 | 796 | 3168 | 0.270 | 1451 | 1.774 | -- |
| 1.510 | M=518 | 0.068 | 235375 | 1168377 | 99.730 | 2185 | 1.509 | 4.100 |
| | | | 236171 | 1171545 | 100.000 | | | |

## HPLC Spectra of Chiral Purity

## Mixture of enantiomer (*R, R*)-12a and (*S, S*)-12a

**<Chromatogram>**

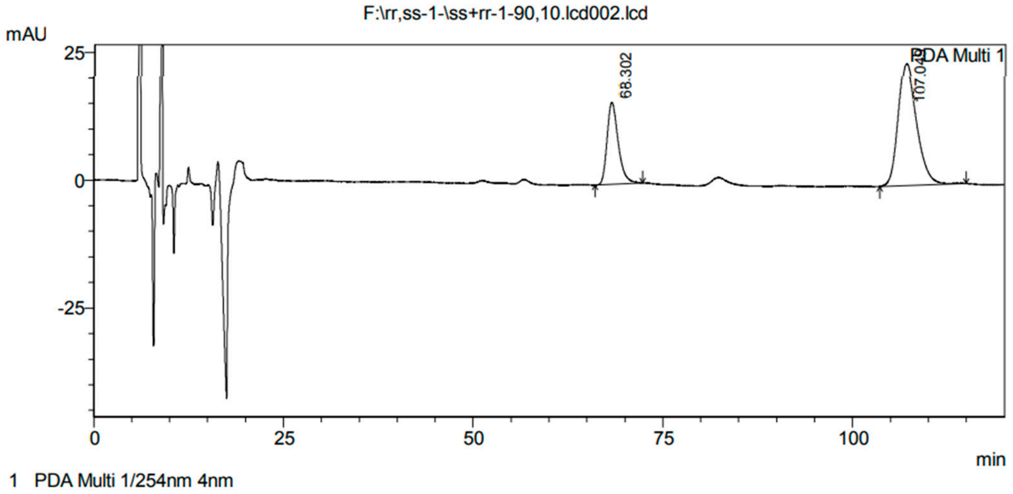


PeakTable

PDA Ch1 254nm 4nm

| Peak# | Ret. Time | Area | Height | Area % | Height % |
|---|---|---|---|---|---|
| 1 | 68.302 | 1629872 | 16034 | 28.806 | 40.228 |
| 2 | 107.049 | 4028229 | 23824 | 71.194 | 59.772 |
| Total | | 5658101 | 39858 | 100.000 | 100.000 |

## Compound (*R, R*)-12a

**<Chromatogram>**

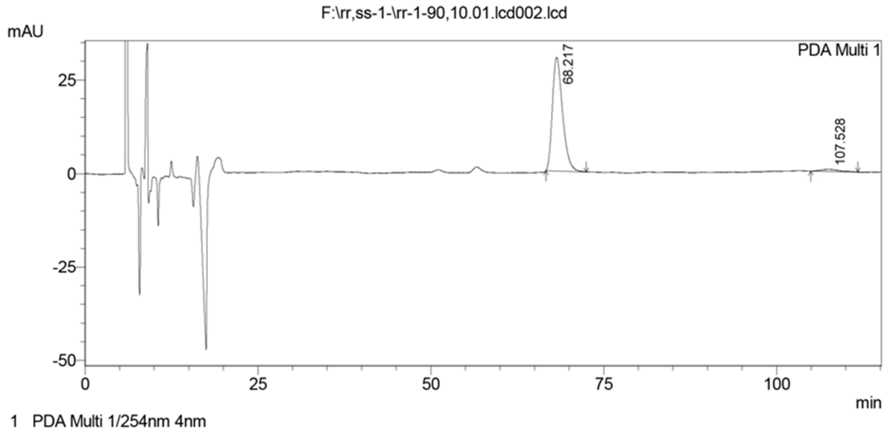


PeakTable

PDA Ch1 254nm 4nm

| Peak# | Ret. Time | Area | Height | Area % | Height % |
|---|---|---|---|---|---|
| 1 | 68.217 | 3104143 | 30404 | 96.562 | 97.955 |
| 2 | 107.528 | 110508 | 635 | 3.438 | 2.045 |
| Total | | 3214651 | 31039 | 100.000 | 100.000 |

**Compound (*S, S*)-12a**

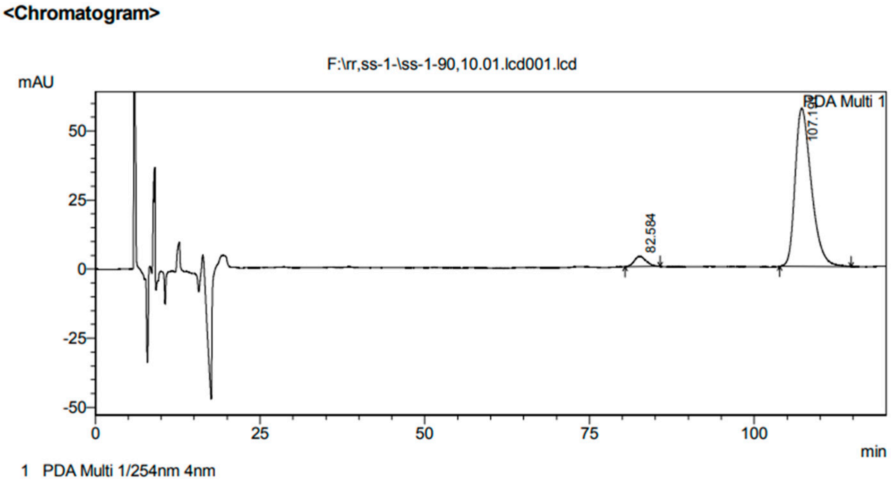


PeakTable

PDA Ch1 254nm 4nm

| Peak# | Ret. Time | Area | Height | Area % | Height % |
|---|---|---|---|---|---|
| 1 | 82.584 | 462538 | 3712 | 4.508 | 6.087 |
| 2 | 107.191 | 9798552 | 57264 | 95.492 | 93.913 |
| Total | | 10261090 | 60975 | 100.000 | 100.000 |

**Mixture of enantiomer (*R, S*)-12a and (*S, R*)-12a**

<Chromatogram>

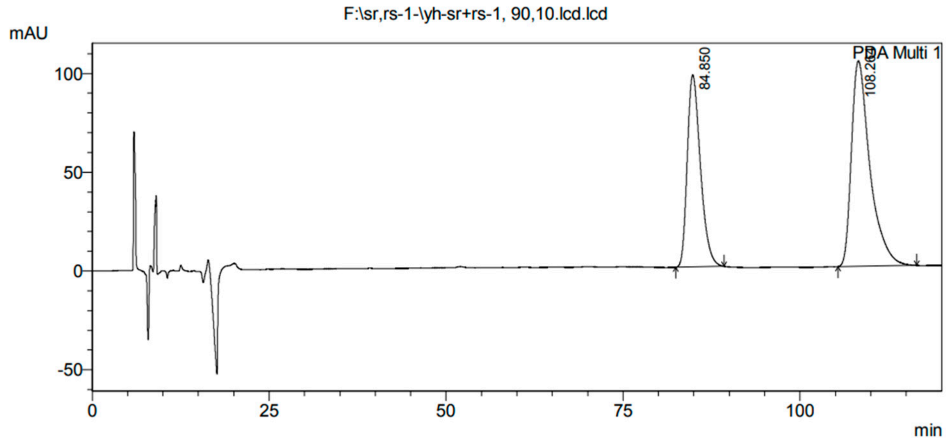


1 PDA Multi 1/254nm 4nm

PeakTable

PDA Ch1 254nm 4nm

| Peak# | Ret. Time | Area | Height | Area % | Height % |
|---|---|---|---|---|---|
| 1 | 84.850 | 12578785 | 97205 | 39.941 | 48.307 |
| 2 | 108.264 | 18914975 | 104017 | 60.059 | 51.693 |
| Total | | 31493760 | 201222 | 100.000 | 100.000 |

# Compound (*R, S*)-12a

<Chromatogram>

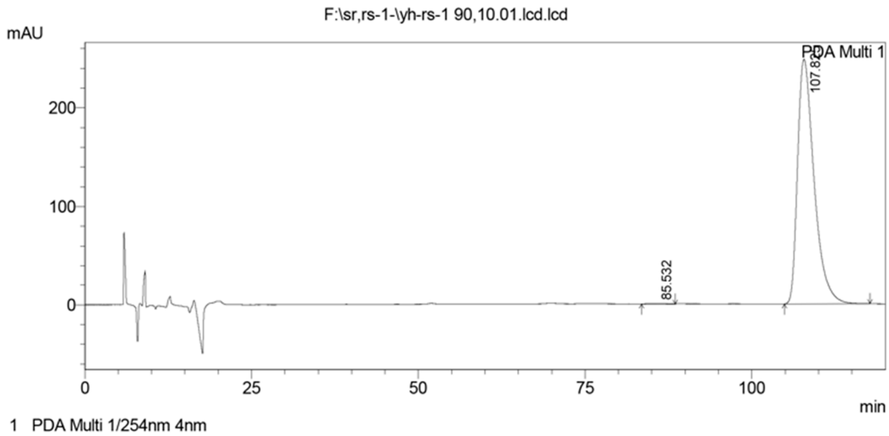


1 PDA Multi 1/254nm 4nm

PeakTable

PDA Ch1 254nm 4nm

| Peak# | Ret. Time | Area | Height | Area % | Height % |
|---|---|---|---|---|---|
| 1 | 85.532 | 57750 | 506 | 0.135 | 0.203 |
| 2 | 107.825 | 42804480 | 248374 | 99.865 | 99.797 |
| Total | | 42862230 | 248880 | 100.000 | 100.000 |

# Compound (*S, R*)-12a

<Chromatogram>

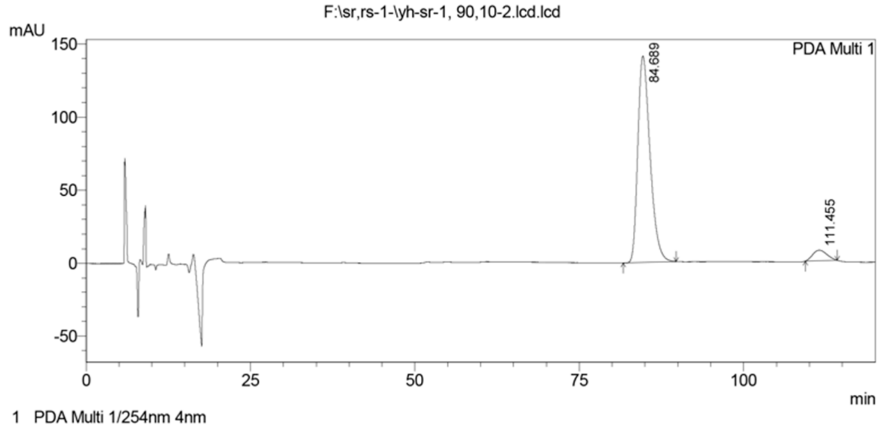


1 PDA Multi 1/254nm 4nm

PeakTable

PDA Ch1 254nm 4nm

| Peak# | Ret. Time | Area | Height | Area % | Height % |
|---|---|---|---|---|---|
| 1 | 84.689 | 18236735 | 141137 | 94.462 | 95.116 |
| 2 | 111.455 | 1069150 | 7247 | 5.538 | 4.884 |
| Total | | 19305885 | 148384 | 100.000 | 100.000 |

## Mixture of enantiomer (*R*, *R*)-12b and (*S*, *S*)-12b

<Chromatogram>

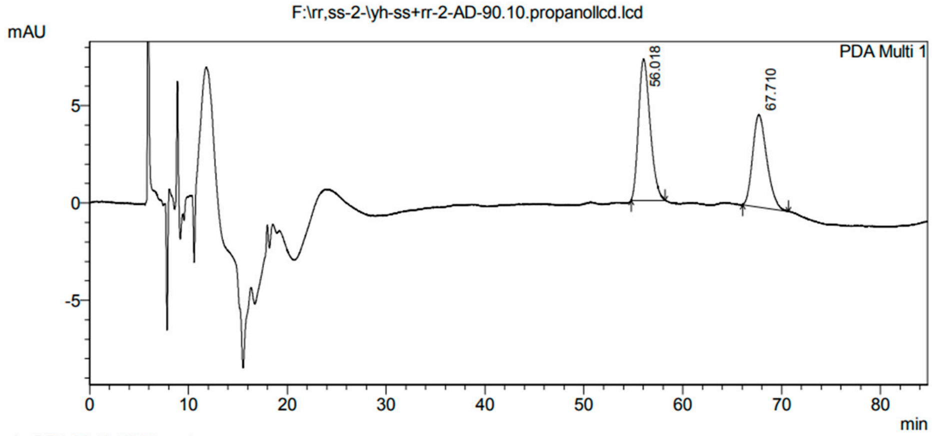


1 PDA Multi 1/254nm 4nm

PeakTable

PDA Ch1 254nm 4nm

| Peak# | Ret. Time | Area | Height | Area % | Height % |
|---|---|---|---|---|---|
| 1 | 56.018 | 593148 | 7266 | 55.474 | 60.335 |
| 2 | 67.710 | 476097 | 4777 | 44.526 | 39.665 |
| Total | | 1069245 | 12043 | 100.000 | 100.000 |

## Compound (*R*, *R*)-12b

<Chromatogram>

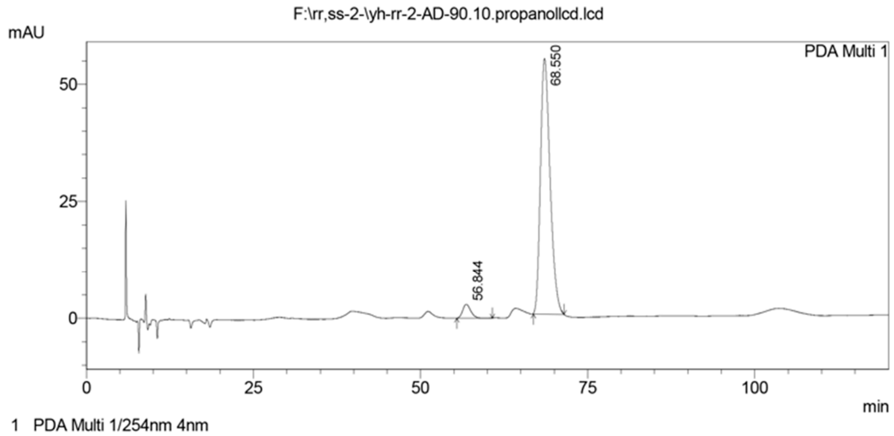


PeakTable

PDA Ch1 254nm 4nm

| Peak# | Ret. Time | Area | Height | Area % | Height % |
|---|---|---|---|---|---|
| 1 | 56.844 | 239685 | 2951 | 4.190 | 5.129 |
| 2 | 68.550 | 5480131 | 54587 | 95.810 | 94.871 |
| Total | | 5719816 | 57538 | 100.000 | 100.000 |

## Compound (*S*, *S*)-12b

<Chromatogram>

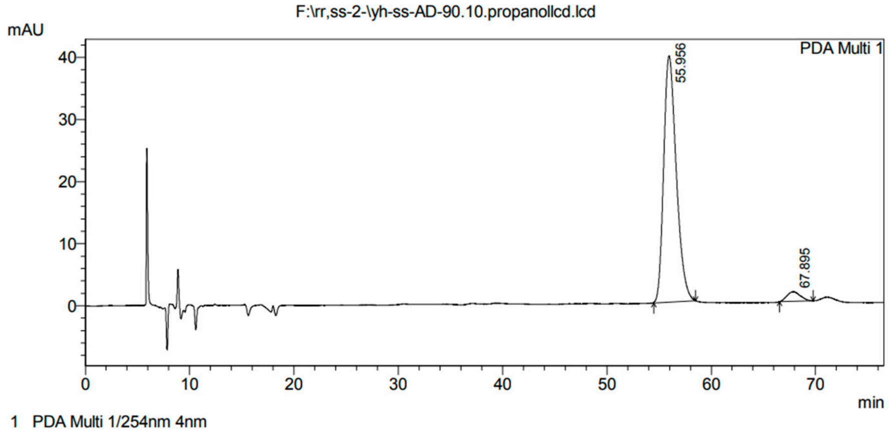


PeakTable

PDA Ch1 254nm 4nm

| Peak# | Ret. Time | Area | Height | Area % | Height % |
|---|---|---|---|---|---|
| 1 | 55.956 | 3326783 | 39683 | 96.087 | 96.272 |
| 2 | 67.895 | 135469 | 1537 | 3.913 | 3.728 |
| Total | | 3462252 | 41220 | 100.000 | 100.000 |

## Mixture of enantiomer (*R*, *S*)-12b and (*S*, *R*)-12b

<Chromatogram>

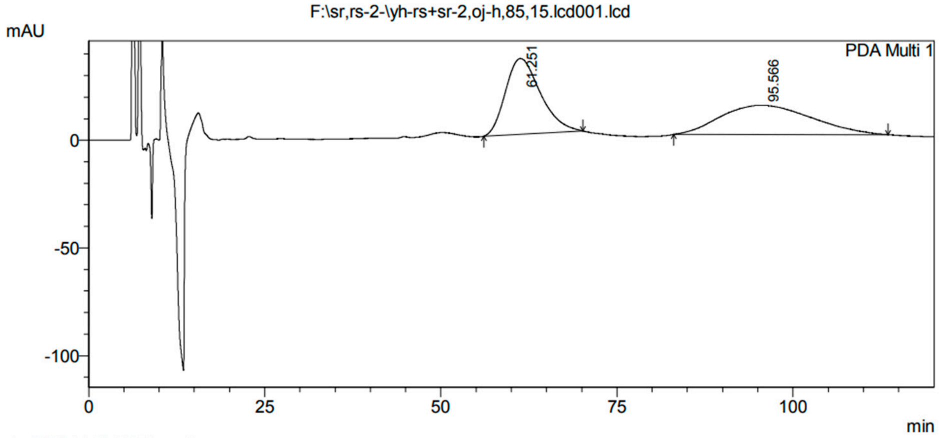


1 PDA Multi 1/254nm 4nm

PeakTable

PDA Ch1 254nm 4nm

| Peak# | Ret. Time | Area | Height | Area % | Height % |
|---|---|---|---|---|---|
| 1 | 61.251 | 12117155 | 35183 | 49.740 | 72.071 |
| 2 | 95.566 | 12243921 | 13634 | 50.260 | 27.929 |
| Total | | 24361076 | 48816 | 100.000 | 100.000 |

## Compound (*R, S*)-12b

<Chromatogram>

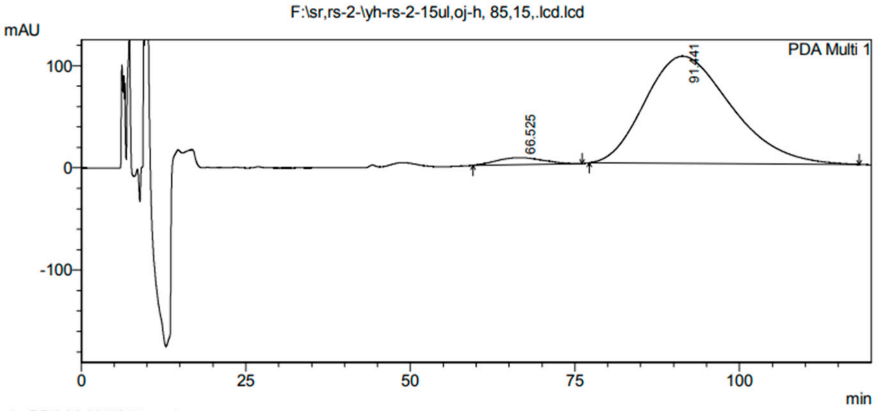


1 PDA Multi 1/254nm 4nm

PeakTable

PDA Ch1 254nm 4nm

| Peak# | Ret. Time | Area | Height | Area % | Height % |
|---|---|---|---|---|---|
| 1 | 66.525 | 3328016 | 6867 | 3.352 | 6.142 |
| 2 | 91.441 | 95963580 | 104929 | 96.648 | 93.858 |
| Total | | 99291596 | 111796 | 100.000 | 100.000 |

## Compound (*S, R*)-12b

**<Chromatogram>**

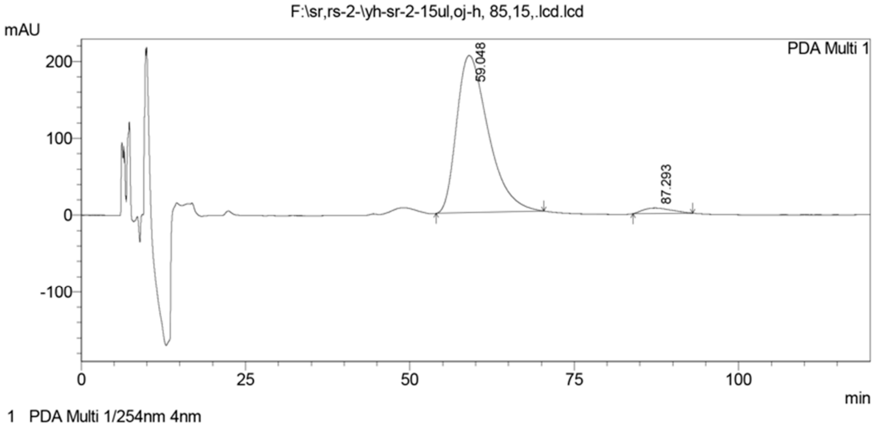


1 PDA Multi 1/254nm 4nm

PeakTable

PDA Ch1 254nm 4nm

| Peak# | Ret. Time | Area | Height | Area % | Height % |
|---|---|---|---|---|---|
| 1 | 59.048 | 68532370 | 204360 | 97.011 | 96.669 |
| 2 | 87.293 | 2111818 | 7042 | 2.989 | 3.331 |
| Total | | 70644188 | 211403 | 100.000 | 100.000 |

Compound (*R*, *R*)**-12a**



Data File: \\Deep-20160624ld\data1\黎星术\YH\RR-1.lcd

| Elmt | Val. | Min | Max | Elmt | Val. | Min | Max | Elmt | Val. | Min | Max | Use Adduct |
|---|---|---|---|---|---|---|---|---|---|---|---|---|
| H | 1 | 32 | 32 | P | 3 | 0 | 0 | Se | 2 | 0 | 0 | H |
| B | 3 | 0 | 0 | S | 2 | 0 | 0 | Br | 1 | 0 | 0 | Na |
| C | 4 | 31 | 31 | Cl | 1 | 0 | 10 | Ru | 2 | 0 | 0 | NH4 |
| N | 3 | 6 | 6 | Fe | 2 | 0 | 4 | Sb | 3 | 0 | 0 | K |
| O | 2 | 2 | 2 | Ni | 2 | 0 | 0 | I | 3 | 0 | 0 | |
| F | 1 | 0 | 0 | Cu | 2 | 0 | 0 | Ir | 3 | 0 | 0 | |
| Si | 4 | 0 | 0 | Zn | 2 | 0 | 0 | Pt | 2 | 0 | 0 | |

| | | | | | |
|---|---|---|---|---|---|
| Error Margin (mDa): | 20.0 | DBE Range: | not fixed | Electron Ions: | both |
| HC Ratio: | unlimited | Apply N Rule: | yes | Use MSn Info: | yes |
| Max Isotopes: | all | Isotope RI (%): | 1.00 | Isotope Res: | 10000 |
| MSn Iso RI (%): | 75.00 | MSn Logic Mode: | AND | Max Results: | 429 |

Event#: 1 MS(E+) Ret. Time : 0.720 Scan# : 109

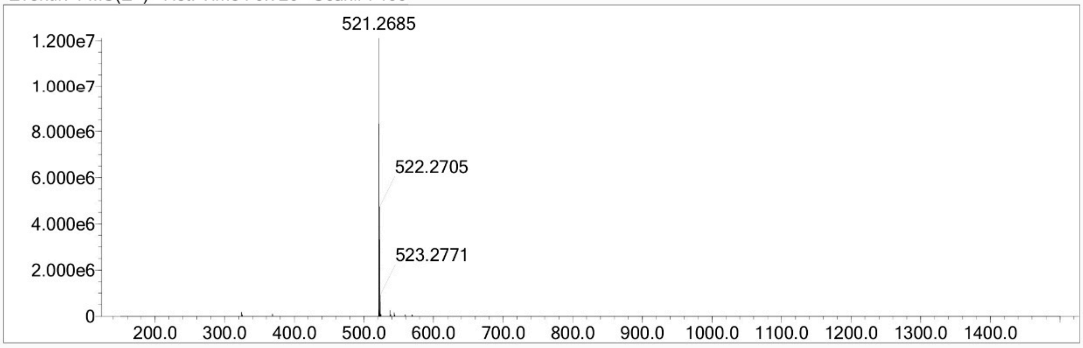


Measured region for 521.2685 m/z

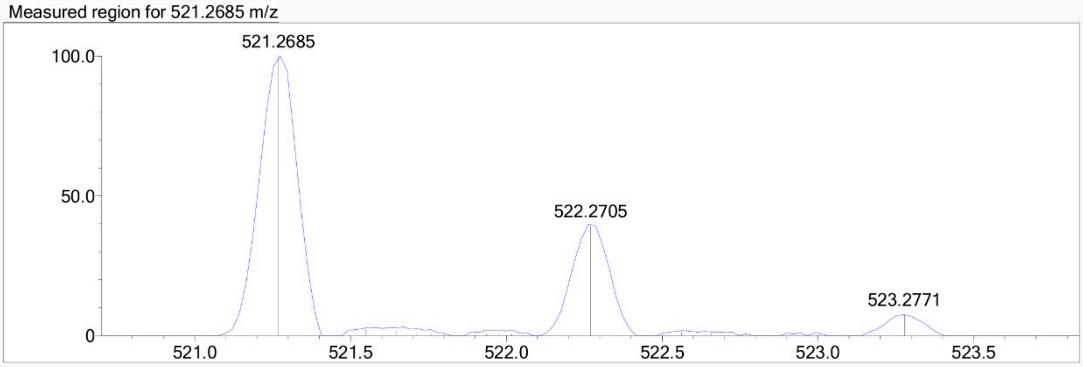


C31 H32 N6 O2 [M+H]+ : Predicted region for 521.2660 m/z

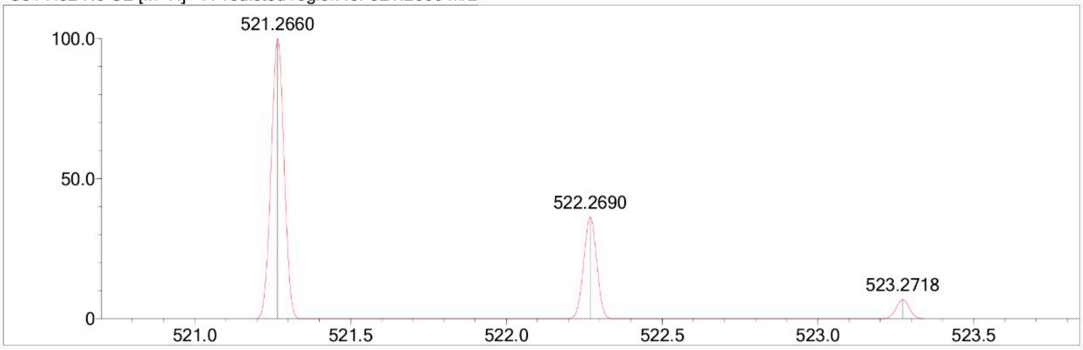


| Rank | Score | Formula (M) | Ion | Meas. m/z | Pred. m/z | Df. (mDa) | Df. (ppm) | Iso | DBE |
|---|---|---|---|---|---|---|---|---|---|
| 1 | 66.62 | C31 H32 N6 O2 | [M+H]+ | 521.2685 | 521.2660 | 2.5 | 4.80 | 73.62 | 19.0 |

Compound (*R*, *S*)**-12a**

Formula Predictor Report - RS-1.lcd Page 1 of 1

Data File: \\Deep-20160624ld\data1\黎星术\YH\RS-1.lcd

| Elmt | Val. | Min | Max | Elmt | Val. | Min | Max | Elmt | Val. | Min | Max | Use Adduct |
|---|---|---|---|---|---|---|---|---|---|---|---|---|
| H | 1 | 32 | 32 | P | 3 | 0 | 0 | Se | 2 | 0 | 0 | H |
| B | 3 | 0 | 0 | S | 2 | 0 | 0 | Br | 1 | 0 | 0 | Na |
| C | 4 | 31 | 31 | Cl | 1 | 0 | 10 | Ru | 2 | 0 | 0 | NH4 |
| N | 3 | 6 | 6 | Fe | 2 | 0 | 4 | Sb | 3 | 0 | 0 | K |
| O | 2 | 2 | 2 | Ni | 2 | 0 | 0 | I | 3 | 0 | 0 | |
| F | 1 | 0 | 0 | Cu | 2 | 0 | 0 | Ir | 3 | 0 | 0 | |
| Si | 4 | 0 | 0 | Zn | 2 | 0 | 0 | Pt | 2 | 0 | 0 | |

| | | | | | |
|---|---|---|---|---|---|
| Error Margin (mDa): | 20.0 | DBE Range: | not fixed | Electron Ions: | both |
| HC Ratio: | unlimited | Apply N Rule: | yes | Use MSn Info: | yes |
| Max Isotopes: | all | Isotope RI (%): | 1.00 | Isotope Res: | 10000 |
| MSn Iso RI (%): | 75.00 | MSn Logic Mode: | AND | Max Results: | 429 |

Event#: 1 MS(E+) Ret. Time : 0.640 Scan# : 97

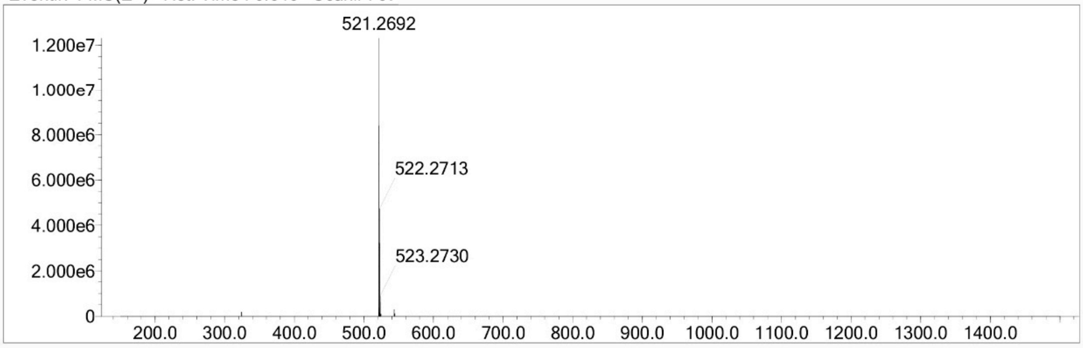


Measured region for 521.2692 m/z

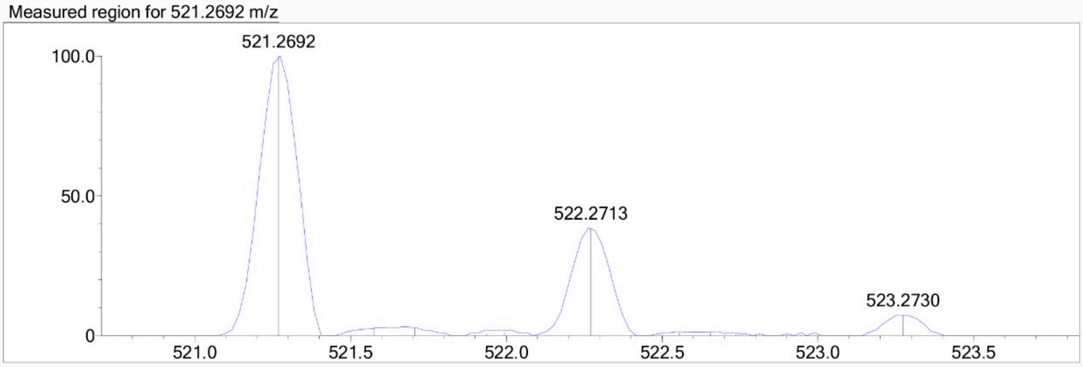


C31 H32 N6 O2 [M+H]+ : Predicted region for 521.2660 m/z

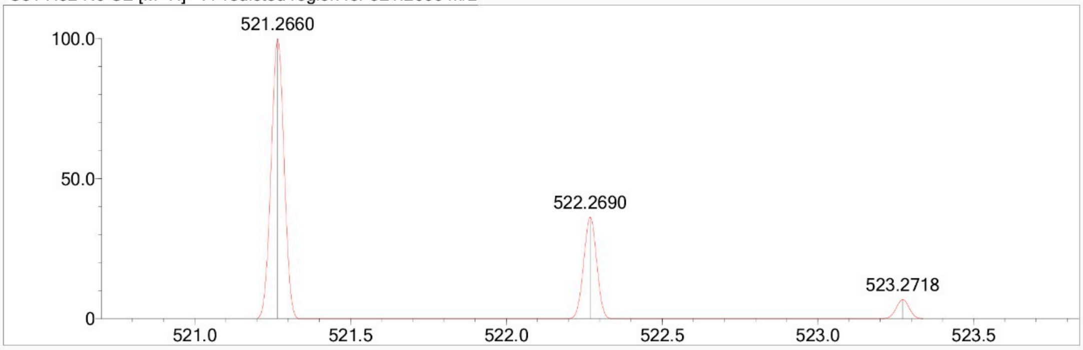


| Rank | Score | Formula (M) | Ion | Meas. m/z | Pred. m/z | Df. (mDa) | Df. (ppm) | Iso | DBE |
|---|---|---|---|---|---|---|---|---|---|
| 1 | 58.42 | C31 H32 N6 O2 | [M+H]+ | 521.2692 | 521.2660 | 3.2 | 6.14 | 74.32 | 19.0 |

## Compound(*S*, *R*)-12a

Formula Predictor Report - SR-1.lcd



Data File: \\Deep-20160624ld\data1\黎星术\YH\SR-1.lcd

| Elmt | Val. | Min | Max | Elmt | Val. | Min | Max | Elmt | Val. | Min | Max | Use Adduct |
|---|---|---|---|---|---|---|---|---|---|---|---|---|
| H | 1 | 32 | 32 | P | 3 | 0 | 0 | Se | 2 | 0 | 0 | H |
| B | 3 | 0 | 0 | S | 2 | 0 | 0 | Br | 1 | 0 | 0 | Na |
| C | 4 | 31 | 31 | Cl | 1 | 0 | 10 | Ru | 2 | 0 | 0 | NH4 |
| N | 3 | 6 | 6 | Fe | 2 | 0 | 4 | Sb | 3 | 0 | 0 | K |
| O | 2 | 2 | 2 | Ni | 2 | 0 | 0 | I | 3 | 0 | 0 | |
| F | 1 | 0 | 0 | Cu | 2 | 0 | 0 | Ir | 3 | 0 | 0 | |
| Si | 4 | 0 | 0 | Zn | 2 | 0 | 0 | Pt | 2 | 0 | 0 | |

| | | | | | |
|---|---|---|---|---|---|
| Error Margin (mDa): | 20.0 | DBE Range: | not fixed | Electron Ions: | both |
| HC Ratio: | unlimited | Apply N Rule: | yes | Use MSn Info: | yes |
| Max Isotopes: | all | Isotope RI (%): | 1.00 | Isotope Res: | 10000 |
| MSn Iso RI (%): | 75.00 | MSn Logic Mode: | AND | Max Results: | 429 |

Event#: 1 MS(E+) Ret. Time : 0.640 Scan# : 97

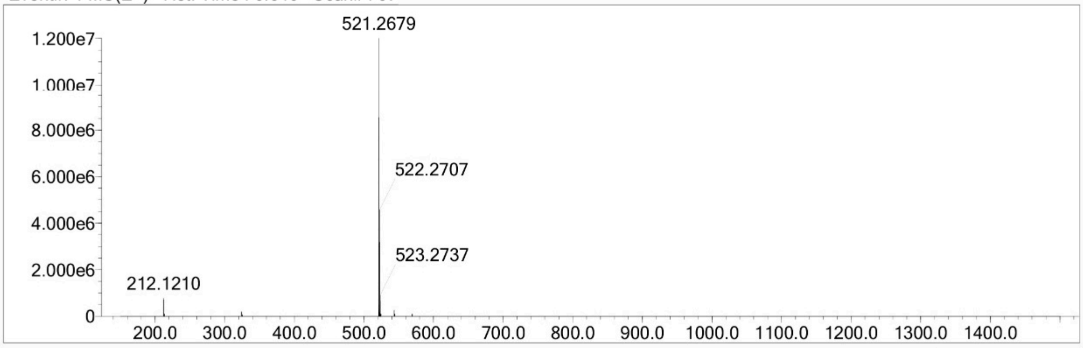


Measured region for 521.2679 m/z

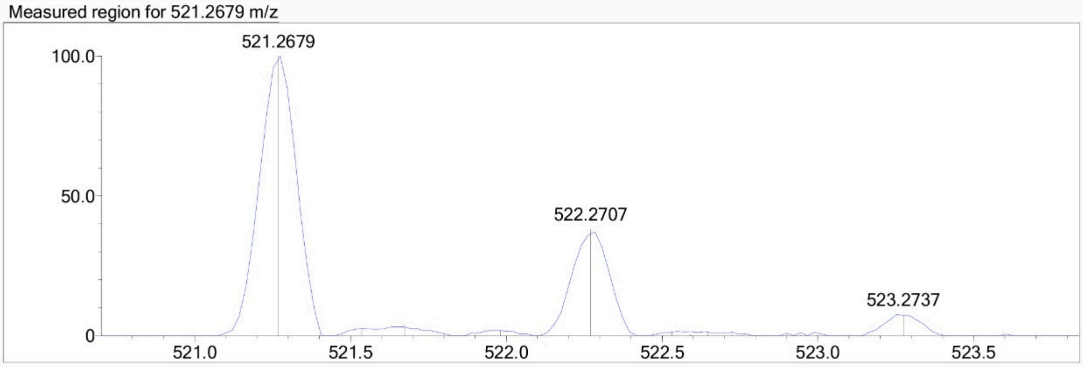


C31 H32 N6 O2 [M+H]+ : Predicted region for 521.2660 m/z

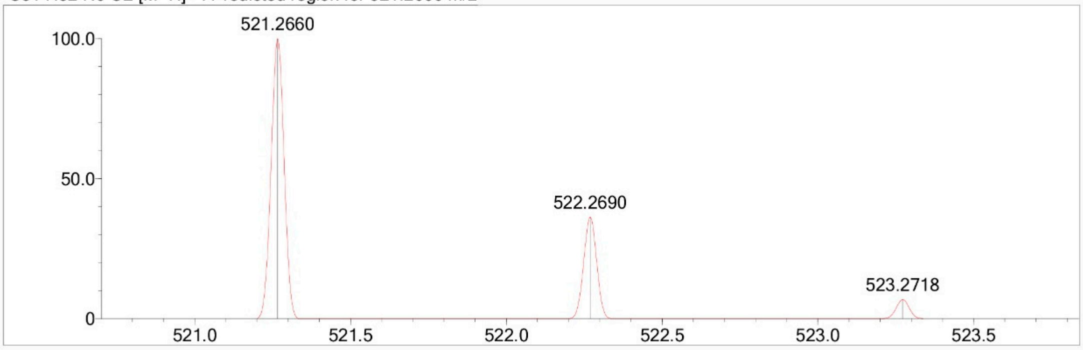


| Rank | Score | Formula (M) | Ion | Meas. m/z | Pred. m/z | Df. (mDa) | Df. (ppm) | Iso | DBE |
|---|---|---|---|---|---|---|---|---|---|
| 1 | 69.73 | C31 H32 N6 O2 | [M+H]+ | 521.2679 | 521.2660 | 1.9 | 3.64 | 74.65 | 19.0 |

## Compound(*S*, *S*)-12a

Formula Predictor Report - SS-1.lcd

Page 1 of 1

Data File: \\Deep-20160624ld\data1\黎星术\YH\SS-1.lcd

| Elmt | Val. | Min | Max | Elmt | Val. | Min | Max | Elmt | Val. | Min | Max | Use Adduct |
|---|---|---|---|---|---|---|---|---|---|---|---|---|
| H | 1 | 32 | 32 | P | 3 | 0 | 0 | Se | 2 | 0 | 0 | H |
| B | 3 | 0 | 0 | S | 2 | 0 | 0 | Br | 1 | 0 | 0 | Na |
| C | 4 | 31 | 31 | Cl | 1 | 0 | 10 | Ru | 2 | 0 | 0 | NH4 |
| N | 3 | 6 | 6 | Fe | 2 | 0 | 4 | Sb | 3 | 0 | 0 | K |
| O | 2 | 2 | 2 | Ni | 2 | 0 | 0 | I | 3 | 0 | 0 | |
| F | 1 | 0 | 0 | Cu | 2 | 0 | 0 | Ir | 3 | 0 | 0 | |
| Si | 4 | 0 | 0 | Zn | 2 | 0 | 0 | Pt | 2 | 0 | 0 | |

| | | | | | |
|---|---|---|---|---|---|
| Error Margin (mDa): | 20.0 | DBE Range: | not fixed | Electron Ions: | both |
| HC Ratio: | unlimited | Apply N Rule: | yes | Use MSn Info: | yes |
| Max Isotopes: | all | Isotope RI (%): | 1.00 | Isotope Res: | 10000 |
| MSn Iso RI (%): | 75.00 | MSn Logic Mode: | AND | Max Results: | 429 |

Event#: 1 MS(E+) Ret. Time : 0.693 Scan# : 105

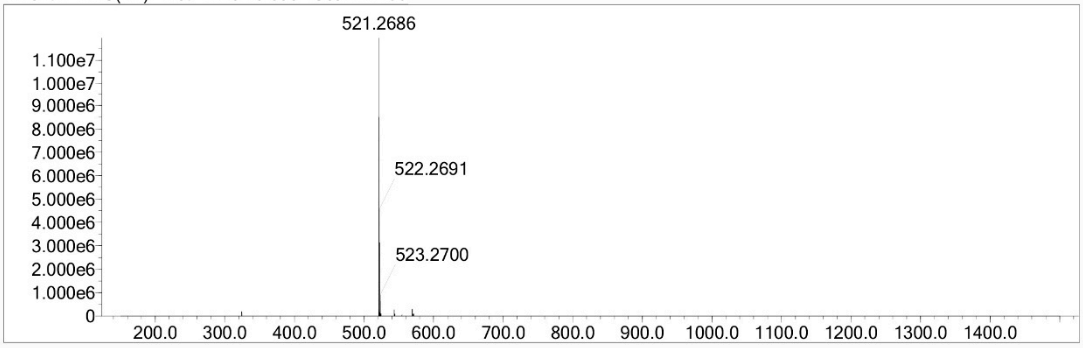


Measured region for 521.2686 m/z

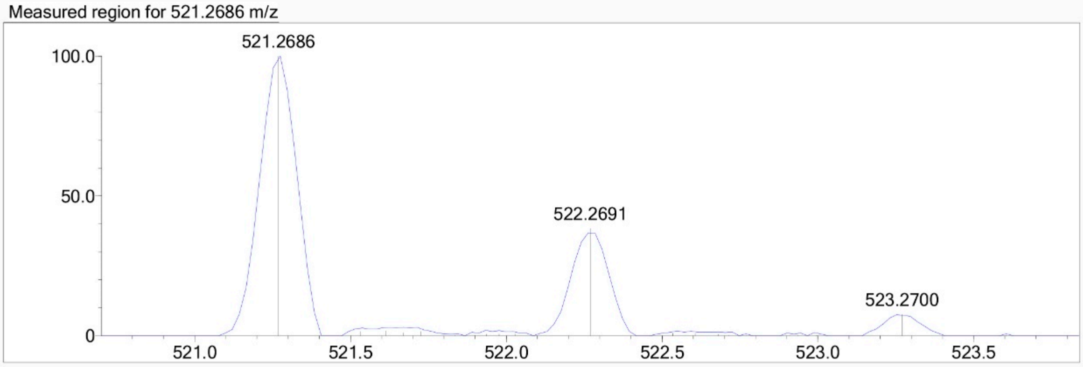


C31 H32 N6 O2 [M+H]+ : Predicted region for 521.2660 m/z

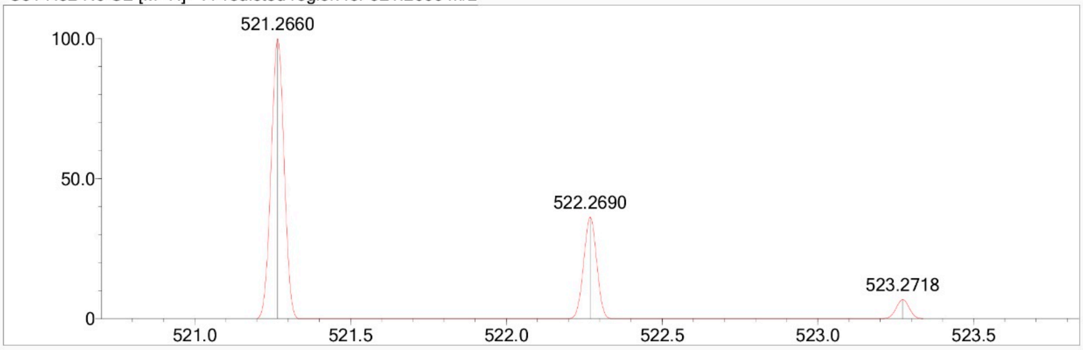


| Rank | Score | Formula (M) | Ion | Meas. m/z | Pred. m/z | Df. (mDa) | Df. (ppm) | Iso | DBE |
|---|---|---|---|---|---|---|---|---|---|
| 1 | 66.72 | C31 H32 N6 O2 | [M+H]+ | 521.2686 | 521.2660 | 2.6 | 4.99 | 74.12 | 19.0 |

## Compound(*R*, *R*)-12b

Formula Predictor Report - RR-2.lcd



Data File: \\Deep-20160624ld\data1\黎星术\YH\RR-2.lcd

| Elmt | Val. | Min | Max | Elmt | Val. | Min | Max | Elmt | Val. | Min | Max | Use Adduct |
|---|---|---|---|---|---|---|---|---|---|---|---|---|
| H | 1 | 30 | 30 | P | 3 | 0 | 0 | Se | 2 | 0 | 0 | H |
| B | 3 | 0 | 0 | S | 2 | 0 | 0 | Br | 1 | 0 | 0 | Na |
| C | 4 | 31 | 31 | Cl | 1 | 0 | 10 | Ru | 2 | 0 | 0 | NH4 |
| N | 3 | 6 | 6 | Fe | 2 | 0 | 4 | Sb | 3 | 0 | 0 | K |
| O | 2 | 2 | 2 | Ni | 2 | 0 | 0 | I | 3 | 0 | 0 | |
| F | 1 | 0 | 0 | Cu | 2 | 0 | 0 | Ir | 3 | 0 | 0 | |
| Si | 4 | 0 | 0 | Zn | 2 | 0 | 0 | Pt | 2 | 0 | 0 | |

| | | | | | |
|---|---|---|---|---|---|
| Error Margin (mDa): | 20.0 | DBE Range: | not fixed | Electron Ions: | both |
| HC Ratio: | unlimited | Apply N Rule: | yes | Use MSn Info: | yes |
| Max Isotopes: | all | Isotope RI (%): | 1.00 | Isotope Res: | 10000 |
| MSn Iso RI (%): | 75.00 | MSn Logic Mode: | AND | Max Results: | 429 |

Event#: 1 MS(E+) Ret. Time : 0.653 Scan# : 99

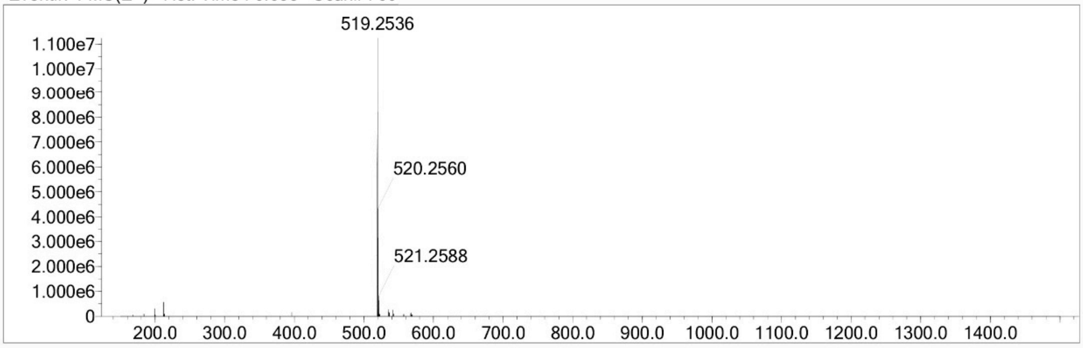


Measured region for 519.2536 m/z

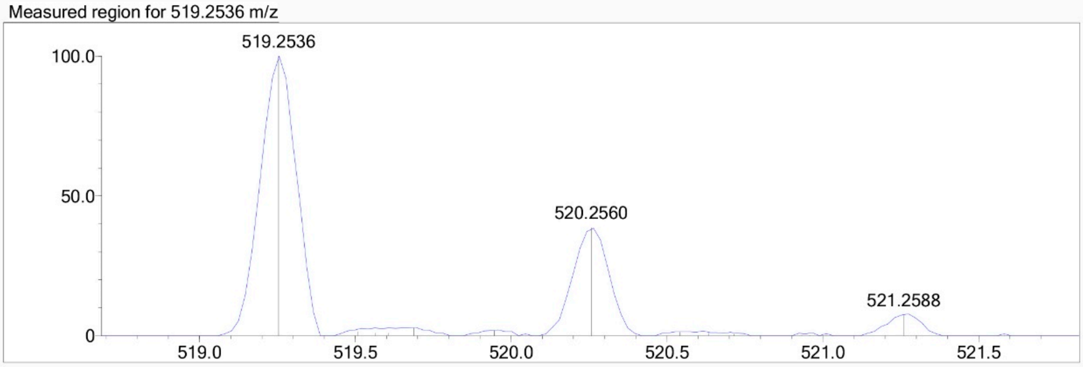


C31 H30 N6 O2 [M+H]+ : Predicted region for 519.2503 m/z

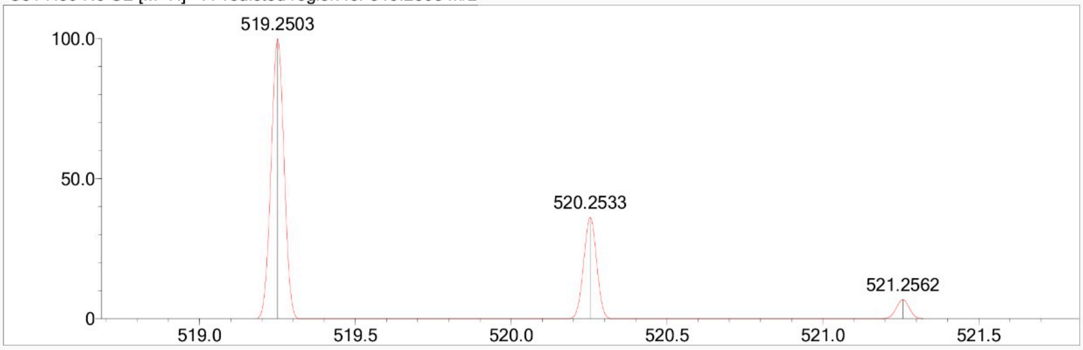


| Rank | Score | Formula (M) | Ion | Meas. m/z | Pred. m/z | Df. (mDa) | Df. (ppm) | Iso | DBE |
|---|---|---|---|---|---|---|---|---|---|
| 1 | 60.91 | C31 H30 N6 O2 | [M+H]+ | 519.2536 | 519.2503 | 3.3 | 6.36 | 79.73 | 20.0 |

Compound(*R*, *S*)-**12b**

Formula Predictor Report - RS-2.lcd



Data File: \\Deep-20160624ld\data1\黎星术\YH\RS-2.lcd

| Elmt | Val. | Min | Max | Elmt | Val. | Min | Max | Elmt | Val. | Min | Max | Use Adduct |
|---|---|---|---|---|---|---|---|---|---|---|---|---|
| H | 1 | 30 | 30 | P | 3 | 0 | 0 | Se | 2 | 0 | 0 | H |
| B | 3 | 0 | 0 | S | 2 | 0 | 0 | Br | 1 | 0 | 0 | Na |
| C | 4 | 31 | 31 | Cl | 1 | 0 | 10 | Ru | 2 | 0 | 0 | NH4 |
| N | 3 | 6 | 6 | Fe | 2 | 0 | 4 | Sb | 3 | 0 | 0 | K |
| O | 2 | 2 | 2 | Ni | 2 | 0 | 0 | I | 3 | 0 | 0 | |
| F | 1 | 0 | 0 | Cu | 2 | 0 | 0 | Ir | 3 | 0 | 0 | |
| Si | 4 | 0 | 0 | Zn | 2 | 0 | 0 | Pt | 2 | 0 | 0 | |

| | | | | | |
|---|---|---|---|---|---|
| Error Margin (mDa): | 20.0 | DBE Range: | not fixed | Electron Ions: | both |
| HC Ratio: | unlimited | Apply N Rule: | yes | Use MSn Info: | yes |
| Max Isotopes: | all | Isotope RI (%): | 1.00 | Isotope Res: | 10000 |
| MSn Iso RI (%): | 75.00 | MSn Logic Mode: | AND | Max Results: | 429 |

Event#: 1 MS(E+) Ret. Time : 0.653 Scan# : 99

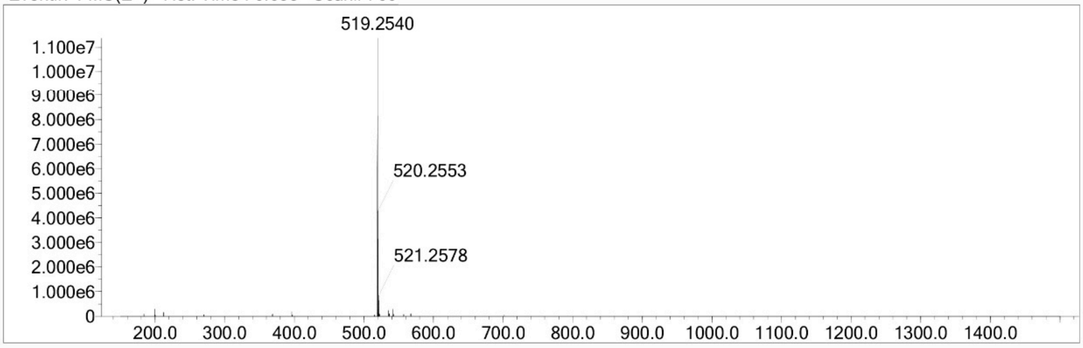


Measured region for 519.2540 m/z

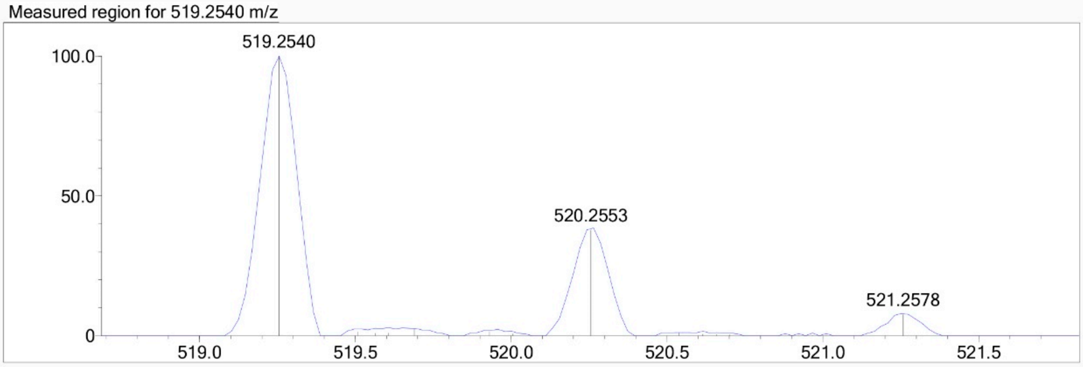


C31 H30 N6 O2 [M+H]+ : Predicted region for 519.2503 m/z

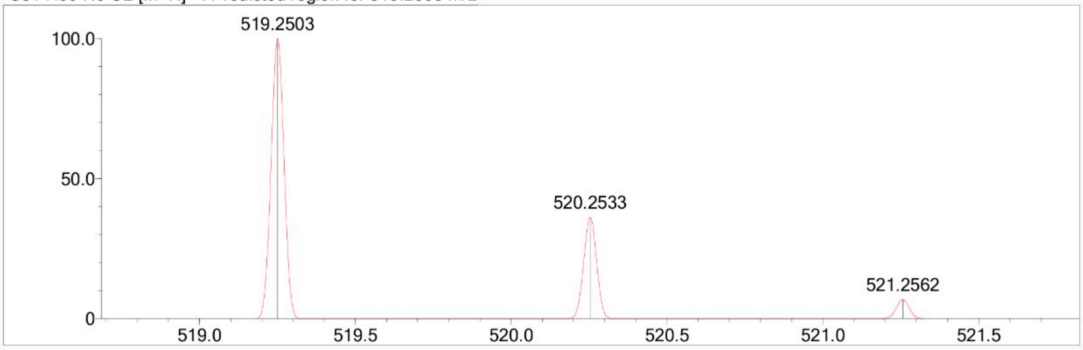


| Rank | Score | Formula (M) | Ion | Meas. m/z | Pred. m/z | Df. (mDa) | Df. (ppm) | Iso | DBE |
|---|---|---|---|---|---|---|---|---|---|
| 1 | 55.41 | C31 H30 N6 O2 | [M+H]+ | 519.2540 | 519.2503 | 3.7 | 7.13 | 80.65 | 20.0 |

## Compound(*S*, *R*)-12b

Formula Predictor Report - SR-2.lcd Page 1 of 1

Data File: \\Deep-20160624ld\data1\黎星术\YH\SR-2.lcd

| Elmt | Val. | Min | Max | Elmt | Val. | Min | Max | Elmt | Val. | Min | Max | Use Adduct |
|---|---|---|---|---|---|---|---|---|---|---|---|---|
| H | 1 | 30 | 30 | P | 3 | 0 | 0 | Se | 2 | 0 | 0 | H |
| B | 3 | 0 | 0 | S | 2 | 0 | 0 | Br | 1 | 0 | 0 | Na |
| C | 4 | 31 | 31 | Cl | 1 | 0 | 10 | Ru | 2 | 0 | 0 | NH4 |
| N | 3 | 6 | 6 | Fe | 2 | 0 | 4 | Sb | 3 | 0 | 0 | K |
| O | 2 | 2 | 2 | Ni | 2 | 0 | 0 | I | 3 | 0 | 0 | |
| F | 1 | 0 | 0 | Cu | 2 | 0 | 0 | Ir | 3 | 0 | 0 | |
| Si | 4 | 0 | 0 | Zn | 2 | 0 | 0 | Pt | 2 | 0 | 0 | |

| | | | | | |
|---|---|---|---|---|---|
| Error Margin (mDa): | 20.0 | DBE Range: | not fixed | Electron Ions: | both |
| HC Ratio: | unlimited | Apply N Rule: | yes | Use MSn Info: | yes |
| Max Isotopes: | all | Isotope RI (%): | 1.00 | Isotope Res: | 10000 |
| MSn Iso RI (%): | 75.00 | MSn Logic Mode: | AND | Max Results: | 429 |

Event#: 1 MS(E+) Ret. Time : 0.653 Scan# : 99

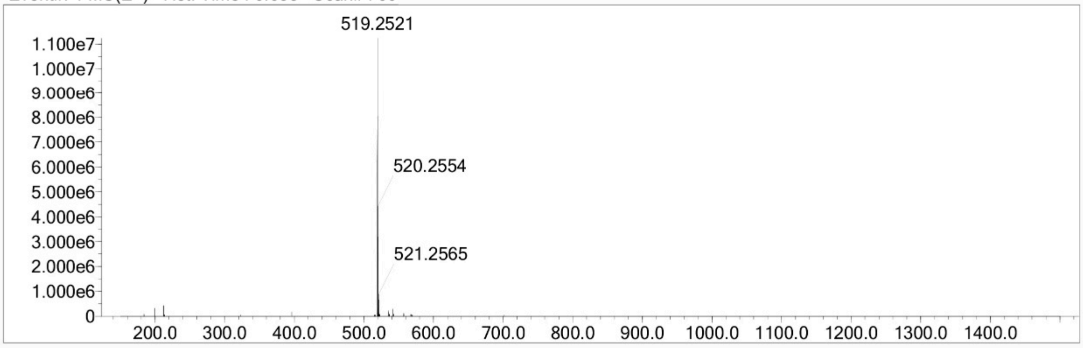


Measured region for 519.2521 m/z

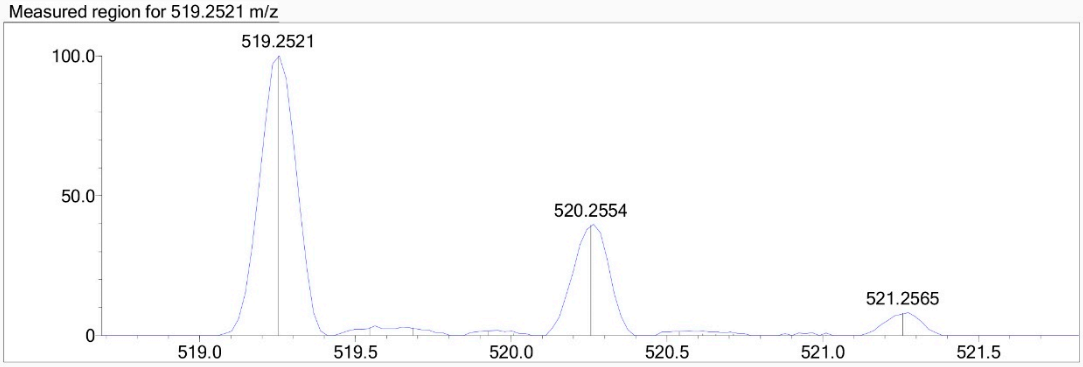


C31 H30 N6 O2 [M+H]+ : Predicted region for 519.2503 m/z

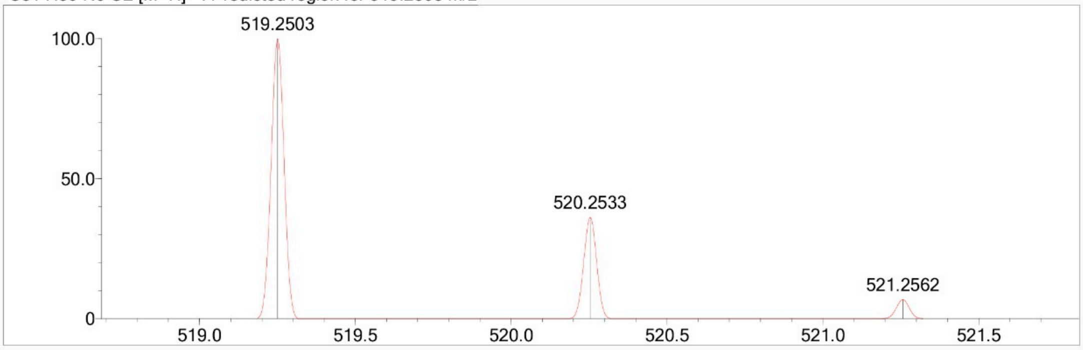


| Rank | Score | Formula (M) | Ion | Meas. m/z | Pred. m/z | Df. (mDa) | Df. (ppm) | Iso | DBE |
|---|---|---|---|---|---|---|---|---|---|
| 1 | 75.56 | C31 H30 N6 O2 | [M+H]+ | 519.2521 | 519.2503 | 1.8 | 3.47 | 80.53 | 20.0 |

## Compound(*S*, *S*)-12b



Data File: \\Deep-20160624ld\data1\黎星术\YH\SS-2.lcd

| Elmt | Val. | Min | Max | Elmt | Val. | Min | Max | Elmt | Val. | Min | Max | Use Adduct |
|---|---|---|---|---|---|---|---|---|---|---|---|---|
| H | 1 | 30 | 30 | P | 3 | 0 | 0 | Se | 2 | 0 | 0 | H |
| B | 3 | 0 | 0 | S | 2 | 0 | 0 | Br | 1 | 0 | 0 | Na |
| C | 4 | 31 | 31 | Cl | 1 | 0 | 10 | Ru | 2 | 0 | 0 | NH4 |
| N | 3 | 6 | 6 | Fe | 2 | 0 | 4 | Sb | 3 | 0 | 0 | K |
| O | 2 | 2 | 2 | Ni | 2 | 0 | 0 | I | 3 | 0 | 0 | |
| F | 1 | 0 | 0 | Cu | 2 | 0 | 0 | Ir | 3 | 0 | 0 | |
| Si | 4 | 0 | 0 | Zn | 2 | 0 | 0 | Pt | 2 | 0 | 0 | |

| | | | | | |
|---|---|---|---|---|---|
| Error Margin (mDa): | 20.0 | DBE Range: | not fixed | Electron Ions: | both |
| HC Ratio: | unlimited | Apply N Rule: | yes | Use MSn Info: | yes |
| Max Isotopes: | all | Isotope RI (%): | 1.00 | Isotope Res: | 10000 |
| MSn Iso RI (%): | 75.00 | MSn Logic Mode: | AND | Max Results: | 429 |

Event#: 1 MS(E+) Ret. Time : 1.147 Scan# : 173

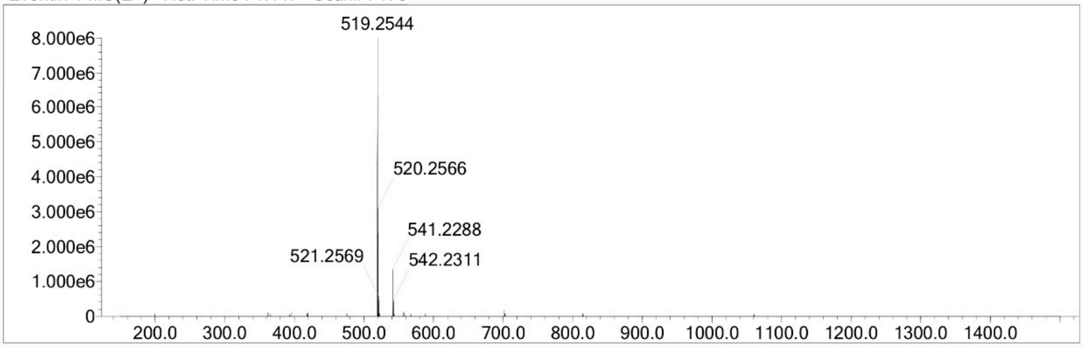


Measured region for 519.2544 m/z

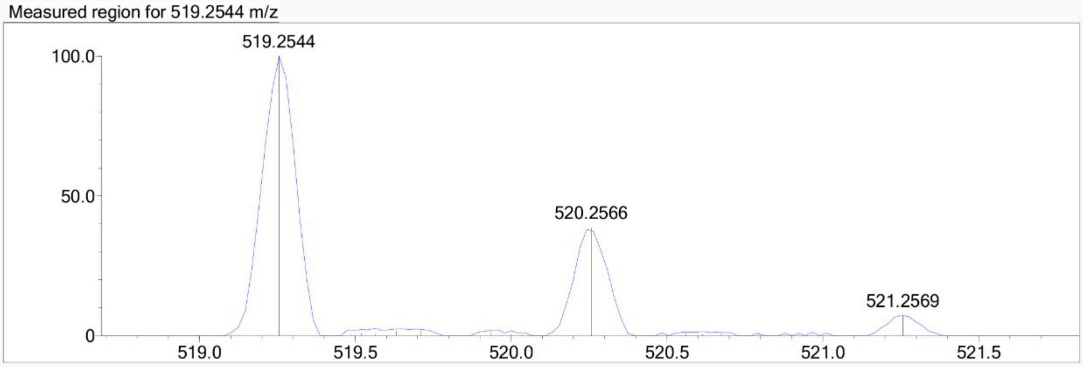


C31 H30 N6 O2 [M+H]+ : Predicted region for 519.2503 m/z

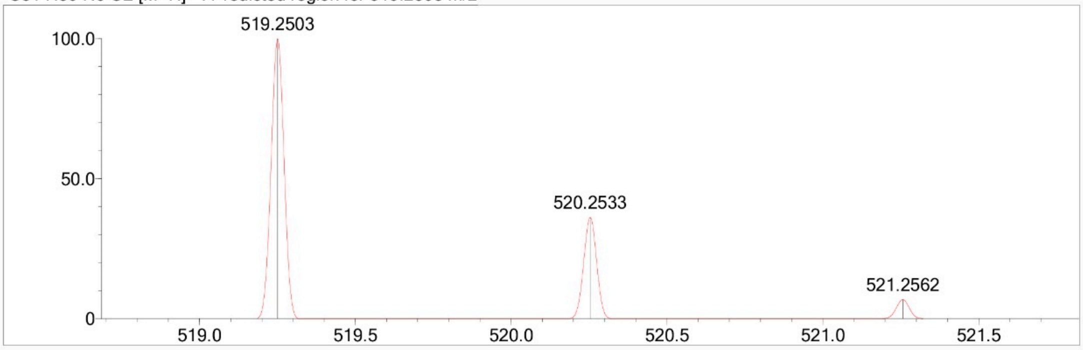


| Rank | Score | Formula (M) | Ion | Meas. m/z | Pred. m/z | Df. (mDa) | Df. (ppm) | Iso | DBE |
|---|---|---|---|---|---|---|---|---|---|
| 1 | 51.23 | C31 H30 N6 O2 | [M+H]+ | 519.2544 | 519.2503 | 4.1 | 7.90 | 83.98 | 20.0 |

Hy0102 representing compound **R-5b**

**Table S1 Crystal data and structure refinement for hy0102.**

| | |
|---|---|
| Identification code | hy0102 |
| Empirical formula | $C_{30}H_{31}ClN_6O_2$ |
| Formula weight | 543.06 |
| Temperature/K | 169.99(10) |
| Crystal system | monoclinic |
| Space group | $P2_1$ |
| a/Å | 13.7983(8) |
| b/Å | 7.3771(3) |
| c/Å | 14.3578(7) |
| α/° | 90 |
| β/° | 101.730(5) |
| γ/° | 90 |
| Volume/Å$^3$ | 1430.97(12) |
| Z | 2 |
| $\rho_{calc}$g/cm$^3$ | 1.260 |
| μ/mm$^{-1}$ | 1.482 |
| F(000) | 572.0 |
| Crystal size/mm$^3$ | 0.15 × 0.11 × 0.09 |

| | |
|---|---|
| Radiation | Cu Kα (λ = 1.54184) |
| 2Θ range for data collection/° | 6.288 to 133.078 |
| Index ranges | $-16 \leq h \leq 14$, $-5 \leq k \leq 8$, $-17 \leq l \leq 16$ |
| Reflections collected | 5010 |
| Independent reflections | 3735 [$R_{int}$ = 0.0499, $R_{sigma}$ = 0.0818] |
| Data/restraints/parameters | 3735/1/354 |
| Goodness-of-fit on $F^2$ | 1.018 |
| Final R indexes [I>=2σ (I)] | $R_1$ = 0.0529, $wR_2$ = 0.1253 |
| Final R indexes [all data] | $R_1$ = 0.0592, $wR_2$ = 0.1330 |
| Largest diff. peak/hole / e Å$^{-3}$ | 0.30/-0.34 |
| Flack parameter | 0.02(3) |

**Crystal structure determination of [hy0102]**

**Crystal Data** for $C_{30}H_{31}ClN_6O_2$ ($M$ =543.06 g/mol): monoclinic, space group $P2_1$ (no. 4), $a$ = 13.7983(8) Å, $b$ = 7.3771(3) Å, $c$ = 14.3578(7) Å, $\beta$ = 101.730(5)°, $V$ = 1430.97(12) Å$^3$, $Z$ = 2, $T$ = 169.99(10) K, μ(Cu Kα) = 1.482 mm$^{-1}$, $Dcalc$ = 1.260 g/cm$^3$, 5010 reflections measured (6.288° ≤ 2Θ ≤ 133.078°), 3735 unique ($R_{int}$ = 0.0499, $R_{sigma}$ = 0.0818) which were used in all calculations. The final $R_1$ was 0.0529 (I > 2σ(I)) and $wR_2$ was 0.1330 (all data).

**Refinement model description**

**Table S2 Fractional Atomic Coordinates (×10$^4$) and Equivalent Isotropic Displacement Parameters (Å$^2$×10$^3$) for hy0102. $U_{eq}$ is defined as 1/3 of the trace of the orthogonalised $U_{IJ}$ tensor.**

| Atom | *x* | *y* | *z* | U(eq) |
|---|---|---|---|---|
| Cl1 | 9110.0(9) | -255.3(16) | 1009.5(8) | 32.6(3) |
| O1 | 5324(3) | 5668(6) | 9674(2) | 43.5(10) |
| O2 | 7510(2) | 5542(5) | 3237(2) | 30.0(8) |
| N1 | 4192(6) | 8460(8) | 3222(4) | 73(2) |
| N2 | 4389(3) | 7162(5) | 7318(3) | 25.1(9) |
| N3 | 4901(3) | 7052(5) | 8223(3) | 23.5(8) |
| N4 | 6661(3) | 6914(6) | 5333(3) | 25.6(9) |
| N5 | 8371(3) | 6173(6) | 5796(3) | 28.8(9) |
| N6 | 8297(3) | 3325(6) | 79(3) | 27.0(9) |
| C1 | 3176(4) | 6144(7) | 6012(4) | 32.5(12) |
| C2 | 3692(4) | 6829(7) | 5351(4) | 32.7(12) |
| C3 | 3207(4) | 7062(8) | 4410(4) | 41.0(14) |
| C4 | 2215(5) | 6614(9) | 4114(4) | 50.3(16) |
| C5 | 1717(5) | 5922(8) | 4768(5) | 52.0(17) |
| C6 | 2179(4) | 5685(8) | 5722(4) | 41.4(14) |
| C7 | 3748(5) | 7843(9) | 3742(4) | 50.4(16) |

**Table S2 Fractional Atomic Coordinates (×10[4]) and Equivalent Isotropic Displacement Parameters (Å[2]×10[3]) for hy0102. $U_{eq}$ is defined as 1/3 of the trace of the orthogonalised $U_{IJ}$ tensor.**

| **Atom** | ***x*** | ***y*** | ***z*** | **U(eq)** |
|---|---|---|---|---|
| C8 | 3690(4) | 5959(7) | 7020(3) | 27.6(11) |
| C9 | 3473(4) | 4569(8) | 7634(4) | 36.3(12) |
| C10 | 4008(4) | 4440(7) | 8529(4) | 38.0(13) |
| C11 | 4781(4) | 5694(7) | 8871(3) | 32.3(12) |
| C12 | 5636(3) | 8498(7) | 8518(3) | 26.0(10) |
| C13 | 5211(4) | 10328(7) | 8173(4) | 31.7(11) |
| C14 | 6591(3) | 8105(6) | 8193(3) | 24.6(10) |
| C15 | 7487(3) | 8343(7) | 8836(3) | 27.4(11) |
| C16 | 8366(4) | 8069(7) | 8552(3) | 30.3(11) |
| C17 | 8395(4) | 7564(7) | 7627(3) | 30.4(11) |
| C18 | 7498(3) | 7322(6) | 6969(3) | 22.5(10) |
| C19 | 6613(4) | 7621(7) | 7257(3) | 26.1(10) |
| C20 | 7522(3) | 6757(7) | 5984(3) | 26.1(10) |
| C22 | 8395(4) | 5727(7) | 4894(3) | 30.6(11) |
| C23 | 7551(3) | 5896(7) | 4176(3) | 25.3(10) |
| C24 | 6695(4) | 6463(7) | 4443(3) | 27.4(11) |

**Table S2 Fractional Atomic Coordinates (×10$^4$) and Equivalent Isotropic Displacement Parameters (Å$^2$×10$^3$) for hy0102. $U_{eq}$ is defined as 1/3 of the trace of the orthogonalised $U_{IJ}$ tensor.**

| Atom | *x* | *y* | *z* | U(eq) |
|---|---|---|---|---|
| C26 | 8430(3) | 5167(7) | 2954(3) | 28.4(12) |
| C27 | 8217(3) | 5128(6) | 1873(3) | 26.0(11) |
| C28 | 9182(3) | 4896(7) | 1532(3) | 25.6(10) |
| C29 | 8979(3) | 4845(7) | 448(3) | 26.2(10) |
| C30 | 7333(4) | 3508(8) | 390(3) | 34.4(12) |
| C31 | 7512(4) | 3605(8) | 1476(3) | 31.4(11) |
| C32 | 8139(4) | 3198(8) | -988(3) | 40.0(13) |

**Table S3 Anisotropic Displacement Parameters (Å$^2$×10$^3$) for hy0102. The Anisotropic displacement factor exponent takes the form: -2π$^2$[h$^2$a*$^2$U$_{11}$+2hka*b*U$_{12}$+…].**

| Atom | $U_{11}$ | $U_{22}$ | $U_{33}$ | $U_{23}$ | $U_{13}$ | $U_{12}$ |
|---|---|---|---|---|---|---|
| Cl1 | 44.5(7) | 28.5(6) | 23.4(5) | -1.5(5) | 3.7(4) | 2.2(6) |
| O1 | 56(2) | 45(2) | 29.1(19) | 13.6(18) | 7.1(17) | 9.9(19) |
| O2 | 30.8(18) | 43(2) | 16.9(15) | -3.6(15) | 7.2(13) | 6.8(16) |
| N1 | 138(6) | 45(3) | 41(3) | 3(3) | 30(4) | 22(4) |

**Table S3 Anisotropic Displacement Parameters (Å$^2$×10$^3$) for hy0102. The Anisotropic displacement factor exponent takes the form: -2π$^2$[h$^2$a*$^2$U$_{11}$+2hka*b*U$_{12}$+…].**

| Atom | U11 | U22 | U33 | U23 | U13 | U12 |
|---|---|---|---|---|---|---|
| N2 | 32(2) | 22(2) | 21.0(19) | -1.9(16) | 5.5(16) | 0.0(17) |
| N3 | 32(2) | 22(2) | 18.9(18) | 1.7(17) | 10.3(16) | 0.9(17) |
| N4 | 28(2) | 32(2) | 16.8(19) | -2.1(18) | 5.8(16) | 1.3(18) |
| N5 | 28(2) | 37(3) | 22(2) | -2.0(18) | 6.7(16) | 3.1(18) |
| N6 | 37(2) | 25(2) | 19.5(19) | -1.3(17) | 6.0(16) | 1.5(18) |
| C1 | 33(3) | 25(3) | 38(3) | -9(2) | 4(2) | 3(2) |
| C2 | 40(3) | 27(3) | 29(3) | -5(2) | 2(2) | 4(2) |
| C3 | 56(4) | 33(3) | 32(3) | -6(3) | 5(3) | 8(3) |
| C4 | 60(4) | 45(4) | 39(3) | -7(3) | -6(3) | 5(3) |
| C5 | 43(3) | 41(4) | 61(4) | -16(3) | -16(3) | 0(3) |
| C6 | 38(3) | 32(3) | 52(3) | -6(3) | 5(3) | -6(2) |
| C7 | 77(5) | 43(4) | 29(3) | -7(3) | 7(3) | 15(3) |
| C8 | 28(2) | 22(2) | 33(3) | 0(2) | 8(2) | -1(2) |
| C9 | 47(3) | 28(3) | 39(3) | -4(3) | 19(2) | -10(2) |
| C10 | 58(3) | 25(3) | 37(3) | 4(2) | 24(3) | -2(2) |
| C11 | 44(3) | 29(3) | 28(3) | 8(2) | 17(2) | 8(2) |

**Table S3 Anisotropic Displacement Parameters ($Å^2 \times 10^3$) for hy0102. The Anisotropic displacement factor exponent takes the form: -$2\pi^2[h^2a^{*2}U_{11}+2hka^*b^*U_{12}+…]$.**

| Atom | $U_{11}$ | $U_{22}$ | $U_{33}$ | $U_{23}$ | $U_{13}$ | $U_{12}$ |
|---|---|---|---|---|---|---|
| C12 | 28(3) | 28(2) | 21(2) | -4(2) | 4.1(19) | -1(2) |
| C13 | 35(3) | 22(3) | 38(3) | -3(2) | 8(2) | 4(2) |
| C14 | 27(2) | 26(3) | 21(2) | -0.6(19) | 6.2(19) | 0(2) |
| C15 | 37(3) | 25(3) | 20(2) | -1(2) | 5(2) | -4(2) |
| C16 | 29(3) | 38(3) | 21(2) | -2(2) | -2.7(19) | -6(2) |
| C17 | 27(2) | 38(3) | 27(2) | -3(2) | 7(2) | -6(2) |
| C18 | 25(2) | 27(2) | 14(2) | -0.3(18) | 0.7(17) | -4(2) |
| C19 | 30(2) | 27(3) | 20(2) | -2(2) | 3.8(19) | 0(2) |
| C20 | 29(2) | 30(3) | 18(2) | 0(2) | 1.1(18) | -5(2) |
| C22 | 30(3) | 36(3) | 27(2) | 0(2) | 6(2) | 9(2) |
| C23 | 32(3) | 28(3) | 16(2) | 1(2) | 4.0(18) | 3(2) |
| C24 | 28(2) | 34(3) | 21(2) | 1(2) | 6.4(19) | -2(2) |
| C26 | 32(3) | 33(3) | 21(2) | -1(2) | 7.7(19) | 3(2) |
| C27 | 32(2) | 27(3) | 19(2) | 1.1(19) | 7.0(19) | 3(2) |
| C28 | 29(2) | 27(2) | 20(2) | -1(2) | 3.3(18) | 3(2) |
| C29 | 31(2) | 27(2) | 22(2) | -1(2) | 8.2(18) | 0(2) |

**Table S3 Anisotropic Displacement Parameters ($Å^2×10^3$) for hy0102. The Anisotropic displacement factor exponent takes the form: -$2\pi^2[h^2a^{*2}U_{11}+2hka^*b^*U_{12}+…]$.**

| Atom | $U_{11}$ | $U_{22}$ | $U_{33}$ | $U_{23}$ | $U_{13}$ | $U_{12}$ |
|---|---|---|---|---|---|---|
| C30 | 30(3) | 46(3) | 28(3) | -6(2) | 5(2) | -1(2) |
| C31 | 31(3) | 38(3) | 27(2) | -7(2) | 12(2) | -4(2) |
| C32 | 60(4) | 39(3) | 22(3) | -4(2) | 10(2) | -3(3) |

**Table S4 Bond Lengths for hy0102.**

| Atom | Atom | Length/Å | Atom | Atom | Length/Å |
|---|---|---|---|---|---|
| O1 | C11 | 1.240(6) | C4 | C5 | 1.370(9) |
| O2 | C23 | 1.363(5) | C5 | C6 | 1.398(8) |
| O2 | C26 | 1.436(5) | C8 | C9 | 1.423(7) |
| N1 | C7 | 1.152(8) | C9 | C10 | 1.349(7) |
| N2 | N3 | 1.350(5) | C10 | C11 | 1.422(7) |
| N2 | C8 | 1.316(6) | C12 | C13 | 1.515(7) |
| N3 | C11 | 1.400(6) | C12 | C14 | 1.513(6) |
| N3 | C12 | 1.474(6) | C14 | C15 | 1.395(6) |
| N4 | C20 | 1.358(6) | C14 | C19 | 1.397(6) |
| N4 | C24 | 1.331(6) | C15 | C16 | 1.372(7) |

**Table S4 Bond Lengths for hy0102.**

| Atom | Atom | Length/Å | Atom | Atom | Length/Å |
|---|---|---|---|---|---|
| N5 | C20 | 1.327(6) | C16 | C17 | 1.387(7) |
| N5 | C22 | 1.342(6) | C17 | C18 | 1.407(6) |
| N6 | C29 | 1.490(6) | C18 | C19 | 1.385(6) |
| N6 | C30 | 1.494(6) | C18 | C20 | 1.481(6) |
| N6 | C32 | 1.505(6) | C22 | C23 | 1.395(6) |
| C1 | C2 | 1.393(7) | C23 | C24 | 1.379(7) |
| C1 | C6 | 1.396(7) | C26 | C27 | 1.520(6) |
| C1 | C8 | 1.483(7) | C27 | C28 | 1.519(6) |
| C2 | C3 | 1.391(7) | C27 | C31 | 1.519(6) |
| C3 | C4 | 1.388(8) | C28 | C29 | 1.525(5) |
| C3 | C7 | 1.448(9) | C30 | C31 | 1.530(6) |

**Table S5 Bond Angles for hy0102.**

| Atom | Atom | Atom | Angle/° | Atom | Atom | Atom | Angle/° |
|---|---|---|---|---|---|---|---|
| C23 | O2 | C26 | 117.1(3) | N3 | C12 | C13 | 110.7(4) |
| C8 | N2 | N3 | 118.5(4) | N3 | C12 | C14 | 111.4(4) |
| N2 | N3 | C11 | 125.0(4) | C14 | C12 | C13 | 111.9(4) |

**Table S5 Bond Angles for hy0102.**

| Atom | Atom | Atom | Angle/° | Atom | Atom | Atom | Angle/° |
|---|---|---|---|---|---|---|---|
| N2 | N3 | C12 | 115.4(4) | C15 | C14 | C12 | 118.8(4) |
| C11 | N3 | C12 | 119.6(4) | C15 | C14 | C19 | 118.7(4) |
| C24 | N4 | C20 | 116.3(4) | C19 | C14 | C12 | 122.4(4) |
| C20 | N5 | C22 | 118.1(4) | C16 | C15 | C14 | 120.2(4) |
| C29 | N6 | C30 | 111.2(4) | C15 | C16 | C17 | 121.6(4) |
| C29 | N6 | C32 | 111.1(4) | C16 | C17 | C18 | 119.0(5) |
| C30 | N6 | C32 | 110.9(4) | C17 | C18 | C20 | 119.4(4) |
| C2 | C1 | C6 | 119.5(5) | C19 | C18 | C17 | 119.2(4) |
| C2 | C1 | C8 | 119.3(5) | C19 | C18 | C20 | 121.5(4) |
| C6 | C1 | C8 | 121.1(5) | C18 | C19 | C14 | 121.4(4) |
| C3 | C2 | C1 | 119.7(5) | N4 | C20 | C18 | 116.6(4) |
| C2 | C3 | C7 | 118.8(5) | N5 | C20 | N4 | 124.9(4) |
| C4 | C3 | C2 | 121.2(6) | N5 | C20 | C18 | 118.5(4) |
| C4 | C3 | C7 | 120.1(5) | N5 | C22 | C23 | 120.7(4) |
| C5 | C4 | C3 | 118.7(5) | O2 | C23 | C22 | 125.0(4) |
| C4 | C5 | C6 | 121.7(6) | O2 | C23 | C24 | 117.8(4) |
| C1 | C6 | C5 | 119.2(6) | C24 | C23 | C22 | 117.1(4) |
| N1 | C7 | C3 | 178.8(7) | N4 | C24 | C23 | 122.8(4) |

**Table S5 Bond Angles for hy0102.**

| Atom | Atom | Atom | Angle/° | Atom | Atom | Atom | Angle/° |
|---|---|---|---|---|---|---|---|
| N2 | C8 | C1 | 115.4(4) | O2 | C26 | C27 | 107.1(4) |
| N2 | C8 | C9 | 121.2(4) | C28 | C27 | C26 | 109.3(4) |
| C9 | C8 | C1 | 123.5(5) | C31 | C27 | C26 | 111.9(4) |
| C10 | C9 | C8 | 119.8(5) | C31 | C27 | C28 | 109.4(4) |
| C9 | C10 | C11 | 120.7(5) | C27 | C28 | C29 | 109.9(4) |
| O1 | C11 | N3 | 119.9(5) | N6 | C29 | C28 | 110.7(4) |
| O1 | C11 | C10 | 125.5(5) | N6 | C30 | C31 | 109.9(4) |
| N3 | C11 | C10 | 114.7(4) | C27 | C31 | C30 | 112.0(4) |

**Table S6 Torsion Angles for hy0102.**

| A | B | C | D | Angle/° | A | B | C | D | Angle/° |
|---|---|---|---|---|---|---|---|---|---|
| O2 | C23 | C24 | N4 | -177.3(5) | C12 | N3 | C11 | O1 | -3.2(7) |
| O2 | C26 | C27 | C28 | 175.2(4) | C12 | N3 | C11 | C10 | 176.1(4) |
| O2 | C26 | C27 | C31 | -63.5(5) | C12 | C14 | C15 | C16 | -177.4(5) |
| N2 | N3 | C11 | O1 | 176.5(4) | C12 | C14 | C19 | C18 | 178.0(5) |
| N2 | N3 | C11 | C10 | -4.2(7) | C13 | C12 | C14 | C15 | 101.1(5) |
| N2 | N3 | C12 | C13 | 43.7(5) | C13 | C12 | C14 | C19 | -74.9(6) |

**Table S6 Torsion Angles for hy0102.**

| A | B | C | D | Angle/° | A | B | C | D | Angle/° |
|---|---|---|---|---|---|---|---|---|---|
| N2 | N3 | C12 | C14 | -81.5(5) | C14 | C15 | C16 | C17 | 0.4(8) |
| N2 | C8 | C9 | C10 | -2.0(8) | C15 | C14 | C19 | C18 | 2.0(7) |
| N3 | N2 | C8 | C1 | -178.7(4) | C15 | C16 | C17 | C18 | -0.3(8) |
| N3 | N2 | C8 | C9 | 0.1(7) | C16 | C17 | C18 | C19 | 1.0(7) |
| N3 | C12 | C14 | C15 | -134.4(4) | C16 | C17 | C18 | C20 | -179.1(5) |
| N3 | C12 | C14 | C19 | 49.6(6) | C17 | C18 | C19 | C14 | -1.9(7) |
| N5 | C22 | C23 | O2 | 177.5(5) | C17 | C18 | C20 | N4 | -166.5(5) |
| N5 | C22 | C23 | C24 | -2.6(8) | C17 | C18 | C20 | N5 | 12.3(7) |
| N6 | C30 | C31 | C27 | 55.8(6) | C19 | C14 | C15 | C16 | -1.3(7) |
| C1 | C2 | C3 | C4 | -0.4(8) | C19 | C18 | C20 | N4 | 13.3(7) |
| C1 | C2 | C3 | C7 | 177.6(5) | C19 | C18 | C20 | N5 | -167.9(5) |
| C1 | C8 | C9 | C10 | 176.7(5) | C20 | N4 | C24 | C23 | -1.1(7) |
| C2 | C1 | C6 | C5 | 0.0(8) | C20 | N5 | C22 | C23 | 0.8(8) |
| C2 | C1 | C8 | N2 | 31.1(7) | C20 | C18 | C19 | C14 | 178.2(5) |
| C2 | C1 | C8 | C9 | -147.8(5) | C22 | N5 | C20 | N4 | 1.1(8) |
| C2 | C3 | C4 | C5 | -0.3(9) | C22 | N5 | C20 | C18 | -177.6(4) |
| C3 | C4 | C5 | C6 | 0.9(10) | C22 | C23 | C24 | N4 | 2.8(8) |
| C4 | C5 | C6 | C1 | -0.8(9) | C23 | O2 | C26 | C27 | -170.9(4) |

**Table S6 Torsion Angles for hy0102.**

| A | B | C | D | Angle/° | A | B | C | D | Angle/° |
|---|---|---|---|---|---|---|---|---|---|
| C6 | C1 | C2 | C3 | 0.5(8) | C24 | N4 | C20 | N5 | -1.0(8) |
| C6 | C1 | C8 | N2 | -147.4(5) | C24 | N4 | C20 | C18 | 177.8(4) |
| C6 | C1 | C8 | C9 | 33.8(8) | C26 | O2 | C23 | C22 | -7.5(7) |
| C7 | C3 | C4 | C5 | -178.3(6) | C26 | O2 | C23 | C24 | 172.6(4) |
| C8 | N2 | N3 | C11 | 3.2(7) | C26 | C27 | C28 | C29 | 179.7(4) |
| C8 | N2 | N3 | C12 | -177.2(4) | C26 | C27 | C31 | C30 | -177.4(4) |
| C8 | C1 | C2 | C3 | -177.9(5) | C27 | C28 | C29 | N6 | -59.0(5) |
| C8 | C1 | C6 | C5 | 178.5(5) | C28 | C27 | C31 | C30 | -56.1(5) |
| C8 | C9 | C10 | C11 | 0.8(8) | C29 | N6 | C30 | C31 | -56.8(5) |
| C9 | C10 | C11 | O1 | -178.7(5) | C30 | N6 | C29 | C28 | 59.3(5) |
| C9 | C10 | C11 | N3 | 2.1(7) | C31 | C27 | C28 | C29 | 56.9(5) |
| C11 | N3 | C12 | C13 | -136.6(4) | C32 | N6 | C29 | C28 | -176.8(4) |
| C11 | N3 | C12 | C14 | 98.2(5) | C32 | N6 | C30 | C31 | 179.1(4) |

**Table S7 Hydrogen Atom Coordinates (Å×$10^4$) and Isotropic Displacement Parameters (Å$^2$×$10^3$) for hy0102.**

| Atom | *x* | *y* | *z* | U(eq) |
|---|---|---|---|---|
| H6 | 8609 | 2194.44 | 346 | 32 |
| H2 | 4357.95 | 7128.57 | 5536.79 | 39 |
| H4 | 1894.45 | 6779.72 | 3484.62 | 60 |
| H5 | 1054.89 | 5602.33 | 4573.4 | 62 |
| H6A | 1826.11 | 5227.06 | 6156.49 | 50 |
| H9 | 2962.73 | 3752.61 | 7418.51 | 44 |
| H10 | 3869.38 | 3521.16 | 8925.88 | 46 |
| H12 | 5787.47 | 8528.87 | 9214.87 | 31 |
| H13A | 5670.46 | 11264.81 | 8432.01 | 47 |
| H13B | 4597.56 | 10512.89 | 8376.31 | 47 |
| H13C | 5096.14 | 10367.48 | 7490.62 | 47 |
| H15 | 7487.52 | 8688.84 | 9459.11 | 33 |
| H16 | 8957.61 | 8224.96 | 8989.15 | 36 |
| H17 | 8997.55 | 7387.75 | 7444.56 | 37 |
| H19 | 6020.04 | 7497.44 | 6818.06 | 31 |
| H22 | 8979.64 | 5300.52 | 4746.57 | 37 |
| H24 | 6116.24 | 6534.55 | 3980.49 | 33 |

**Table S7 Hydrogen Atom Coordinates (Å×$10^4$) and Isotropic Displacement Parameters (Å$^2$×$10^3$) for hy0102.**

| Atom | *x* | *y* | *z* | U(eq) |
|---|---|---|---|---|
| H26A | 8692.05 | 4007.86 | 3207.44 | 34 |
| H26B | 8911.9 | 6101.11 | 3189.73 | 34 |
| H27 | 7916.61 | 6285.2 | 1634.06 | 31 |
| H28A | 9501.7 | 3779.5 | 1785.16 | 31 |
| H28B | 9623.01 | 5895.69 | 1760.57 | 31 |
| H29A | 8685.18 | 5984.1 | 196.81 | 31 |
| H29B | 9597.72 | 4694.3 | 235 | 31 |
| H30A | 6913.67 | 2476.7 | 166.07 | 41 |
| H30B | 6996.12 | 4597.72 | 118.18 | 41 |
| H31A | 6885.6 | 3788.9 | 1669.59 | 38 |
| H31B | 7785.29 | 2460.39 | 1741.43 | 38 |
| H32A | 7798.14 | 4260.65 | -1268.2 | 60 |
| H32B | 7750.59 | 2142.41 | -1202.37 | 60 |
| H32C | 8768.24 | 3109.05 | -1172.5 | 60 |

**Table S8 Solvent masks information for hy0102.**

| Number | X | Y | Z | Volume | Electron count | Content |
|---|---|---|---|---|---|---|
| 1 | -0.006 | -0.078 | 0.340 | 39.5 | 10.0 | ? |
| 2 | 0.006 | 0.422 | 0.660 | 39.5 | 10.2 | ? |

## Experimental

Single crystals of $C_{30}H_{31}ClN_6O_2$ **[hy0102]**.A suitable crystal was selected and operated on a SuperNova, Dual, Cu at zero, AtlasS2 diffractometer. The crystal was kept at 169.99(10) K during data collection.
